# Positional Fluorination of Phenylpyridine: Unexpected Electronic Tuning in Bis-Cyclometalated Iridium(III) Acetylacetonate Complexes

**DOI:** 10.1021/acs.inorgchem.5c05152

**Published:** 2025-12-29

**Authors:** Silvia Sigismondi, Valentina Montani, Morgan Gaggioli, Daniele Tedesco, Nicola Armaroli, Letizia Sambri, Filippo Monti, Andrea Baschieri

**Affiliations:** † Institute for Organic Synthesis and Photoreactivity (ISOF), National Research Council of Italy (CNR), Via Piero Gobetti 101, 40129 Bologna, Italy; ‡ Department of Industrial Chemistry “Toso Montanari”, 9296 University of Bologna, Via Piero Gobetti 85, 40129 Bologna, Italy

## Abstract

We report a systematic study on the effect of positional fluorination of 2-phenylpyridine (Hppy) cyclometalating ligands in neutral iridium­(III) complexes of general formula [Ir­(Fppy)_2_(acac)], where Fppy = 2-(fluorophenyl)­pyridine and acac = acetylacetonate. Five complexes (**C1**–**C5**) were synthesized, including a nontrivial asymmetric derivative. The archetypal complexes [Ir­(ppy)_2_(acac)] (**C6**) and [Ir­(dFppy)_2_(acac)] (**C7**) were also prepared, to serve as reference. We demonstrate that fluorination does not simply induce a general blue shift, but that the specific substitution site on the phenyl ring of the cyclometalating ligands tunes HOMO and LUMO levels in a nontrivial fashion. Indeed, *para* fluorination (as in **C3**) affords the most red-shifted emission of the series, even compared to the fluorine free **C6** complex. On the other hand, *meta* and *ortho* substitutions (as in **C4** and **C2**, and **C1**, respectively) result in progressively bluer emission, in line with electrochemical and computational data. The asymmetric complex (**C5**) exhibits intermediate properties, reflecting the averaged contribution of the two parent symmetric complexes (**C1** and **C3**). This work establishes positional fluorination as a powerful design tool for tuning the electronic structure and emission color of cyclometalated iridium­(III) complexes, with relevance for photocatalytic applications, OLED development, and other photonic technologies.

## Graphical abstract

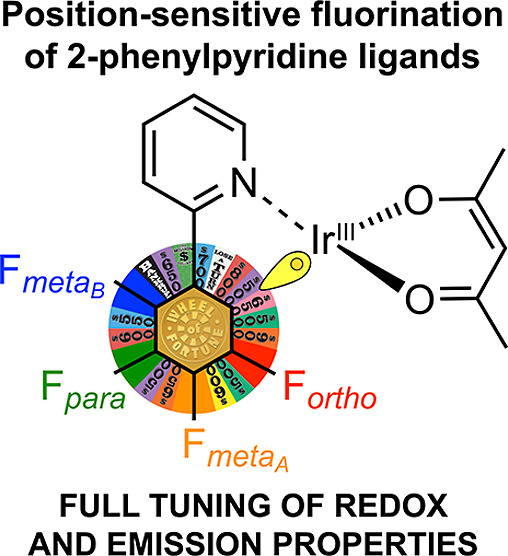

## Introduction

A comprehensive understanding of structure–property relationships in transition metal complexes is essential, given their critical roles in catalysis, optoelectronics, and sensing technologies.^1–6^

Among this vast class of compounds, cyclometalated iridium­(III) complexes are cornerstones, owing to their intense and long-lived phosphorescence, high thermal and photochemical stability, and easily tunable emission, all across the visible spectrum.^7–10^ Since the pioneering work by Thompson and co-workers in 1999, reporting an OLED incorporating the homoleptic cyclometalated iridium­(III) dopant *fac*-[Ir­(ppy)_3_] (where Hppy = 2-phenylpyridine),^11^ the field has expanded to include other countless tris-cyclometalated derivatives, with the general formula [Ir­(C^N)_3_],^12,13^ as well as their bis-cyclometalated heteroleptic analogues [Ir­(C^N)_2_(X^Y)]^0/+^.^9,14^ In the heteroleptic systems, the ancillary ligand (X^Y) may include neutral chelating donors such as diamines (N^N), phosphines (P^P), or carbenes (N^C), affording cationic complexes.^10^ Alternatively, anionic ligands such as β-diketonates (O^O^–^), picolinates (N^O^–^), and pyridineazolates (N^N^–^), bearing deprotonatable NH or OH groups, are typically employed to generate neutral complexes commonly used in OLEDs.^15^

Heteroleptic iridium­(III) complexes equipped with small ancillary ligands with high-energy π* orbitals, typically display emission from ^3^LC states, located on the cyclometalating ligands. Within this family, complexes of the type [Ir­(C^N)_2_(acac)] (where Hacac = 2,4-pentanedione or acetylacetone) are paradigmatic examples.^16^ In these systems, the HOMO and LUMO are located on different regions of the C^N ligands; for example, in complexes featuring ppy ligands, the HOMO is primarily localized on the phenyl ring and the iridium center, while the LUMO is mainly confined to the pyridine moiety.^17,18^

As a consequence, systematic chemical modifications of the phenylpyridine ligand provide a direct handle for tuning the HOMO–LUMO gap and, hence, the absorption, emission, and redox potentials of the related complexes.^15,19,20^ Introducing electron-withdrawing groups (*e.g*., −F or −CN) on the phenyl ring of the ppy cyclometalating ligands is known to stabilize the HOMO (and increase the HOMO–LUMO band gap), resulting in a blue shift of the complex emission.^21–23^ This is assumed to be true for all emitters of the type [Ir­(C^N)_2_(X^Y)]^0/+^,^24–27^ and the well-known FIrpic complex (*i.e.*, [Ir­(dFppy)_2_(pic)], where pic = picolinate) is just a notorious example,^28^ likewise [Ir­(dFppy)_2_(acac)].^29^

However, the above-mentioned studies have mostly varied the number of fluorine atoms rather than their precise position on the aromatic ring, and a systematic and rigorous mapping of positional effects is still lacking. Yet, substituent theories (*e.g.*, Hammett parameters) predicts that *ortho*, *meta*, and *para* fluorine substitutions, relative to the metal ion, should influence the complex electron density differently, suggesting opportunities for more refined and nonstraightforward electronic control.^25,30^

Here we fill this gap presenting a complete series of emissive neutral iridium­(III) complexes featuring specifically designed monofluorinated 2-phenylpiridine derivatives as cyclometalating ligands (HFppy, **L1**–**L3**) and the acetylacetonate as the ancillary one (Figure 1, left).

**Figure 1:**
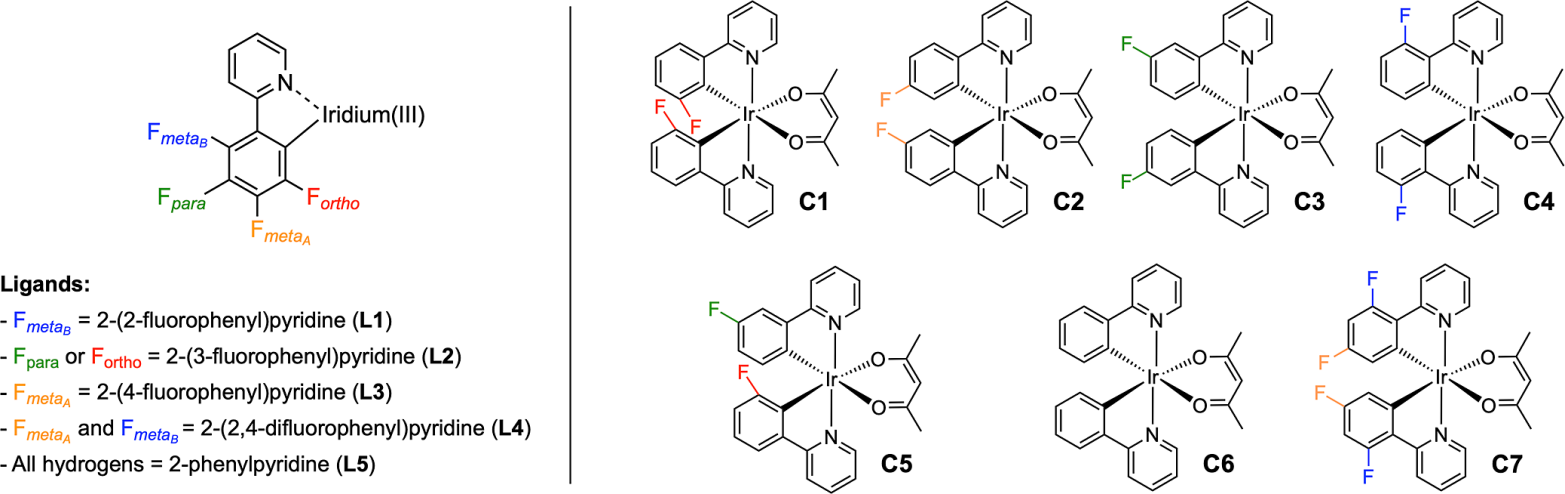
(Left) Mono-, bis- and nonfluorinated ppy-based ligands (**L1**–**L5**); (right) related neutral bis-cyclometalated iridium­(III) complexes (**C1**–**C7**) investigated in this work.

By fine-tuning the reaction conditions, we could selectively obtain all the 4 possible cyclometalated complexes (**C1**–**C4**) with the general formula [Ir­(Fppy)_2_(acac)], where Fppy = F_ortho_ (oFppy), F_metaA_ (m_A_Fppy), F_para_ (pFppy), and F_metaB_ (m_B_Fppy), relative to the iridium center (Figure 1, right). Such complexes incorporate a fluorine substituent at one of the four available positions on the phenyl ring of the two ppy ligands. Moreover, due to the different chelation modes of **L2**, the mixed asymmetric complex [Ir­(pFppy)­(oFppy)­(acac)] (**C5**) was also isolated and characterized. For completeness, the fluorine-free [Ir­(ppy)_2_(acac)] complex (**C6**), and the [Ir­(dFppy)_2_(acac)] complex (**C7**), bearing two fluorine atoms on the cyclometalating ligands, were synthesized and employed as references. The results of this positional fluorination study are presented and discussed below.

## Experimental Section

### General Information

Analytical grade solvents and commercially available reagents were used as received unless otherwise stated. Chromatographic purifications were performed using aluminum oxide 90 active neutral (activity stage I) 0.063–0.200 mm (70–230 mesh) or using silica gel 70–230 mesh. ^1^H, ^19^F, and ^13^C NMR spectra were recorded on Agilent (500 MHz for ^1^H) and Varian Mercury (400 MHz for ^1^H) spectrometers. Chemical shifts (δ) are reported in ppm relative to residual solvent signals for ^1^H and ^13^C NMR (^1^H NMR: 7.26 ppm for CDCl_3_, 5.33 ppm for CD_2_Cl_2_; ^13^C NMR: 77.0 ppm for CDCl_3_, 53.84 ppm for CD_2_Cl_2_. ^19^F NMR spectra were recorded at 470 MHz using trichlorofluoromethane as an external standard. ^13^C NMR spectra were acquired with the ^1^H broadband decoupled mode. Coupling constants are given in Hz. The abbreviations used to indicate the multiplicity of signals are: s, singlet; d, doublet; t, triplet; dd, double doublet; ddd, double double doublet; dt, double triplet; m, multiplet. The high-resolution mass spectra (HRMS) were obtained with an ESI-QTOF (Agilent Technologies, model G6520A) instrument, and the *m*/*z* values are referred to the monoisotopic mass.

Ligands **L1**, **L2** and **L3** were synthesized following a previously reported procedure with slight modifications;^31^ instead, ligands **L4** and **L5** were commercially available.

### General Procedures for the Synthesis of Ligands **L1**, **L2** and **L3**

To a two necked, 50 mL round-bottom flask equipped with a magnetic stir bar were added 2-chloropyridine (330 μL, 3.52 mmol, 1 equiv), phenylboronic acid (4.23 mmol, 1.2 equiv), triphenylphosphine (92 mg, 0.35 mmol, 0.1 equiv), 2 M in water potassium carbonate (1.31 g, 9.5 mmol, 2.7 equiv) and ethylene glycol dimethyl ether (5 mL). The mixture was degassed with N_2_ for 15 min. Then Pd­(OAc)_2_ (29.9 mg, 0.088 mmol, 0.025 equiv) was added to the reaction mixture and degassing continued for 15 more minutes and then the outlet was removed. The reaction mixture was heated to reflux. The progress of reaction was monitored by TLC. Upon completion (typically 24 h), reaction mixture was cooled to room temperature and then extracted with water (60 mL) and DCM (3 × 20 mL). The combined organic portion was dried over anhydrous sodium sulfate and then concentrated in vacuo. The crude material was purified by flash chromatography to obtain pure ligand.

NMR spectra of previously reported compounds were in agreement with those of the authentic samples and/or available literature data (see Figures S1–S3).

#### 2-(2-Fluorophenyl)­pyridine (**L1**)

The crude was purified by column chromatography on silica gel with cyclohexane/ethyl acetate (95:5) as eluent, to give the desired products. Results: 485.3 mg, 2.81 mmol, yield = 79.7%. ^1^H NMR (500 MHz, CDCl_3_): δ 8.71 (d, *J* = 3.9 Hz, 1H), 7.98 (td, *J* = 7.8, 1.9 Hz, 1H), 7.77 (d, *J* = 9.0 Hz, 1H), 7.74–7.66 (m, 1H), 7.39–7.31 (m, 1H), 7.25 (d, *J* = 7.6 Hz, 1H), 7.21 (d, *J* = 16.9 Hz, 1H), 7.17–7.10 (m, 1H).^32^

#### 2-(3-Fluorophenyl)­pyridine (**L2**)

The crude was purified by column chromatography on silica gel with hexane/ethyl acetate (95:5) as eluent, to give the desired products. Results: 561.2 mg, 3.24 mmol, yield = 92%. ^1^H NMR (400 MHz, CDCl_3_): δ 8.71–8.63 (m, 1H), 7.77–7.63 (m, 4H), 7.43–7.36 (m, 1H), 7.23–7.17 (m, 1H), 7.11–7.04 (m, 1H).^33^

#### 2-(4-Fluorophenyl)­pyridine (**L3**)

The crude was purified by column chromatography on silica gel with hexane/ethyl acetate (90:10) as eluent, to give the desired products. Results: 316.7 mg, 1.83 mmol, yield = 52%. ^1^H NMR (500 MHz, CDCl_3_): δ 8.64 (d, *J* = 4.9 Hz, 1H), 7.97 (dd, *J* = 8.9, 5.4 Hz, 2H), 7.67–7.56 (m, 2H), 7.09 (t, *J* = 8.7 Hz, 3H).^31^

#### General Procedures for the Synthesis of Dimers **Ir-dimer-1–Ir-dimer-4**

According to the Nonoyama route,^34^ cyclometalated iridium­(III) μ-chloro-bridged dimers were synthesized by charging a 50 mL round-bottom flask with magnetic stir bar, IrCl_3_·*x*H_2_O (90 mg, 0.26 mmol, 1 equiv), ligands **L1**, **L3**, **L4** or **L5** (0.65 mmol, 2.5 equiv), and a mixture 3:1 (v/v) of 2-ethoxyethanol/water (6 mL). The reaction was heated at 120 °C under nitrogen atmosphere with constant stirring overnight. The crude mixture was cooled to room temperature. The solution was added dropwise to 200 mL of water previously cooled in an ice bath. The precipitate was filtered and washed with water (1 × 10 mL) and ethyl ether (1 × 5 mL) and dried in air. The dimers were used in the next step without further purification.

#### Synthesis of Dimers **Ir-dimer-5**

Ligand **L2** (141 mg, 0.82 mmol, 2.3 equiv) was dissolved in a mixture of 2-ethoxyethanol/water 3:1 (8.0 mL), and the solution was degassed with N_2_ for 20 min. Then, IrCl_3_·*x*H_2_O (125 mg, 0.35 mmol, 1 equiv) was added and the resulting mixture was heated at 60 °C for 72 h or 120 °C for 24 h in both cases in a N_2_ atmosphere. Then, the reaction was cooled to room temperature, and the solution was added dropwise to 250 mL of water previously cooled in an ice bath. The precipitate was filtered and washed with water (2 × 10 mL) and ethyl ether (1 × 5 mL) and dried in air. Results for the reaction at 60 °C: 85.3 mg, 0.075 mmol, yield = 42.6%. Results for the reaction at 120 °C: 138.4 mg, 0.12 mmol, yield = 69.2%. The mixture of dimers was used in the next step without further purification.

Complexes **C1**–**C7** were synthesized following a previously reported procedure with slight modifications.^35^

#### General Procedures for the Synthesis of Complexes **C1–C7**

**Ir-dimer-1–Ir-dimer-5** (0.07 mmol, 1.0 equiv) was dissolved in dichloromethane (12.5 mL). Then, the ancillary ligand acetylacetone (16 μL, 0.154 mmol, 2.2 equiv) and K_2_CO_3_ (96 mg, 0.7 mmol, 10 equiv) were added and the mixture was stirred for 24 h at reflux under nitrogen atmosphere. Water (60 mL) was then added and the mixture was extracted with dichloromethane (3 × 20 mL). The organic layer was dried over Na_2_SO_4_ and the solvent evaporated.

#### Complex (**C1**, **C3** and **C5**)

To have enough amount of all isomers, two reactions were performed using **Ir-dimer-5** obtained at 60 and 120 °C, respectively. The reaction crudes were combined before purification and the mixture was purified by column chromatography on neutral aluminum oxide with hexane/ethyl acetate from 80:20 to 75:25 as eluent, to obtain a clean mixture of the three isomers without other impurities. The isomer ratio of the resulting **C1**/**C3**/**C5** mixtures was determined by analytical HPLC, while isolated complexes were then obtained by semipreparative HPLC (see HPLC methods for further details).

#### Complex (**C1**)

Results: 16.9 mg, 0.027 mmol, yield = 19.0%. ^1^H NMR (500 MHz, CDCl_3_): δ 8.45 (d, *J* = 5.7 Hz, 2H), 7.82 (d, *J* = 8.1 Hz, 2H), 7.76–7.67 (m, 2H), 7.45 (d, *J* = 7.7 Hz, 2H), 7.10 (t, *J* = 7.3 Hz, 2H), 6.87–6.77 (m, 2H), 6.35 (t, *J* = 9.1 Hz, 2H), 5.26 (s, 1H), 1.78 (s, 6H); ^13^C NMR (126 MHz, CDCl_3_): δ 184.4 (CO), 170.1 (d, *J* = 235.9 Hz, C), 168.4 (C), 149.4 (d, *J* = 16.0 Hz, C), 148.7 (CH), 136.7 (CH), 126.7 (d, *J* = 36.8 Hz, C), 122.3 (d, *J* = 7.4 Hz, CH), 121.1 (CH), 119.6 (d, *J* = 2.8 Hz, CH), 118.5 (CH), 115.6 (d, *J* = 27.3 Hz, CH), 100.5 (CH), 28.6 (CH_3_); ^19^F NMR (470 MHz, CDCl_3_): δ −106.15 (dd, *J* = 9.1, 5.2 Hz). HRMS (ESI-QTOF) *m*/*z*: calcd for C_27_H_21_F_2_IrN_2_O_2_, 635.1250; found, 635.1272 [M + H]^+^

#### Complex (**C3**)

Results: 34.4 mg, 0.054 mmol, yield = 38.7%. ^1^H NMR (500 MHz, CDCl_3_): δ 8.50 (d, *J* = 5.6 Hz, 2H), 7.83–7.72 (m, 4H), 7.27 (d, *J* = 2.7 Hz, 2H), 7.21–7.15 (m, 2H), 6.55–6.46 (m, 2H), 6.16–6.08 (m, 2H), 5.22 (s, 1H), 1.79 (s, 6H); ^13^C NMR (126 MHz, CDCl_3_): δ 184.7 (CO), 167.7 (d, *J* = 4.4 Hz, C), 159.4 (d, *J* = 235.7 Hz, C), 148.3 (CH), 145.2 (d, *J* = 6.6 Hz, C), 140.1 (d, *J* = 2.4 Hz, C), 137.1 (CH), 133.3 (d, *J* = 6.6 Hz, CH), 122.0 (CH), 118.7 (CH), 116.4 (d, *J* = 19.6 Hz, CH), 110.5 (d, *J* = 21.3 Hz, CH), 100.5 (CH), 28.8 (CH_3_); ^19^F NMR (470 MHz, CDCl_3_): δ −124.53 (td, *J* = 9.9, 5.8 Hz). HRMS (ESI-QTOF): *m*/*z* calcd for C_27_H_21_F_2_IrN_2_O_2_, 635.1250; found, 635.1282 [M + H]^+^

#### Complex (**C5**)

Results: 28.8 mg, 0.045 mmol, yield = 32.4%. ^1^H NMR (500 MHz, CDCl_3_): δ 8.49 (s, 1H), 8.46 (d, *J* = 4.9 Hz, 1H), 7.89 (s, 1H), 7.80–7.69 (m, 3H), 7.45 (d, *J* = 7.6 Hz, 1H), 7.28 (d, *J* = 2.7 Hz, 1H), 7.18–7.09 (m, 2H), 6.85–6.77 (m, 1H), 6.55–6.46 (m, 1H), 6.34 (t, *J* = 9.0 Hz, 1H), 6.07 (dd, *J* = 8.4, 6.1 Hz, 1H), 5.24 (s, 1H), 1.81 (s, 3H), 1.77 (s, 3H); ^13^C NMR (126 MHz, CDCl_3_): δ 184.9 (CO), 184.2 (CO), 170.2 (d, *J* = 236.0 Hz, C), 168.3 (d, *J* = 1.5 Hz, C), 167.6 (d, *J* = 4.4 Hz, C), 159.7 (d, *J* = 235.7 Hz, C), 149.1 (CH), 148.5 (d, *J* = 16.5 Hz, C), 148.2 (CH), 146.1 (d, *J* = 6.6 Hz, C), 137.6 (d, *J* = 2.4 Hz, C), 137.0 (CH), 136.8 (CH), 133.2 (d, *J* = 6.6 Hz, CH), 129.4 (d, *J* = 37.6 Hz, C), 122.2 (d, *J* = 7.5 Hz, CH), 121.9 (CH), 121.2 (CH), 120.0 (d, *J* = 2.5 Hz, CH), 119.2 (CH), 118.1 (CH), 116.0 (d, *J* = 5.8 Hz, CH), 115.8 (d, *J* = 13.5 Hz, CH), 110.2 (d, *J* = 21.5 Hz, CH), 100.5 (CH), 28.7 (2CH_3_); ^19^F NMR (470 MHz, CDCl_3_): δ −105.36 (dd, *J* = 9.1, 5.0 Hz), −124.55 (td, *J* = 9.5, 6.2 Hz). HRMS (ESI-QTOF): *m*/*z* calcd for C_27_H_21_F_2_IrN_2_O_2_, 635.1250; found, 635.1279 [M + H]^+^

#### Complex (**C2**)

The crude was washed with hexane and acetonitrile. Results: 29.0 mg, 0.046 mmol, yield = 32.6%. ^1^H NMR (500 MHz, CD_2_Cl_2_): δ 8.44 (d, *J* = 7.2 Hz, 2H), 7.88–7.78 (m, 4H), 7.60 (dd, *J* = 8.6, 5.6 Hz, 2H), 7.22 (t, *J* = 7.3 Hz, 2H), 6.64–6.56 (m, 2H), 5.87 (dd, *J* = 9.8, 2.6 Hz, 2H), 5.31 (s, 1H), 1.82 (s, 6H); ^13^C NMR (126 MHz, CD_2_Cl_2_): δ 184. (CO), 167.1 (C), 162.7 (d, *J* = 251.9 Hz, C), 150.4 (C), 148.1 (CH), 141.4 (C), 137.5 (CH), 125.5 (d, *J* = 9.5 Hz, CH), 121.8 (CH), 118.9 (d, *J* = 16.8 Hz, CH), 118.6 (CH), 107.9 (d, *J* = 23.4 Hz, CH), 100.4 (CH), 28.2 (CH_3_); ^19^F NMR (470 MHz, CD_2_Cl_2_): δ −111.96 (td, *J* = 9.1, 5.4 Hz). HRMS (ESI-QTOF): *m*/*z* calcd for C_27_H_21_F_2_IrN_2_O_2_, 635.1250; found, 635.1261 [M + H]^+^

#### Complex (**C4**)

The crude was washed with hexane and acetonitrile. Results: 40.4 mg, 0.064 mmol, yield = 45.4%. Results: 73.5 mg, 0.122 mmol, yield = 87.5%. ^1^H NMR (500 MHz, CD_2_Cl_2_): δ 8.52 (d, *J* = 4.0 Hz, 2H), 8.32 (d, *J* = 8.2 Hz, 2H), 7.84 (t, *J* = 8.3 Hz, 2H), 7.24 (t, *J* = 7.2 Hz, 2H), 6.74–6.64 (m, 2H), 6.61–6.51 (m, 2H), 6.02 (d, *J* = 7.8 Hz, 2H), 5.31 (s, 1H), 1.82 (s, 6H); ^13^C NMR (126 MHz, CD_2_Cl_2_): δ 184.9 (CO), 166.6 (d, *J* = 230.7 Hz, C), 161.7 (C), 150.0 (C), 148.3 (CH), 139.5 (C), 137.7 (CH), 129.8 (d, *J* = 8.9 Hz, CH), 128.7 (d, *J* = 3.3 Hz, CH), 123.1 (d, *J* = 19.6 Hz, CH), 122.0 (CH), 108.1 (d, *J* = 22.4 Hz, CH), 100.4 (CH), 28.2 (CH_3_); ^19^F NMR (470 MHz, CD_2_Cl_2_): δ −114.71 (dd, *J* = 13.7, 7.0 Hz). HRMS (ESI-QTOF): *m*/*z* calcd for C_27_H_21_F_2_IrN_2_O_2_, 635.1250; found, 635.1275 [M + H]^+^

#### Complex (**C6**)

The crude was purified by column chromatography on neutral aluminum oxide with hexane/ethyl acetate 95:5 as eluent, to give the desired products. Results: 73.5 mg, 0.122 mmol, yield = 87.5%. ^1^H NMR (500 MHz, CDCl_3_): δ 8.51 (d, *J* = 4.9 Hz, 2H), 7.84 (d, *J* = 8.3 Hz, 2H), 7.77–7.68 (m, 2H), 7.54 (d, *J* = 9.2 Hz, 2H), 7.13 (t, *J* = 7.3 Hz, 2H), 6.80 (t, *J* = 8.1 Hz, 2H), 6.69 (t, *J* = 8.1 Hz, 2H), 6.27 (d, *J* = 7.7 Hz, 2H), 5.21 (s, 1H), 1.78 (s, 6H); ^13^C NMR (126 MHz, CDCl_3_): δ 184.6 (CO), 168.6 (C), 148.2 (CH), 147.6 (C), 144.7 (C), 136.8 (CH), 133.0 (CH), 129.1 (CH), 123.8 (CH), 121.4 (CH), 120.7 (CH), 118.4 (CH), 100.3 (CH), 28.7 (CH_3_).

#### Complex (**C7**)

The crude was purified by column chromatography on neutral aluminum oxide with cyclohexane/ethyl acetate from 98:2 to 95:5 as eluent, to give the desired products. Results: 42.9 mg, 0.064 mmol, yield = 45.6%. ^1^H NMR (500 MHz, CDCl_3_): δ 8.45 (d, *J* = 5.6 Hz, 2H), 8.25 (d, *J* = 9.0 Hz, 2H), 7.79 (t, *J* = 7.0 Hz, 2H), 7.18 (t, *J* = 5.8 Hz, 2H), 6.39–6.29 (m, 2H), 5.66 (dd, *J* = 8.8, 2.3 Hz, 2H), 5.26 (s, 1H), 1.81 (s, 6H); ^13^C NMR (126 MHz, CDCl_3_): δ 185.0 (CO), 165.31 (d, *J* = 7.2 Hz, C), 162.8 (dd, *J* = 230.3, 12.9 Hz, C), 160.7 (dd, *J* = 233.7, 12.9 Hz, C), 151.3 (d, *J* = 7.2 Hz, C), 148.0 (CH), 137.9 (CH), 128.6 (m, C), 122.6 (d, *J* = 19.5 Hz, CH), 121.6 (CH), 115.1 (dd, *J* = 16.7, 2.9 Hz, CH), 100.7 (CH), 97.3 (t, *J* = 26.9 Hz, CH), 28.7 (CH_3_); ^19^F NMR (470 MHz, CDCl_3_): δ −108.90 (q, *J* = 9.5 Hz), −111.17 (t, *J* = 12.4 Hz).

### Analytical and Semipreparative HPLC

The **C1**/**C3**/**C5** mixtures, as obtained by column chromatography purification, were subjected to reverse-phase HPLC on a Phenomenex Gemini C18 column (100 × 3.0 mm I.D., 3 μm particle size, 110 Å pore size), using a Shimadzu Nexera XR HPLC system with UV detection (λ = 254 nm). Water (A) and acetonitrile (B) were employed as mobile phase solvents (0.5 mL/min flow rate) according to the following gradient program: 50% B (0–1 min); from 50% to 85% B (1–8 min); 85% B (8–11 min); 50% B (11–15 min). The column temperature was kept constant at 40 °C and the injection volume was set to 5 μL, using 100 μg/mL samples in acetonitrile. Isomer ratios were estimated from chromatograms based on peak areas at 254 nm.

The isolation of **C1**, **C3** and **C5** was achieved by semipreparative HPLC purification on a Phenomenex Luna C18(2) AXIA column (250 × 21.2 mm I.D., 5 μm particle size, 100 Å pore size), using an Agilent 1260 Infinity II HPLC system with UV detection (λ = 254 nm). A water/acetonitrile mixture (35:65, v/v) was employed as mobile phase in isocratic conditions (20 mL/min flow rate). Reaction crudes were first purified by column chromatography using neutral alumina as the stationary phase to eliminate most of the impurities, then injected manually (1 mL for each injection) to isolate the isomers. The collected **C1**, **C3** and **C5** fractions were subsequently concentrated under reduced pressure using rotary evaporation; the resulting aqueous residue was extracted twice with dichloromethane, and the organic layers were dried over anhydrous sodium sulfate, filtered, and concentrated under reduced pressure.

### Electrochemical Characterization

Voltammetric experiments were performed using a Metrohm AutoLab PGSTAT 302N electrochemical workstation in combination with the NOVA 2.0 software package. All the measurements were carried out at room temperature in acetonitrile solutions with a sample concentration approximately 1.0 mM and using 0.1 M tetrabutylammonium hexafluorophosphate (electrochemical grade, TBAPF_6_) as the supporting electrolyte. Oxygen was removed from the solutions by bubbling argon. All the experiments were carried out using a three-electrode setup (BioLogic VC-4 cell, volume range: 1–3 mL) using a glassy carbon working electrode (having an active surface disk of 1.6 mm in diameter), the Ag/AgNO_3_ redox couple (0.01 M in acetonitrile, with 0.1 M TBAClO_4_ supporting electrolyte) as the reference electrode, and a platinum wire as the counter electrode. At the end of each measurement, ferrocene was added as the internal reference. Cyclic voltammograms (CV) were recorded at a scan rate of 100 mV s^–1^. Osteryoung square-wave voltammograms (OSWV) were recorded with scan rate of 25 mV s^–1^, a SW amplitude of ±20 mV, and a frequency of 25 Hz.

### Photophysics

The spectroscopic investigations were carried out in spectrofluorimetric grade acetonitrile. The absorption spectra were recorded with a PerkinElmer Lambda 950 spectrophotometer. For the photoluminescence experiments, the sample solutions were placed in fluorimetric Suprasil quartz cuvettes (10.00 mm) and dissolved oxygen was removed by bubbling argon for 30 min. The uncorrected emission spectra were obtained with an Edinburgh Instruments FLS920 spectrometer equipped with a Peltier-cooled Hamamatsu R928 photomultiplier tube (PMT, spectral window: 185–850 nm). An Osram XBO xenon arc lamp (450 W) was used as the excitation light source. The corrected spectra were acquired by means of a calibration curve, obtained by using an Ocean Optics deuterium–halogen calibrated lamp (DH-3plus-CAL-EXT). The photoluminescence quantum yields (PLQYs) in solution were obtained from the corrected spectra on a wavelength scale (nm) and measured according to the approach described by Demas and Crosby,^36^ using an air-equilibrated water solution of tris­(2,2′-bipyridyl)­ruthenium­(II) dichloride as reference (PLQY = 0.040).^37^ The emission lifetimes (τ) were measured through the time-correlated single photon counting (TCSPC) technique using an HORIBA Jobin Yvon IBH FluoroHub controlling a spectrometer equipped with a pulsed NanoLED (λ_exc_ = 465 nm) or SpectraLED (λ_exc_ = 370 nm) as the excitation source and a red-sensitive Hamamatsu R-3237–01 PMT (185–850 nm) as the detector. The analysis of the luminescence decay profiles was accomplished with the DAS6 Decay Analysis Software provided by the manufacturer, and the quality of the fit was assessed with the χ^2^ value close to unity and with the residuals regularly distributed along the time axis. To record the 77 K luminescence spectra, samples were put in quartz tubes (2 mm inner diameter) and inserted into a special quartz Dewar flask filled with liquid nitrogen. The poly­(methyl methacrylate) (PMMA) films containing 1% (w/w) of the complex were obtained by drop casting and the thickness of the films was not controlled. Solid-state PLQY values were calculated by corrected emission spectra obtained from an Edinburgh FLS920 spectrometer equipped with a barium sulfate- coated integrating sphere (diameter of 4 in.) following the procedure described by Würth et al.^38^ Experimental uncertainties are estimated to be ±8% for τ determinations, ±10% for PLQYs, ±2 nm and ±5 nm for absorption and emission peaks, respectively.

### Computational Details

Density functional theory (DFT) calculations were carried out using the B.01 revision of the Gaussian 16 program package^39^ in combination with the M06 global-hybrid *meta*-GGA exchange–correlation functional.^40,41^ The fully relativistic Stuttgart/Cologne energy-consistent pseudopotential with multielectron fit was used to replace the first 60 inner-core electrons of the iridium metal center (i.e., ECP60MDF) and was combined with the associated triple-ζ basis set (i.e., cc-pVTZ-PP basis).^42^ On the other hand, the Pople 6-31G­(d,p) basis was adopted for all other atoms.^43,44^ All the reported complexes were fully optimized without symmetry constraints, using a time-independent DFT approach, in their ground state (S_0_) and lowest triplet states; all the optimization procedures were performed using the polarizable continuum model (PCM) to simulate acetonitrile solvation effects.^45–47^ Frequency calculations were always used to confirm that every stationary point found by geometry optimizations was actually a minimum on the corresponding potential-energy surface (no imaginary frequencies). To investigate the nature of the emitting states, geometry optimizations and frequency calculations were performed at the spin-unrestricted UM06 level of theory (imposing a spin multiplicity of 3), using the S_0_ minimum-energy geometry as initial guess or other educated guesses. Time-dependent DFT calculations (TD-DFT),^48,49^ carried out at the same level of theory used for geometry optimizations, were used to calculate the first 16 triplet excitations and their nature was assessed with the support of natural transition orbital (NTO) analysis.^50^ All the pictures showing molecular geometries, orbitals and spin-density surfaces were created using GaussView 6.^51^

## Results and Discussion

### Synthesis

Ligands **L1**–**L3** were easily obtained through a typical Suzuki reaction, starting from commercially available 2-chloropyridine and differently substituted phenylboronic acids (Scheme 1).^31^ Conversely, ligands **L4** and **L5** were commercially available.

**Scheme 1:**
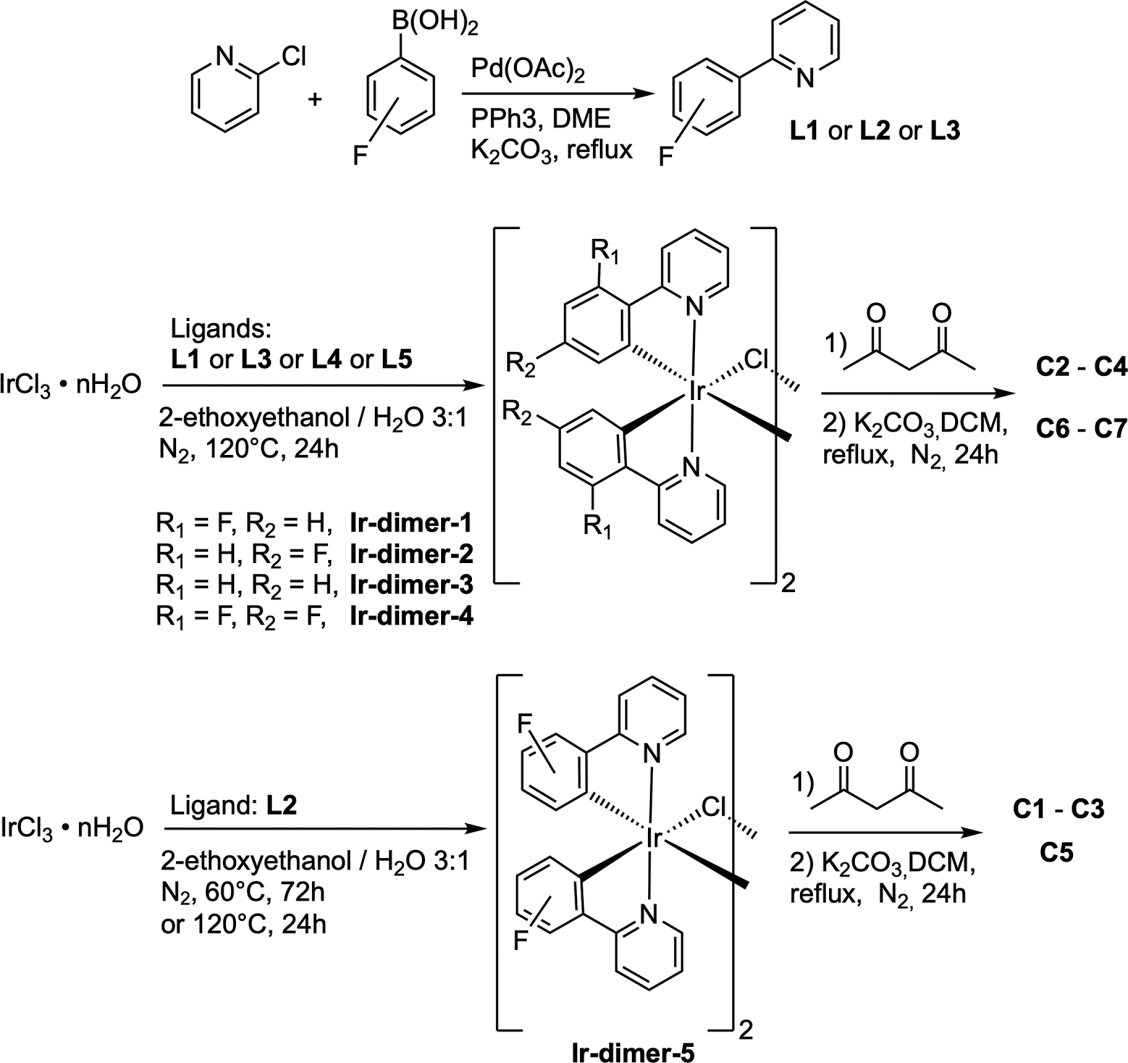
Synthesis of the Fluorinated Ligands **L1**–**L3** and of the Related Neutral Iridium­(III) Complexes (**C1**–**C7**)

To set up a suitable procedure to get complexes **C2**, **C4**, **C6** and **C7**, we prepared cyclometalated μ-dichloro bridged iridium precursors [Ir­(C^∧^N)_2_Cl]_2_ (**Ir-dimer-1**–**Ir-dimer-4**) following standard procedures by refluxing in a 2-ethoxyethanol/water (3:1) mixture the iridium­(III) chloride hydrate (IrCl_3_·*x*H_2_O) salt and the appropriate cyclometalating ligand HC^∧^N (**L1**, **L3, L4**, **L5**), as reported in Scheme 1.^34,52^

Chloro-bridged dimers thus obtained were reacted with acetylacetone in the presence of a base and the reaction mixture was stirred at reflux for 24 h. The crude was purified by column chromatography on neutral alumina. For complexes **C2** and **C4** the crude was simply washed with acetonitrile. Lastly, a final wash with hexane gave the desired pure complexes in good yields (32–87%), which were fully characterized by NMR spectroscopy (see the Experimental Section and Figures S4–S35).

However, the case of **L2** displayed a more complicated situation. In fact, this ligand presents two nonequivalent CH where cyclometalation with the iridium­(III) atom can occur.

For all other previously used fluorinated ligands (and nonfluorinated **L5**), cyclometalation could occur at only one position or at two equivalent positions (as in paths A and C, or path B, respectively, see Figure 2). In contrast, **L2**, which has a fluorine atom at the 3-position of the phenyl ring, *meta* to the heterocycle, offers two nonequivalent positions where cyclometalation can occur and can, in principle, lead to two different products (path D, Figure 2).

**Figure 2:**
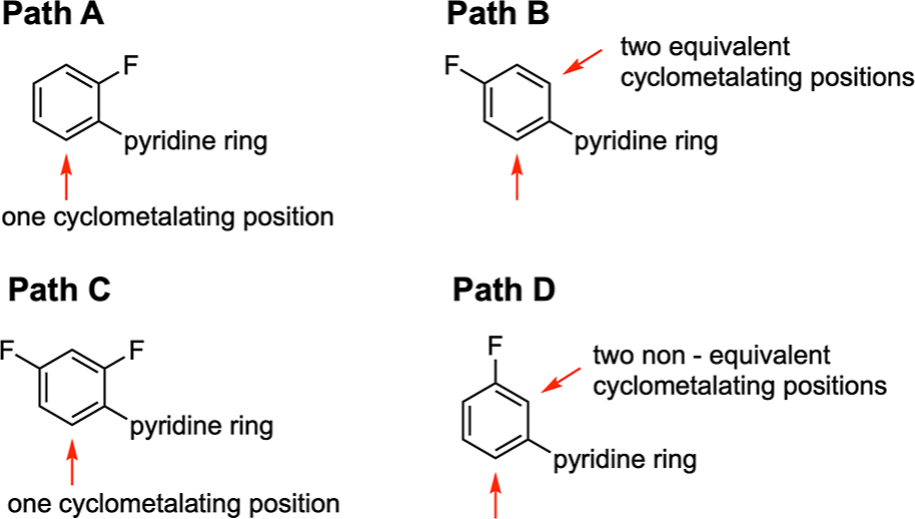
Possible sites for cyclometalation of *ortho*, *para* and *meta*-fluorinated phenylpyridine. The different positional fluorination may lead to only one (paths A and C) or two possible equivalent (path B) or nonequivalent (path D) cyclometalating positions.

Several examples have been reported in the literature in which *meta*-substituted phenylpyridine ligands are used to obtain cyclometalated iridium­(III) complexes. In almost all cases, the authors described the synthesis of complexes exhibiting only C_2_-symmetry.^24,53–56^

Only in very few studies the presence of minor isomers has been noted; however, the different compounds could not be separated and were therefore considered byproducts of the reaction.^57,58^ Recently, in 2023, Zhao and co-workers developed a “random cyclometalation” approach using 2-(3-methoxyphenyl)-4-(trifluoromethyl)­pyridine as a ligand, obtaining all three possible iridium­(III) complex isomers in a one-pot reaction.^59^

In our case, we initially adopted the classical procedure for the synthesis of iridium­(III) dimers—previously used for the preparation of complexes **C2**, **C4**, **C6**, and **C7**—to obtain **Ir-dimer-5**, using a reaction temperature of 120 °C. **Ir-dimer-5** does not represent a single compound, but rather a mixture of all possible isomers with the general formula [(**L2**)_2_Ir­(μ-Cl)]_2_ (a total of seven isomers are presumed). This compound was then used as-is, without further purification or separation steps, and reacted with the ancillary ligand acetylacetonate. This step reduces the number of possible isomers to three, thereby facilitating the identification of the reaction products.

The reaction can yield two symmetric complexes, **C1** and **C3**, or the asymmetric complex **C5**, in which the cyclometalating ligand is coordinated to the central iridium atom through two different binding modes. However, all three complexes exhibit only a singlet in the ^1^H NMR spectrum (around 5.25 ppm), attributable to the CH proton of the ancillary ligand. Unlike the aromatic region, where multiple signals from the three complex structures overlap, the CH signal appears in a more isolated region, making it possible to determine the number of products formed and to estimate their relative ratios in the reaction mixture crude. At 120 °C, the reaction afforded an isomeric ratio of 3.7:1:2.9 (Figure S36), which was later assigned to complexes **C1**, **C3**, and **C5**, respectively (see below).

Despite the fluorine atom having similar steric hindrance to a proton, we modified the reaction conditions to assess any change in the isomer ratio. We then attempted to synthesize **Ir-dimer-5** at 60 °C instead of 120 °C. To maintain a comparable yield, the reaction time was extended 3-fold. Since the attachment of the ancillary ligand is carried out at approximately 40 °C, it does not affect the binding mode of cyclometalation. Surprisingly, under these conditions, a 1:16.9:11.6 ratio of the **C1**, **C3**, and **C5** isomers was obtained (Figure S37).

Attempts to separate the isomers by column chromatography were unsuccessful. Based on the chromatographic resolution of isomers obtained in reverse-phase HPLC analysis, we resorted to semipreparative HPLC on a C18 column to collect pure isomeric fractions (Figures S38 and S39); in both experimental settings, the elution order (**C1** < **C5** < **C3**) was maintained. The HPLC estimation of **C1**:**C3**:**C5** ratios for the two different reaction conditions (2.4:1:2.6 at 120 °C, 1:12.8:9.6 at 60 °C) is in good agreement with NMR data.

Identification of the complex **C5** was straightforward, as its asymmetric structure is easily distinguishable by NMR spectroscopy (Figure S24). Identifying the **C1** and **C3** complexes was more challenging. To do this, we performed two-dimensional COSY NMR analysis, and the ^13^C NMR spectra of the two compounds were also recorded. In particular, by examining the C–F coupling constants in the DEPT-135 NMR spectra, it is possible to determine the position of the fluorine atom on the phenyl ring of the cyclometalating ligand, thereby differentiating **C1** from **C3**.

The **C1** complex exhibits three distinct C–F coupling constants, as the distances between the fluorine atom and the CH groups on the same ring are all different. In contrast, the **C3** complex has two CH groups on the phenyl ring that are equidistant from the fluorine atom, and thus it is expected to show two similar coupling constants and one smaller one (Figure 3).

**Figure 3:**
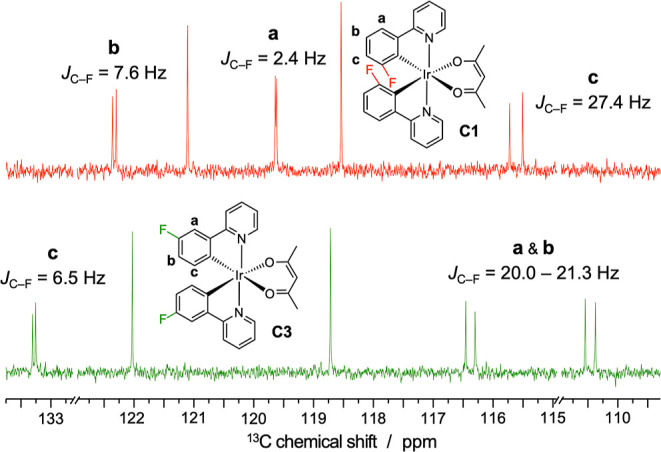
Portion of the DEPT-135 ^13^C NMR spectra of the **C1** and **C3** complexes, showing the corresponding C–F coupling constants.

### DFT Calculations: Ground-State Properties

To gain insight into the electronic structure and optical behavior of complexes **C1**–**C7**, we performed DFT and TD-DFT calculations employing the M06 hybrid *meta*-GGA exchange–correlation functional.^40,41^ All geometries were fully optimized in their ground state (S_0_), incorporating solvation effects from acetonitrile through the polarizable continuum model (PCM).^45–47^ The reliability of this computational strategy has been corroborated by prior studies on structurally related systems, as documented in the literature.^10,60^

Figure 4 depicts the energy diagram and frontier molecular orbitals for the present set of complexes. In line with other cyclometalated iridium­(III) complexes containing phenylpyridine-based cyclometalating ligands and an acetylacetonate as the ancillary one, the HOMO in each complex is primarily distributed over the iridium *d* orbitals and the phenyl units of the substituted cyclometalating ligands.^61^ As expected, the fluorine-free complex [Ir­(ppy)_2_(acac)] (**C6**) displays the highest HOMO, while the [Ir­(dFppy)_2_(acac)] counterpart (**C7**) has the most stabilized HOMO due to the presence of two fluorine substituent on each cyclometalating ligand (i.e., a 0.32 eV stabilization is observed compared to **C6**, Figure 4). However, depending on the fluorine position on the monofluorinated phenylpyridine ligands, the HOMO energy of the related complex could be finely tuned. Notably, in complex [Ir­(pFppy)_2_(acac)] (**C3**), the fluorine substituents have virtually no effects on the HOMO energy, which remains isoenergetic to that of the fluorine-free **C6** (Figure 4).

**Figure 4:**
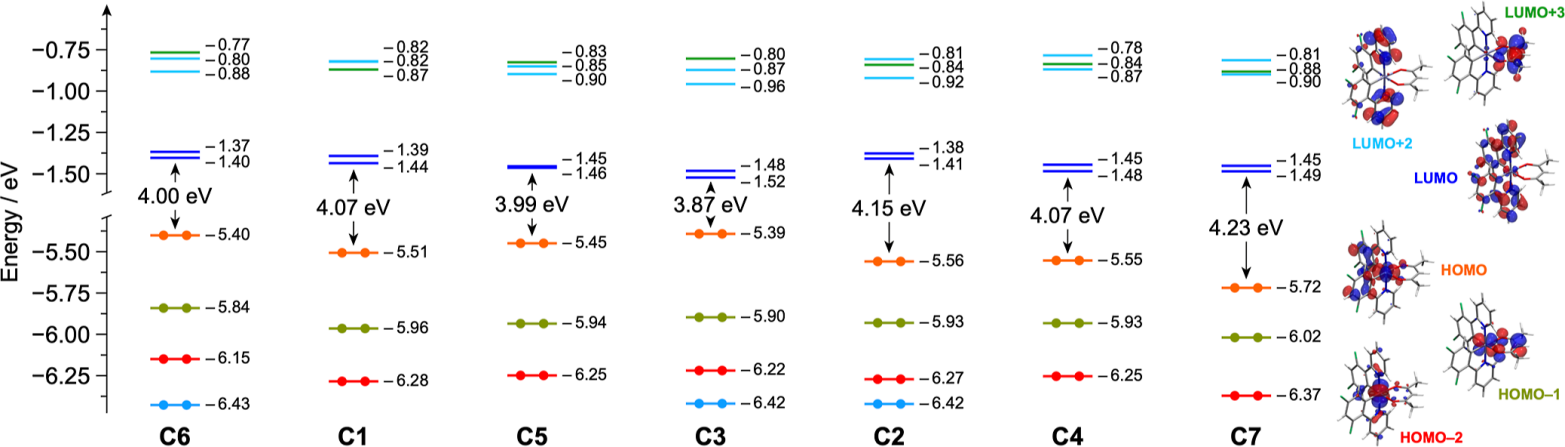
Energy diagram showing the energy values of the frontier Kohn–Sham molecular orbitals of **C1**–**C7** in acetonitrile. For the archetypal complex [Ir­(ppy)_2_(acac)] (**C6**), the frontier molecular orbitals are displayed for the sake of clarity (isovalue = 0.04 e^1/2^ bohr^–3/2^). Along the series, relevant orbitals with similar topology are plotted with the same color for an easier comparison.

In all complexes, the LUMO is mainly centered on the π* orbitals of the pyridine ring of the cyclometalating ligands, its energy being indirectly modulated by the different fluorine-substitution on the nearby phenyl moiety (Figure 4). As in the HOMO case, the most destabilized LUMO is observed for the fluorine-free complex **C6**, reflecting the absence of electron-withdrawing groups. Yet, despite complex **C7** contains the largest number of fluorine substituents, it does not exhibit the lowest LUMO of the series. Instead, complex [Ir­(m_A_Fppy)_2_(acac)] (**C2**), which contains only one fluorine per cyclometalating ligand, has a nearly isoenergetic LUMO. Even more surprisingly, [Ir­(pFppy)_2_(acac)] (**C3**) shows the lowest LUMO energy among the series, being 0.03 eV lower than **C7** and 0.12 eV below that of **C6**.

As expected, the largest HOMO–LUMO gap of the series is found in complex **C7** (i.e., 4.23 eV, Figure 4). However, counterintuitively, the smallest gap does not belong to the nonfluorinated complex **C6** (4.00 eV), but rather to complex **C3**, which shows a gap of just 3.87 eV (Figure 4). A narrowed gap is also observed in the tris–heteroleptic complex [Ir­(pFppy)­(oFppy)­(acac)] (**C5**), whose HOMO and LUMO energies fall between those of the parent counterparts **C1** and **C3**, resulting in a HOMO–LUMO gap virtually identical to that of the fluorine-free complex **C6** (Figure 4).

### Electrochemistry

To investigate the influence of the different fluorine substitution pattern on the electronic properties of the related cyclometalated iridium­(III) complexes, cyclic and square-wave voltammetry experiments were performed in acetonitrile at 298 K; the corresponding voltammograms are shown in Figures 5 and S40, respectively. The recorded redox potentials are summarized in Table 1, relative to the ferrocene/ferrocenium (Fc/Fc^+^) reference couple (see Experimental Section for further details).

**Figure 5:**
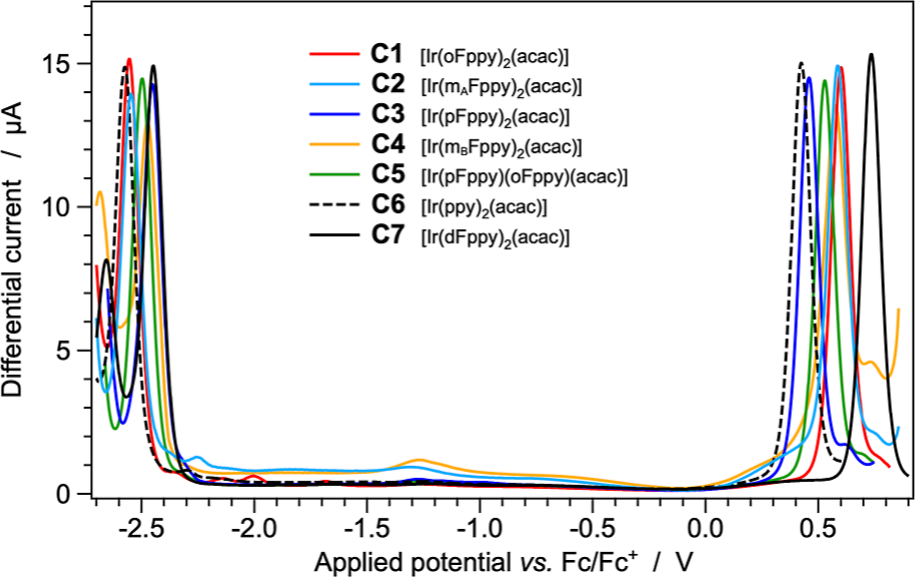
Square-wave voltammograms of complexes **C1**–**C7** (1.0 mM) in acetonitrile solution at 298 K.

**Table 1:** Electrochemical Data of **C1**–**C7** in Acetonitrile Solution (1.0 mM) + 0.1 M TBAPF_6_ at 298 K

	*E*_ox_ ^a^ [V]	*E*_red_ ^a^ [V]	Δ*E*_redox_ ^b^ [V]
**C1** [Ir(oFppy)_2_(acac)]	+0.600	–2.554	3.154
**C2** [Ir(m_A_Fppy)_2_(acac)]	+0.587	–2.546	3.133
**C3** [Ir(pFppy)_2_(acac)]	+0.458	–2.452	2.910
**C4** [Ir(m_B_Fppy)_2_(acac)]	+0.576	–2.473	3.049
**C5** [Ir(pFppy)(oFppy)(acac)]	+0.529	–2.497	3.026
**C6** [Ir(ppy)_2_(acac)]	+0.426	–2.574	3.000
**C7** [Ir(dFppy)_2_(acac)]	+0.737	–2.448	3.185

a The reported potential values are obtained by square-wave voltammetry and reported vs. the ferrocene/ferrocenium couple, used as internal reference. All redox processes are fully reversible.

b Δ*E*_redox_ = *E*_ox_ – *E*_red_.

For the entire set of investigated compounds, all the recorded redox processes are fully reversible (Figure 5); moreover, as suggested by DFT calculations (see previous section), both the oxidation and reduction reactions involve similar processes for all the complexes. Namely, the oxidation can be formally attributed to the Ir­(III)/Ir­(IV) redox couple, while the reduction involves the pyridine moieties of the cyclometalating ligands.^15^ Therefore, along the series, all redox potentials are affected by the different fluorine substitution on the phenylpyridine ligands.

In line with DFT predictions (Figure 4), the most positive oxidation potential is measured for complex [Ir­(dFppy)_2_(acac)] (**C7**), while the lowest oxidation potential is observed for the fluorine-free [Ir­(ppy)_2_(acac)] (**C6**) analogue, closely followed by complex **C3** (Table 1). It should be also emphasized that the asymmetric complex **C5** shows an oxidation potential that is exactly in between the ones of its symmetrical parent compounds **C1** and **C3** (i.e., +0.529 vs +0.600 and +0.458 V, respectively, see Table 1), demonstrating how the different chelation mode of the 2-(3-fluorophenyl)­pyridine is able to exert a markedly different averaged ligand field around the central iridium­(III) ion, due to the strong delocalization of the HOMO on both the iridium *d* orbitals and phenyl moieties of the cyclometalating ligands (Figure 4).

As far as the cathodic region is concerned, the variation of the first reduction potentials across the series is substantially lower (about 50%) compared to that observed for oxidation potentials (i.e., 0.052 vs 0.103 V, respectively, see Table 1). This is because the different position of the fluorine group on the phenyl ring of the cyclometalating ligands directly affects the HOMO, but only indirectly influences the electron density on the pyridine subunit, where the LUMO is located.

The experimentally determined redox gaps span a range from 2.910 to 3.185 V, corresponding to complexes **C3** and **C7**, respectively. Accordingly, complex **C3** is expected to show the most red-shifted absorption (and emission) spectrum of the whole series, followed by the fluorine-free compound **C6**, and the asymmetric complex **C5**. On the contrary, always based on redox gaps, complex **C7** (equipped with bis-fluorinated cyclometalating ligands) is expected to display the bluest emission, followed by the monofluorinated complexes [Ir­(oFppy)_2_(acac)] (**C1**) and [Ir­(m_A_Fppy)_2_(acac)] (**C2**).

### Photophysical Properties and Excited-State Calculations

The UV–vis absorption spectra of complexes **C1**–**C7** were recorded in acetonitrile solutions at 298 K (Figure 6).

**Figure 6:**
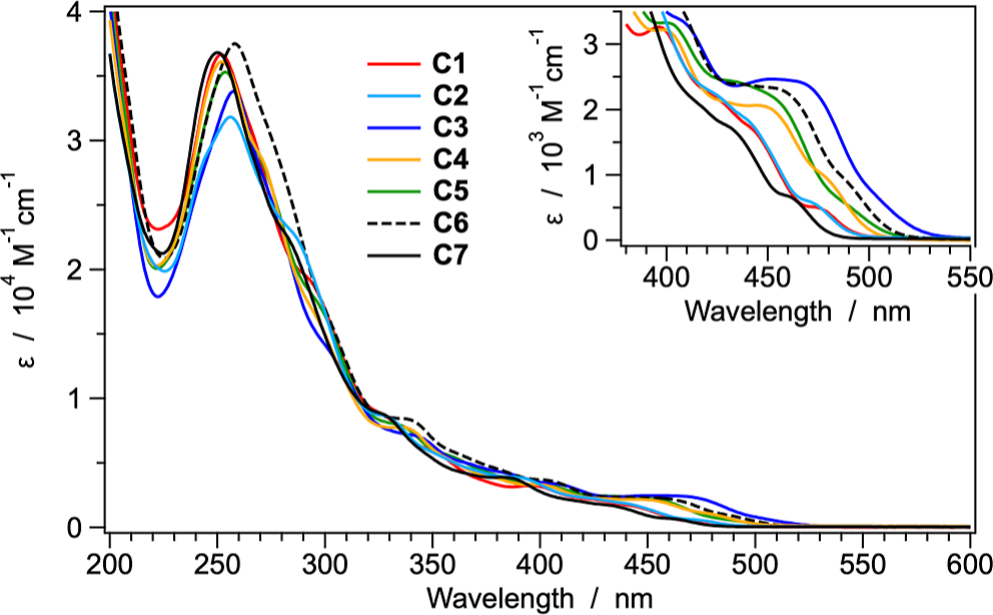
Absorption spectra of complexes **C1–C7** in acetonitrile solution at 298 K. Lowest-energy transitions are magnified in the inset.

As commonly found in cyclometalated iridium­(III) complexes, the intense absorption bands observed in the 200–300 nm range are associated with spin-allowed π–π* ligand-centered (LC) transitions involving both the cyclometalating ligands and the acetylacetonate ancillary one.^8,9,15^ Indeed, all the investigated complexes show similar features in this region, with absorption maxima ranging between 250 and 260 nm, with similar intensities (ε ≈ (3.4 ± 0.3)·10^4^ M^–1^ cm^–1^, see Figure 6). In the longer wavelength region (300–400 nm), the weaker and broader absorption features are attributed to charge–transfer transitions, which may possess ligand-to-ligand (LLCT), intraligand (ILCT), or metal-to-ligand (MLCT) character.

The inset of Figure 6 highlights the lowest-energy absorption bands of all the complexes. The most red-shifted features (λ > 450 nm) are attributed to the direct population of the lowest triplet state via the formally spin-forbidden S_0_ → T_1_ transition. Although these transitions are partially allowed due to the strong spin–orbit coupling of the iridium center,^8^ they are extremely weak (ε < 1000 M^–1^ cm^–1^) and can be clearly detected only for **C1**, **C2** and **C7**, while they appear as shoulders for the other complexes. Notably, the energy of the S_0_ → T_1_ absorption band increases in the following order: **C3** < **C6** < **C5** < **C4** < **C1** ≈ **C2** < **C7**. This trend closely mirrors the redox gaps determined from electrochemical measurements (see previous section), confirming the strong correlation between optical and electrochemical properties and suggesting that the T_1_ is directly related to the HOMO → LUMO excitation.

To gain a more detailed understanding of the excited states, the lowest-lying triplets of all complexes (**C1**–**C7**) were examined using time-dependent DFT (TD-DFT) calculations. The lowest triplet transitions are summarized in Tables S1–S7, each described in terms of their dominant natural transition orbital (NTO) pairs.^50^ For a more intuitive picture, Figure 7 provides a concise visualization of the triplet-state energy landscape at the Franck–Condon region for the entire series of investigated complexes.

**Figure 7:**
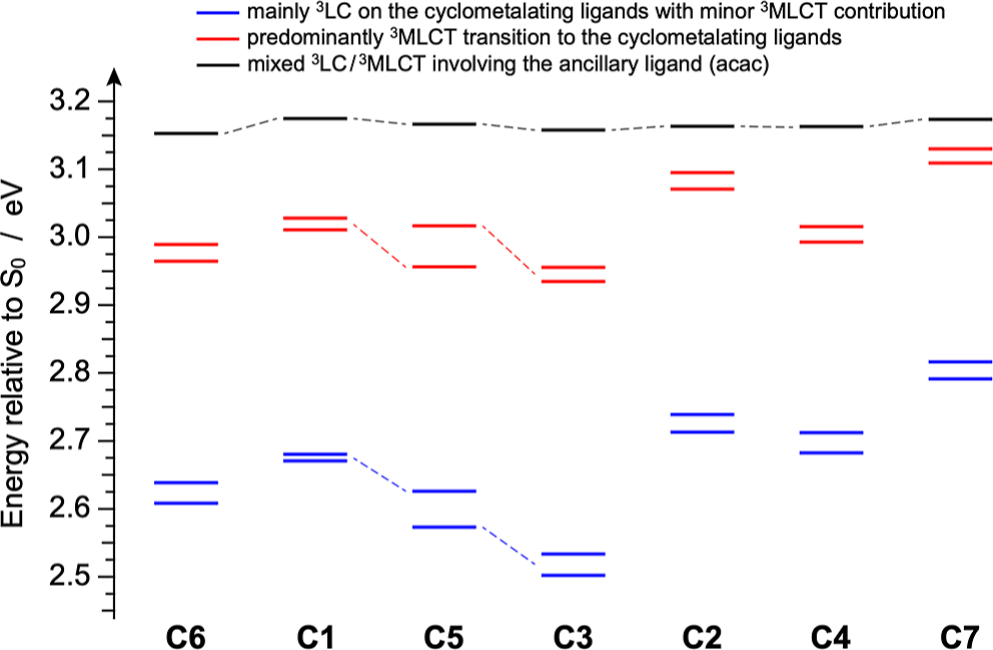
Energy diagram of the lowest triplet states for complexes **C1**–**C7**, calculated in acetonitrile as vertical excitations from their optimized ground-state geometries.

TD-DFT calculations further support the assignment of the lowest-energy absorption band observed in the 450–550 nm range for complexes **C1**–**C7** (Figure 6, inset) to the S_0_ → T_1_ transition, having a predominant HOMO → LUMO character and a ^3^LC nature (Tables S1–S7). As illustrated in Figure 7, the computed S_0_ → T_1_ transition energies are in good agreement with those inferred by absorption, as discussed above.

In all complexes, except for the asymmetric complex **C5**, the T_2_ state is nearly degenerate with T_1_, a consequence of their *C*_2_ symmetry leading to equivalent cyclometalating ligands. Conversely, in the case of **C5**, the energy separation between T_1_ and T_2_ increases to 0.06 eV, since each triplet is associated with an excitation localized on one of the two distinct cyclometalating ligands (Table S5). For all the compounds, a second pair of triplet states (i.e., T_3_ and T_4_) is predicted at approximately (0.36 ± 0.4) eV above the lowest nearly degenerate triplet pair. These higher-lying triplets exhibit a stronger ^3^MLCT character, while still involving the cyclometalating ligands. Only the T_5_ state starts to involve the acetylacetonate ancillary ligand, which is identical across all complexes; as a result, the energy of the S_0_ → T_5_ transition is consistently estimated at (3.16 ± 0.01) eV for the entire series (Figure 7 and Tables S1–S7).

The normalized emission spectra of complexes **C1**–**C7**, recorded in acetonitrile at 298 K, are presented in the top panel of Figure 8; for comparative purposes, the same measurements carried out at 77 K in butyronitrile glass are shown in the bottom panel. The corresponding emission properties and key photophysical parameters are summarized in Table 2 for each complex.

**Figure 8:**
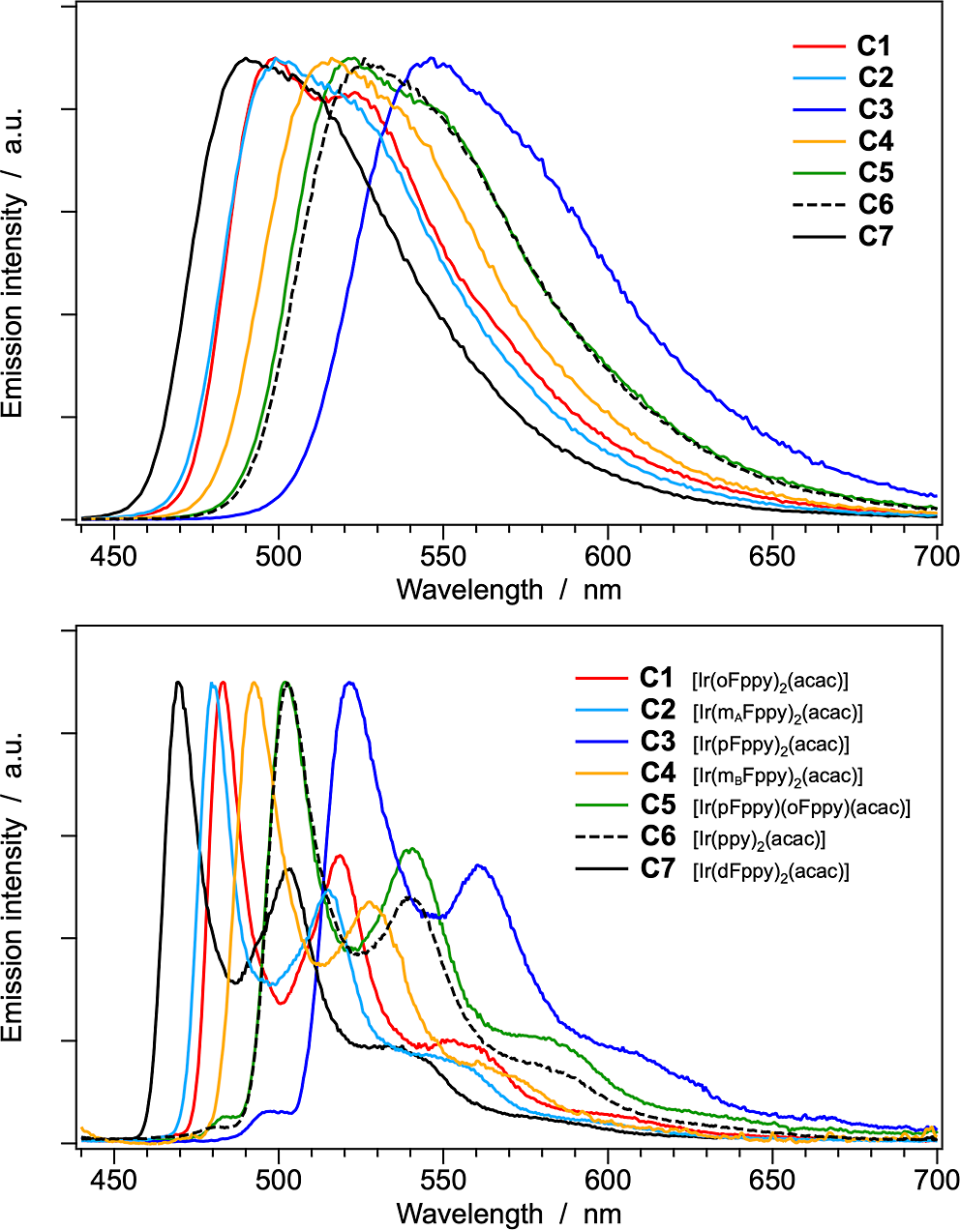
Normalized emission spectra of complexes **C1–C7** in acetonitrile at 298 K (top) and in butyronitrile glass at 77 K (bottom). Sample concentration: ≈20 μM.

**Table 2:** Luminescence Properties and Photophysical Parameters of Complexes **C1**–**C7** in Different Media

	CH_3_CN oxygen-free solution, 298 K					BuCN rigid matrix, 77 K		1% PMMA matrix, 298 K				
	λ_em_ ^a^ ^b^ [nm]	PLQY^a^ [%]	τ^b^ [μs]	*k*_r_ ^c^ [10^5^ s^–1^]	*k*_nr_ ^d^ ^e^ [10^5^ s^–1^]	λ_em_ ^a^ [nm]	τ^e^ [μs]	λ_em_ ^a^ [nm]	PLQY^f^ [%]	τ^b^ [μs]	*k*_r_ ^c^ [10^5^ s^–1^]	*k*_nr_ ^d^ [10^5^ s^–1^]
**C1**	498, 522	53.5	1.43	3.73	3.25	483, 519, 550	4.94	495, 524	58.2	1.59	3.67	2.64
C2	501, 520^sh^	55.5	1.20	4.65	3.72	480, 515, 547^sh^	3.75	493, 518	56.9	1.29	4.41	3.34
C3	546, 570^sh^	54.0	1.87	2.89	2.46	522, 561, 605^sh^	6.14	544, 562^sh^	57.4	2.22	2.59	1.92
C4	515, 530^sh^	70.5	1.48	4.77	2.00	493, 528, 562^sh^	5.82	508, 530	57.5	1.26	4.58	3.39
C5	522, 542^sh^	60.8	1.88	3.23	2.09	502, 540, 577^sh^	5.53	518, 541	59.5	1.82	3.28	2.23
C6	526, 540^sh^	58.9	1.61	3.66	2.55	503, 540, 580^sh^	5.60	518, 543^sh^	52.5	1.28	4.11	3.72
**C7**	490, 505^sh^	52.0	0.975	5.34	4.92	470, 503, 532^sh^	3.61	481, 507	69.7	1.17	5.94	2.58

a λ_exc_ = 430 nm.

b λ_exc_ = 465 nm.

c Radiative constant: *k*_r_ = PLQY/τ.

d Nonradiative constant: *k*_nr_ = 1/τ – *k*_r_.

e λ_exc_ = 370 nm.

f Photoluminescence quantum yield determined by integrating sphere; λ_exc_ = 430 nm.

It is particularly noteworthy that, at both 298 and 77 K, the most red-shifted emission in the entire series is observed for complex **C3**, rather than for the archetypal fluorine-free reference complex [Ir­(ppy)_2_(acac)] (**C6**); this result challenges the commonly held assumption that fluorinated phenylpyridine ligands invariably induce a hypsochromic shift in the emission of the related cyclometalated iridium­(III) complexes, if compared to their nonfluorinated analogues. Even more surprisingly, the emission spectrum of the asymmetric complex **C5** is virtually identical to that of the prototypical **C6**, despite incorporating two distinct monofluorinated cyclometalating ligands (Figure 8). In contrast, and more in line with conventional expectations, the emission maxima of complexes **C1**, **C2**, and **C4** (each bearing a monofluorinated phenylpyridine derivative) are found between those of the well-known poly fluorinated and blue-emitting [Ir­(dFppy)_2_(acac)] complex (**C7**) and fluorine-free [Ir­(ppy)_2_(acac)] counterpart (**C6**), reflecting a somehow predictable intermediate degree of electronic perturbation exerted by the fluorine substituents (Table 2 and Figure 8).

As suggested by TD-DFT calculations, all complexes emit from predominantly ^3^LC states localized on the variously fluorinated phenylpyridine cyclometalating ligands. This assignment is supported by the following experimental evidence: (i) the absence of significant spectral shifts upon cooling from 298 to 77 K, with the emission onset remaining unchanged at both temperatures; (ii) the presence of weak vibronic features in the room-temperature spectra, which become markedly more pronounced in the frozen glass at 77 K (Figure 8, top and bottom panels); and (iii) the observation that all the 77 K spectra display identical vibronic progressions, suggesting that the effective vibrational mode coupling the T_1_ and S_0_ states has always a frequency of approximately (1275 ± 50) cm^–1^, which is typical of the phenylpyridine in-plane ring stretching modes (Figure S41 and Table S8).

The ^3^LC nature of the emissive states is further confirmed by spin-unrestricted DFT calculations (Figure S42), which reveal that the spin-density in the lowest-energy, fully relaxed triplet states is always predominantly localized on the cyclometalating ligands, with only minor contributions from the iridium *d* orbitals. Notably, in the case of the asymmetric complex **C5**, which features two different cyclometalating ligands, two close lying triplets are found (Δ*E* = 63 meV), each centered on one of the two different Fppy ligands. As expected, the lower-energy triplet is localized on the ligand that cyclometalates in the same fashion as in **C3**, the most red-emissive complex among the series (Figure S42).

All complexes are bright emitters in oxygen-free acetonitrile solutions at 298 K, with photoluminescence quantum yields (PLQYs) ranging from 52 to 71%; notably, the highest PLQY value is reached in complex **C4**, which displays the lowest nonradiative rate constant of the series (Table 2). As commonly observed in cyclometalated iridium­(III) complexes, the excited state lifetimes of all the complexes are substantially longer at 77 K, due to the different thermal equilibration (and populations) of the triplet sublevels.^62,63^

The photophysical characterization of the complexes was also carried out in solid state by embedding the complexes in a poly­(methyl methacrylate) (PMMA) matrix at a concentration of 1% by weight. The corresponding emission spectra, recorded at 298 K under ambient conditions, are shown in Figure 9, while the associated photophysical data are summarized alongside the solution data in Table 2.

**Figure 9:**
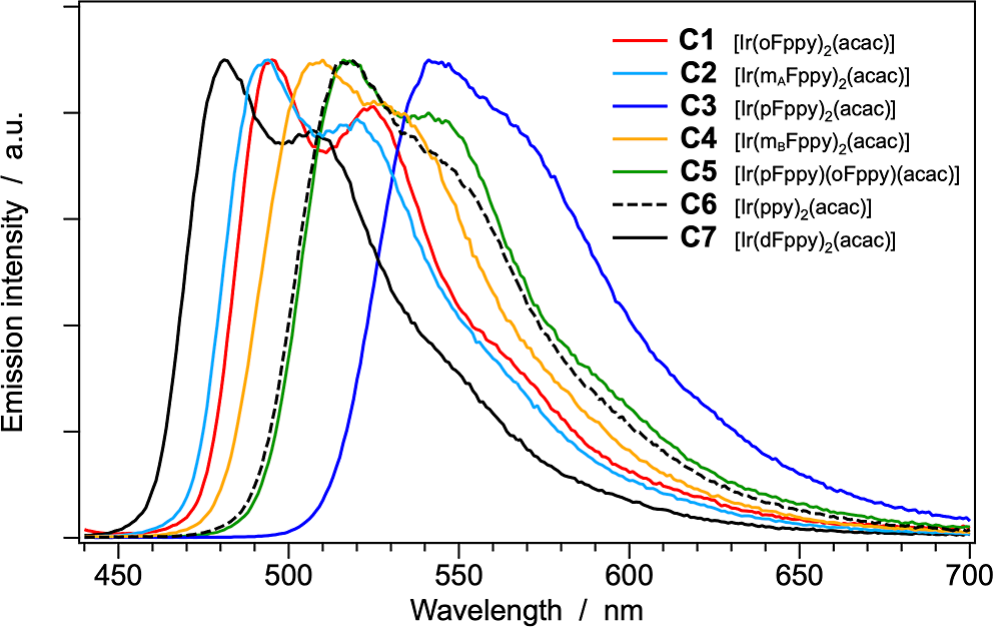
Normalized emission spectra of complexes **C1–C7** in 1% w./w. PMMA matrix at 298 K.

In diluted PMMA matrix, the photophysical performances of all the complexes remain very similar to those already measured in acetonitrile solutions at 298 K (e.g., similar PLQYs and lifetimes) and only a systematic minor blue-shift of the emission profiles is observed in the polymeric film, which is a further indication of the ^3^LC nature of all the emissive states.

### Rationalization of ppy Fluorination Effects

The full characterization and the comparative analysis of complexes **C1**–**C7** allows to extract general guidelines on how the position of fluoro substituents within phenylpyridine ligands dictates the electronic structure and photophysical response of the related cyclometalated iridium­(III) complexes.

Fluorine, as a substituent, exhibits both a strong electron-withdrawing inductive effect (−I), due to its high electronegativity, and a positive mesomeric effect (+M) or π-donating effect, which involves donating electron density via *p*-orbital overlap with π systems.^64,65^ The inductive effect is stronger in *ortho*, effectively draining the electron density from that position, and gradually decreases as the distance increases in *meta* and *para*. On the contrary, the mesomeric effect is more pronounced in *ortho* and *para*, increasing the electron density in those positions.^66^ It is worth noting that, according to our nomenclature, the *ortho*, *meta* and *para* positions are occupied by the iridium­(III) ion in the present complexes (Figure 10).

**Figure 10:**
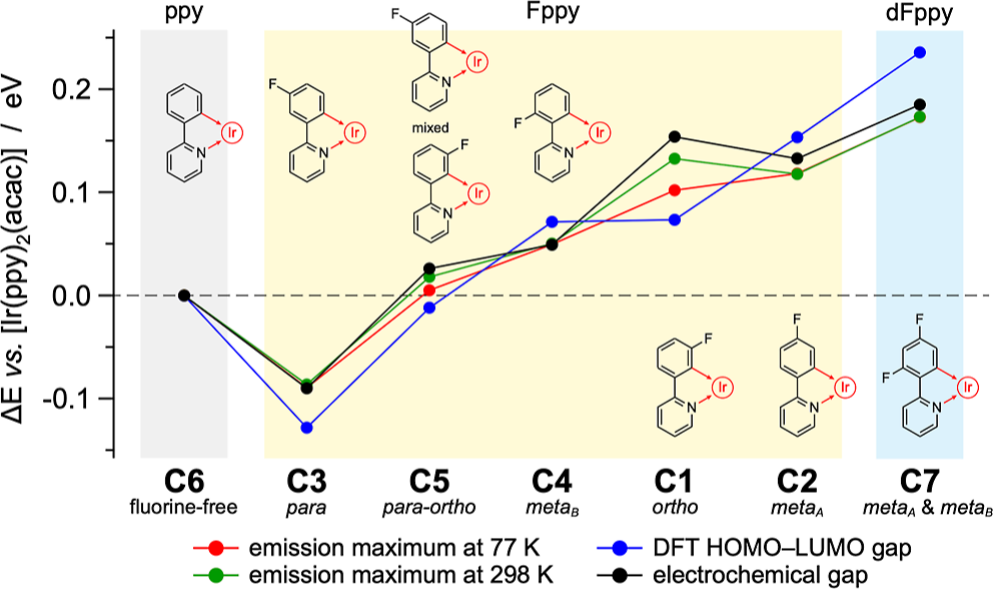
Effects of the fluorination of the phenylpyridine cyclometalating ligands on the photophysical, electrochemical and DFT-computed properties of the related iridium­(III) complexes, compared to the fluorine-free reference [Ir­(ppy)_2_(acac)] (**C6**).

Fluorination in the *ortho* position (as in **C1**) exerts the strongest inductive (−I) effect, which is partially counterbalanced by an important mesomeric (+M) π-donation. This interplay results in only moderate HOMO stabilization but produces an overall widening of both the HOMO–LUMO and the electrochemical gap, leading to a blue-shifted emission relative to **C6**. Because these opposing −I and +M contributions act with different weight on frontier orbitals, redox potentials, and excited-state density, the net outcome is variable (and reflected in the large spreading of the **C1** points in Figure 10), even though the inductive effect remains dominant.

In contrast, *para* substitution is dominated by the +M effect that predominantly lower the LUMO, leaving the HOMO virtually unperturbed. As a result, an unexpected stabilization of the triplet emitting state is observed, and **C3** displays the most red-shifted emission in the series, even compared to **C6**.

In the asymmetric complex **C5** (equipped with one oFppy and one pFppy ligand), the effects already described for **C1** and **C3** are counterbalanced. As a result, **C5** is a fluorinated iridium­(III) complex with the same properties of the fluorine-free [Ir­(ppy)_2_(acac)] reference (**C6**, Figure 10), highlighting the additive yet inherently nonlinear character of substituent effects.

In the case of the *meta* substitutions (meta_A_ and meta_B_, as in **C2** and **C4**, respectively), the predominant effect is the inductive (−I), yielding to a larger Δ*E*, compared to **C6** (Figure 10). Notably, the fluorination in the *meta*_A_ position exerts roughly twice the impact of that in *meta*_B_, underscoring the positional sensitivity of the inductive withdrawing effect. These effects combine in an essentially additive fashion in the well-known bis-fluorinated complex [Ir­(dFppy)_2_(acac)] (**C7**, Figure 10).

While well-defined trends can be identified for emission energies and electrochemical gaps, all investigated complexes exhibit very similar photoluminescence quantum yields (58 ± 6)% and excited-state lifetimes (1.5 ± 0.4) μs, both in room-temperature acetonitrile solution and in a PMMA matrix. As a result, any correlation inferred from these parameters would need to be interpreted with caution, as it may be close to the limits of the experimental uncertainty (see Experimental Section).

Even the ^19^F NMR data were examined in the context of the fluorine substitution pattern; however, no unambiguous correlations with the photophysical properties could be identified. Moreover, any tentative interpretation is further complicated by solvent-dependent effects arising from solubility limitations (not all spectra could be recorded in CDCl_3_).

## Conclusions

In this work, we have conducted a systematic investigation on the effects of positional fluorination in 2-phenylpyridine ligands coordinated to neutral [Ir­(Fppy)_2_(acac)] complexes. Seven distinct emitters, including four positional isomers (**C1**–**C4**), an unprecedented asymmetric derivative (**C5**), and reference complexes [Ir­(ppy)_2_(acac)] (**C6**) and [Ir­(dFppy)_2_(acac)] (**C7**) were synthesized and fully characterized through electrochemical methods, steady-state and time-resolved spectroscopic techniques (both in solution and in solid state), and their properties were rationalized with the support of DFT and TD-DFT calculations.

Our results show that fluorination does not exert a mere hypsochromic effect as the number of fluorine substituents increases, but rather its impact depends mainly on the substitution position. *Meta* substitutions primarily stabilize the HOMO via inductive effects, while *para* substitution lowers the LUMO through resonance interactions (mesomeric effect), unexpectedly leading to the most red-shifted emission of the series. This overturns the conventional assumption that fluorination always drives the emission of the corresponding iridium­(III) complexes to higher energies. The asymmetric derivative **C5** further highlights how mixed cyclometalation modes yield intermediate properties compared to the symmetric parent compounds (**C1** and **C3**).

From these findings, clear design principles emerge: (i) fluorination acts as a position-sensitive rather than just a number-sensitive tuning handle; (ii) inductive and mesomeric contributions can be selectively combined to modulate HOMO and LUMO levels, redox potentials, and emission energies; and (iii) *para* substitution offers an unconventional route to obtain redder emission than fluorine-free equivalents. These principles can be easily extended beyond the present series and can guide the rational engineering of other cyclometalated iridium­(III) complexes with innocent ancillary ligands for next-generation OLEDs, photocatalysts, and photonic devices. The strategy can be more broadly extended to luminescent complexes of other transition metals.

## Supplementary Material



## Notes

Electronic Supporting Information NMR spectra of ligands and complexes, cyclic electrochemical data, TD-DFT excitations, vibronic analysis of emission spectra, and triplet spin densities (PDF)
