# Supplementary material for: Positional Fluorination of Phenylpyridine: Unexpected Electronic Tuning in Bis-Cyclometalated Iridium(III) Acetylacetonate Complexes
Source: Inorg Chem. 2025 Dec 29;65(1):821–33. doi: 10.1021/acs.inorgchem.5c05152 (PMC12801313; doi:10.1021/acs.inorgchem.5c05152)
Supplement: Supplementary file 1 [file ic5c05152_si_001.pdf]

Supporting Information

Positional Fluorination of Phenylpyridine: Unexpected Electronic Tuning in Bis-cyclometalated Iridium(III) Acetylacetonate Complexes

Silvia Sigismondi,[†] Valentina Montani,[§] Morgan Gaggioli,[§] Daniele Tedesco,[†] Nicola Armaroli,[†] Letizia Sambri,[§] Filippo Monti,*[†] and Andrea Baschieri*[†]

† Institute for Organic Synthesis and Photoreactivity (ISOF), National Research Council of Italy (CNR), Via Piero Gobetti 101, 40129 Bologna, Italy.

§ Department of Industrial Chemistry "Toso Montanari", University of Bologna, Via Piero Gobetti 85, 40129 Bologna, Italy.

* E-mail: filippo.monti@isof.cnr.it (F.M.), andrea.baschieri@isof.cnr.it (A.B.)-

Table of Contents

Contents	*Pages*

NMR spectra of ligands **L1**, **L2** and **L3**	S2 – S3
NMR spectra of complexes **C1**–**C7**	S4 – S20
Ratio of **C1**, **C3**, and **C5** isomers in the reaction crudes, determined by ^{1}H NMR.	S21
NMR spectra of CH signals of pure complexes **C1**, **C3** and **C5**.	S22
Ratio of **C1**, **C3**, and **C5** isomers in the reaction crudes, determined by analytical HPLC.	S22
Electrochemical data	S23
Excited-state computational and photophysical data	S24 – S32

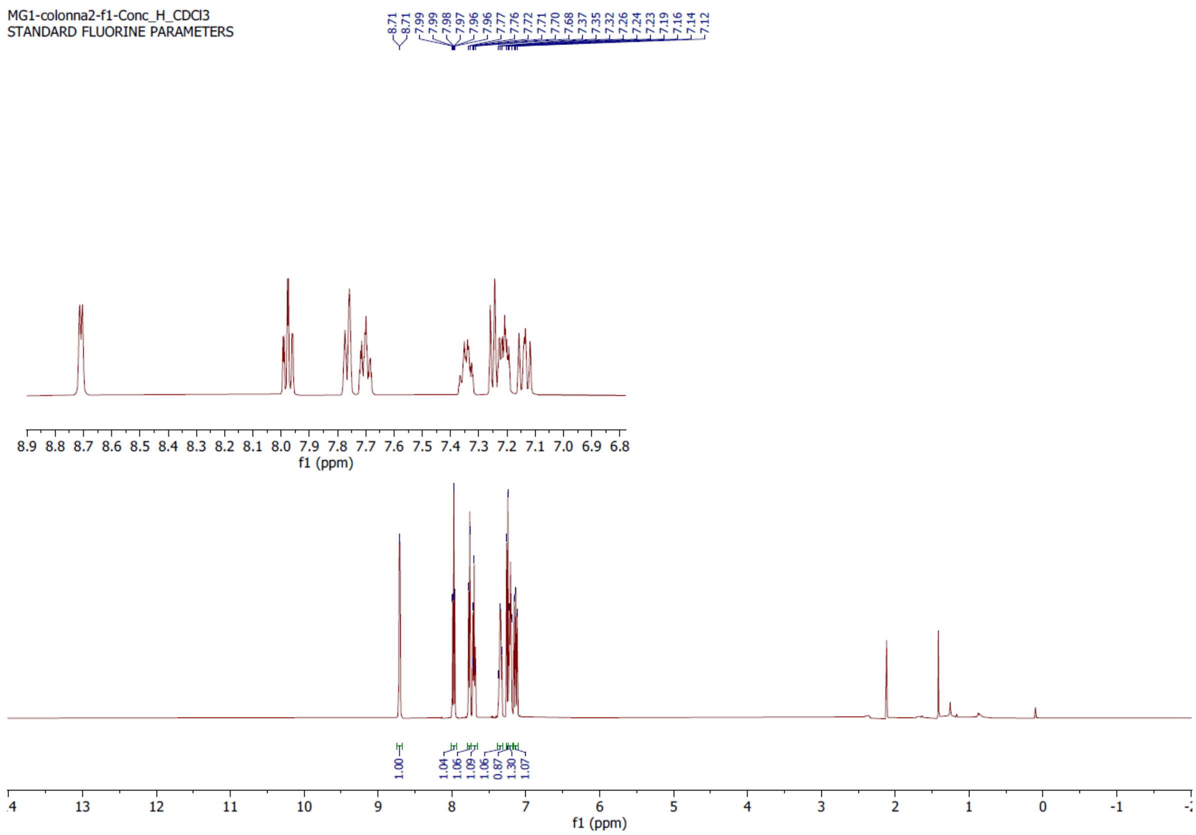

Figure S1. ¹H NMR spectrum of ligand **L1**.

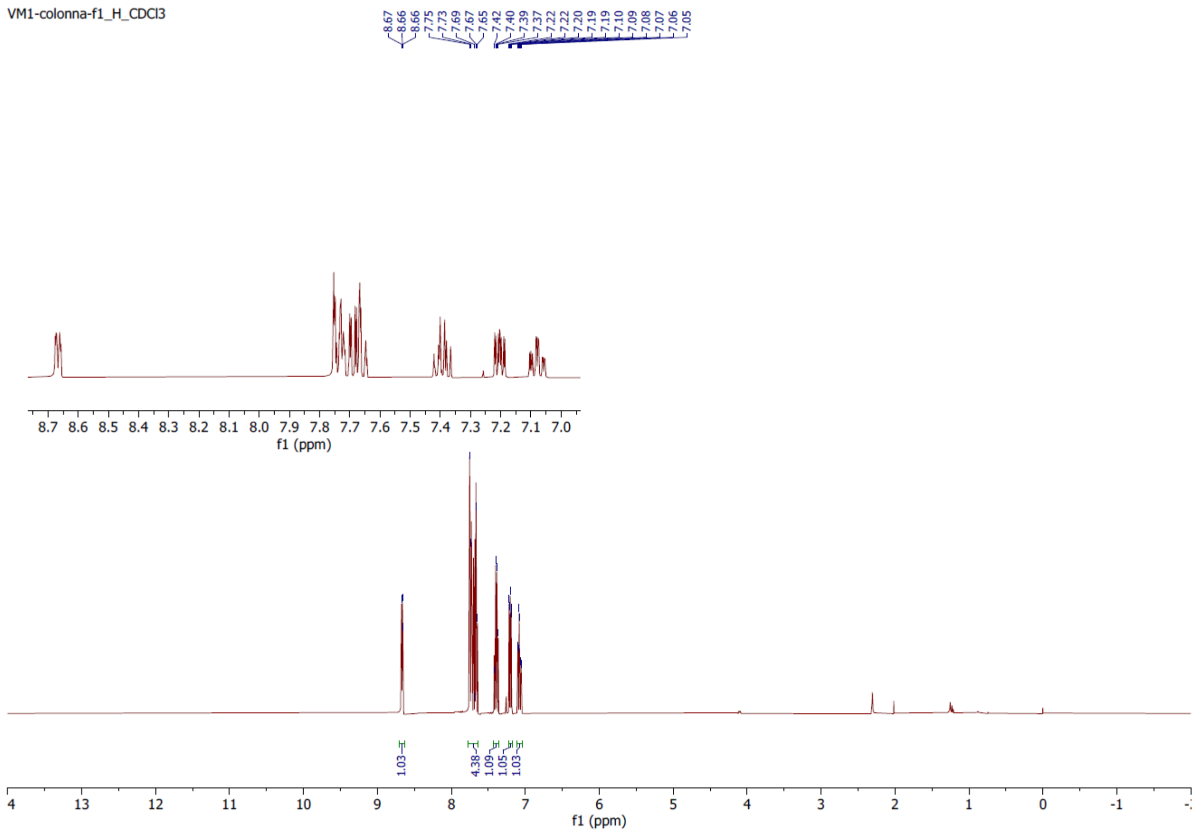

Figure S2. ¹H NMR spectrum of ligand **L2**.

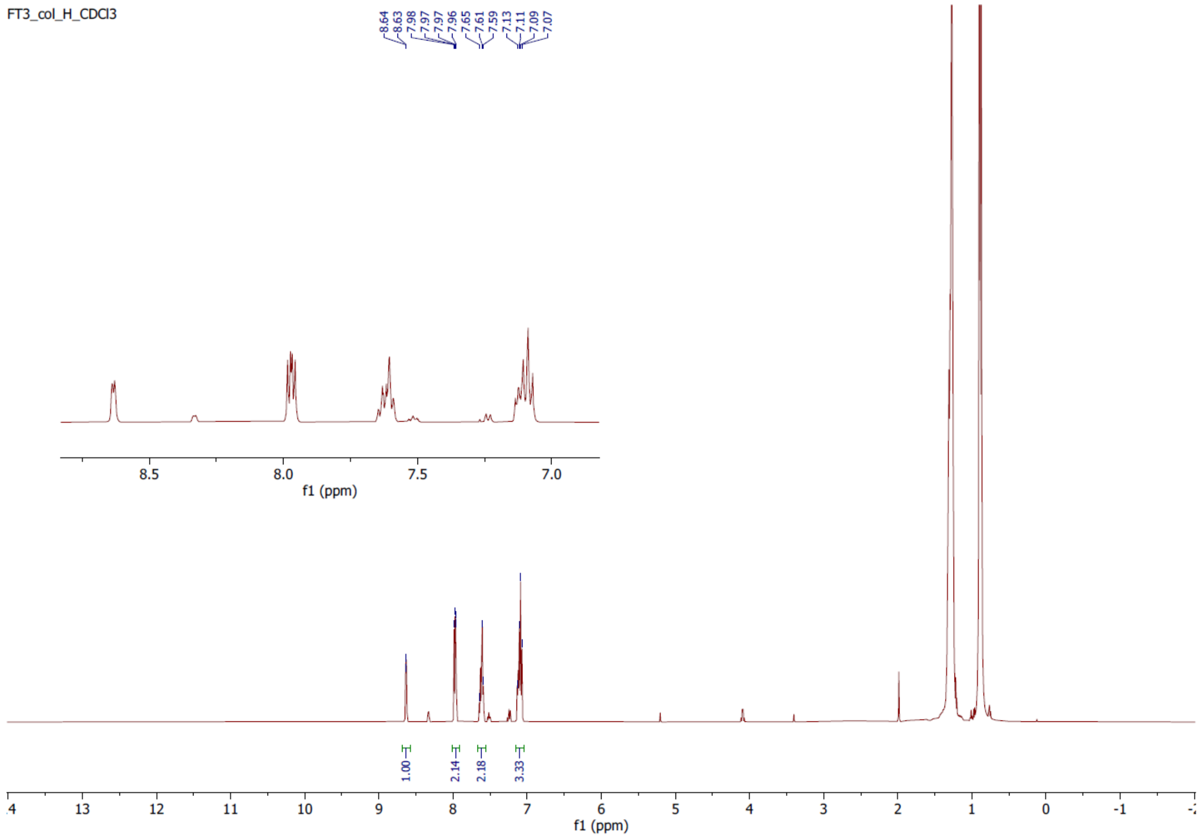

Figure S3. ^{1}H NMR spectrum of ligand **L3**.

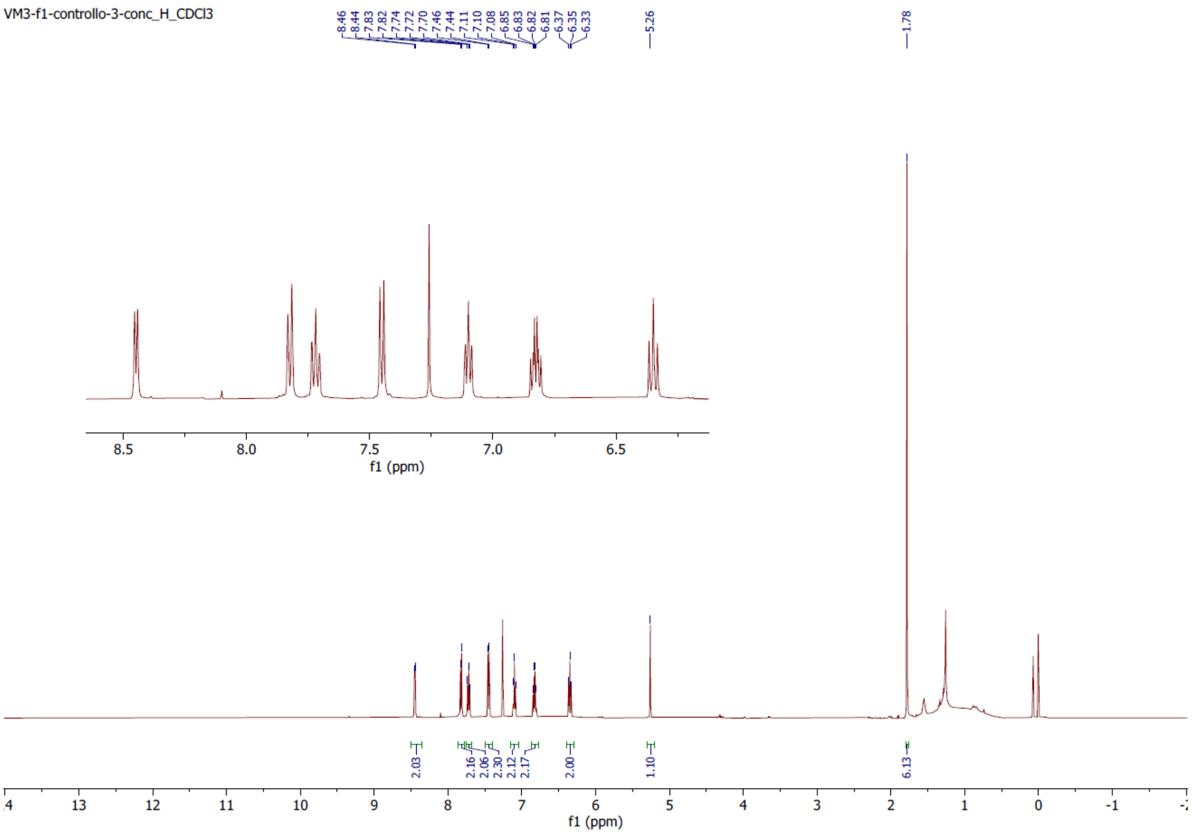

Figure S4. ^{1}H NMR spectrum of complex **C1**.

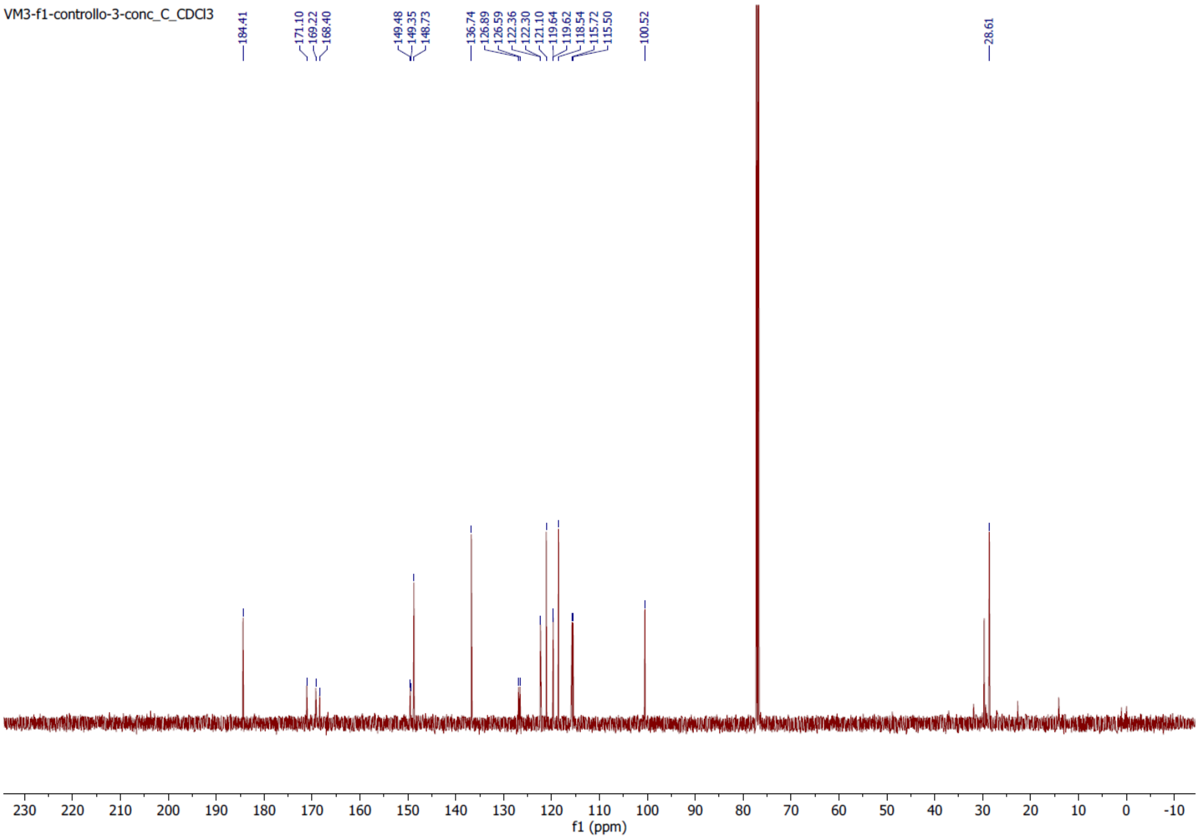

Figure S5. ^{13}C NMR spectrum of complex **C1**.

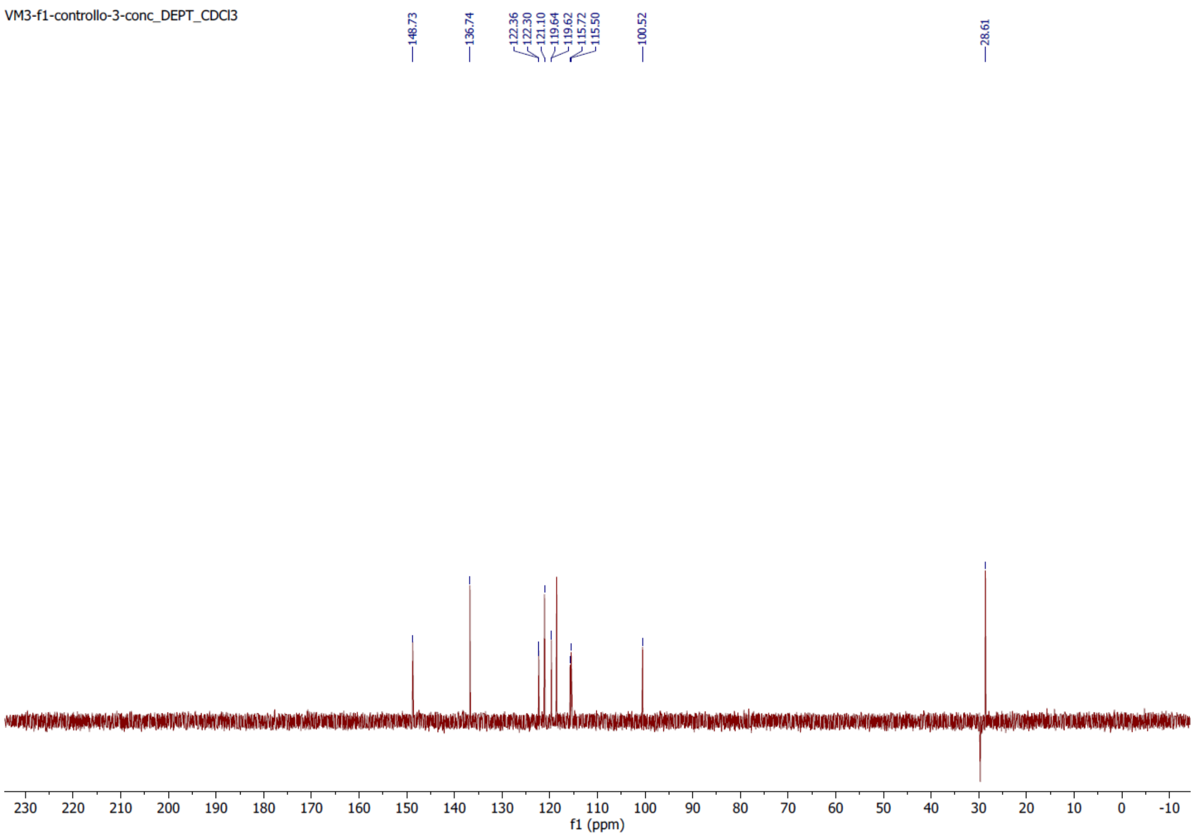

Figure S6. DEPT 135 NMR spectrum of complex **C1**.

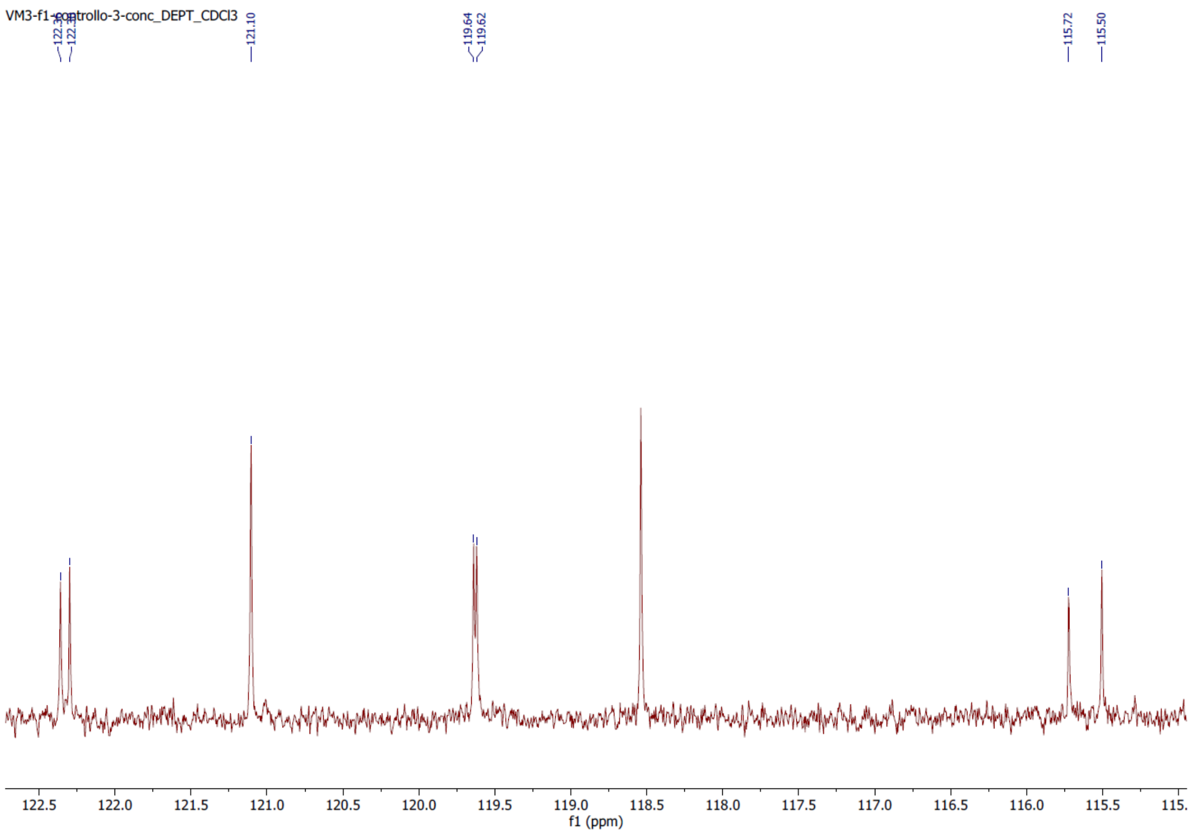

Figure S7. DEPT 135 NMR spectrum of complex **C1** with zoom on J(C_H-F).

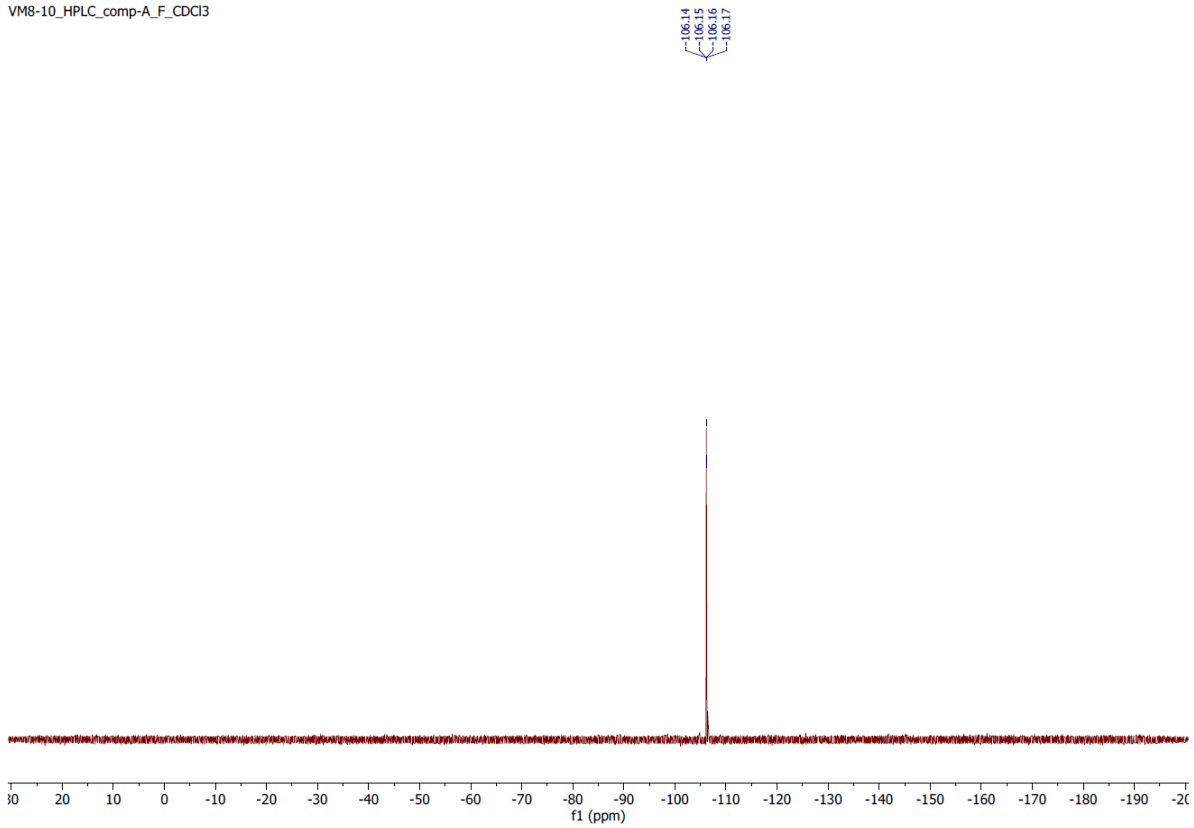

Figure S8. ^{19}F NMR spectrum of complex **C1**.

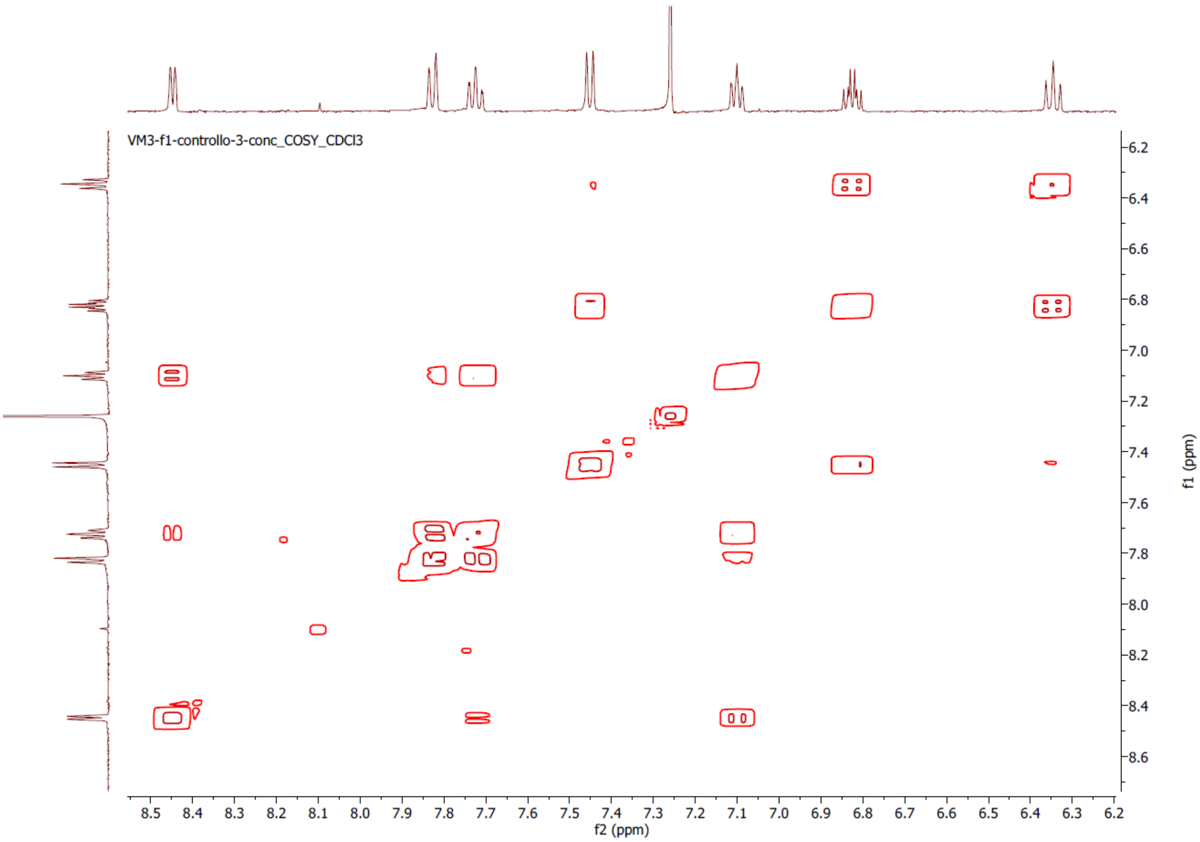

Figure S9. COSY NMR spectrum of complex **C1**.

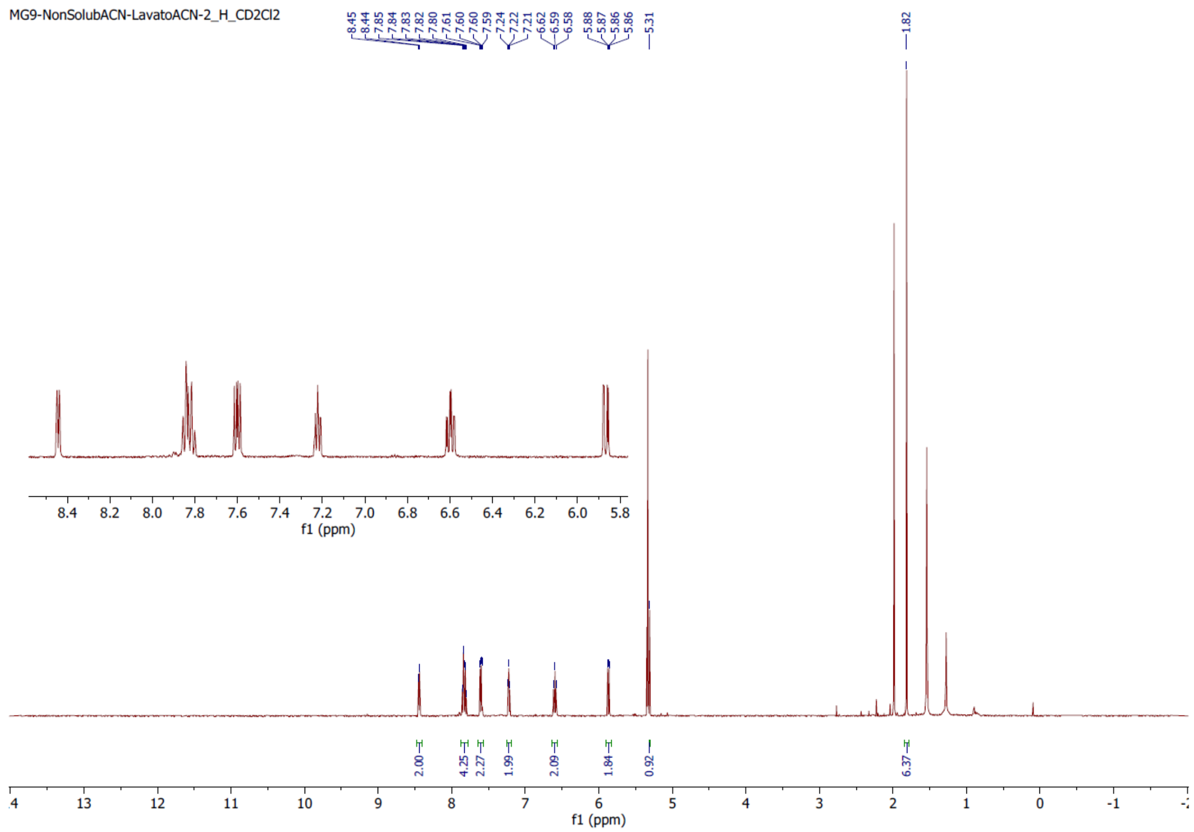

Figure S10. ^{1}H NMR spectrum of complex **C2**.

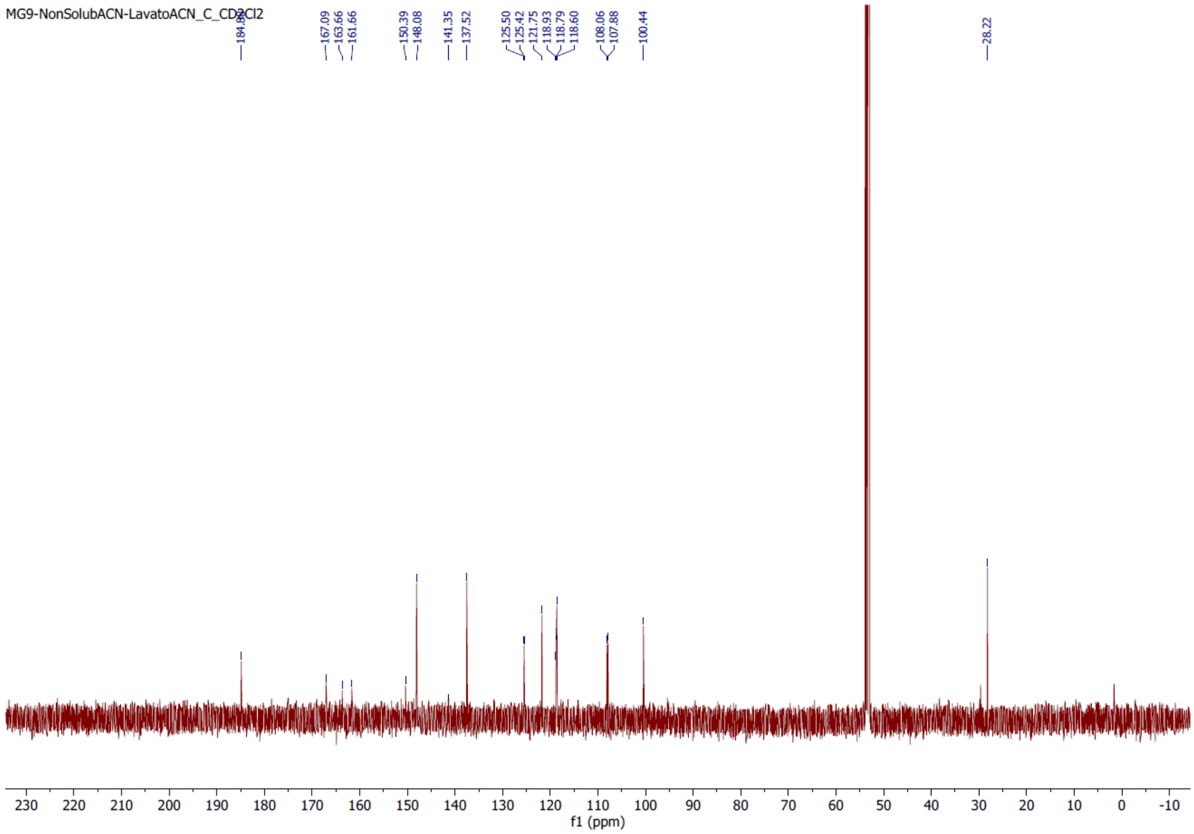

Figure S11. ^{13}C NMR spectrum of complex **C2**.

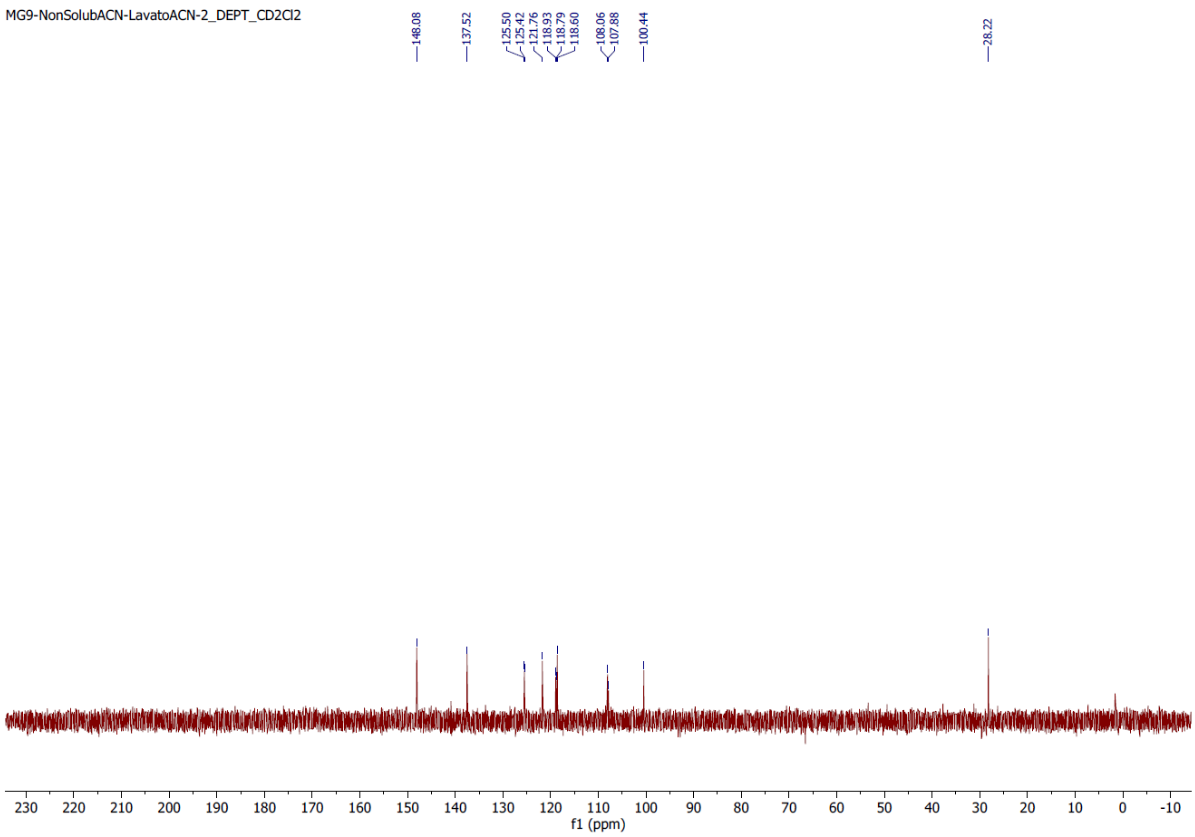

Figure S12. DEPT 135 NMR spectrum of complex **C2**.

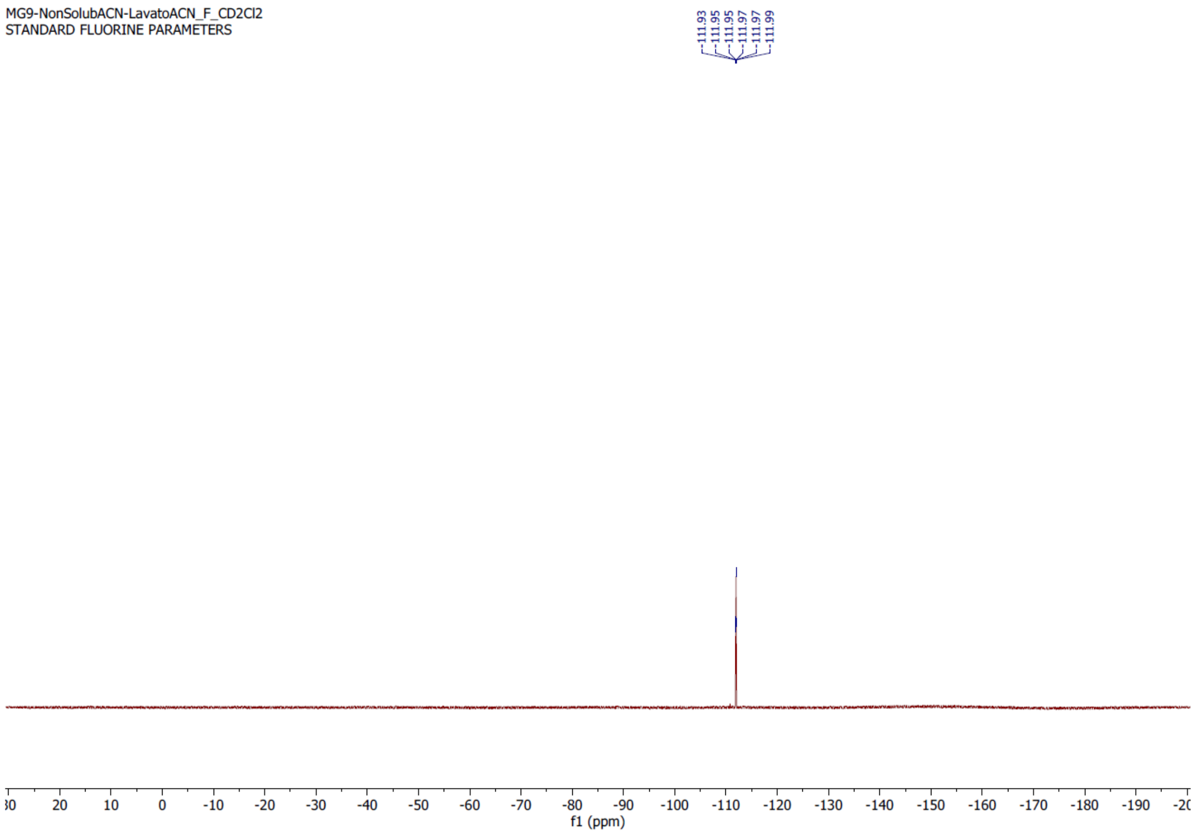

Figure S13. ^{19}F NMR spectrum of complex **C2**.

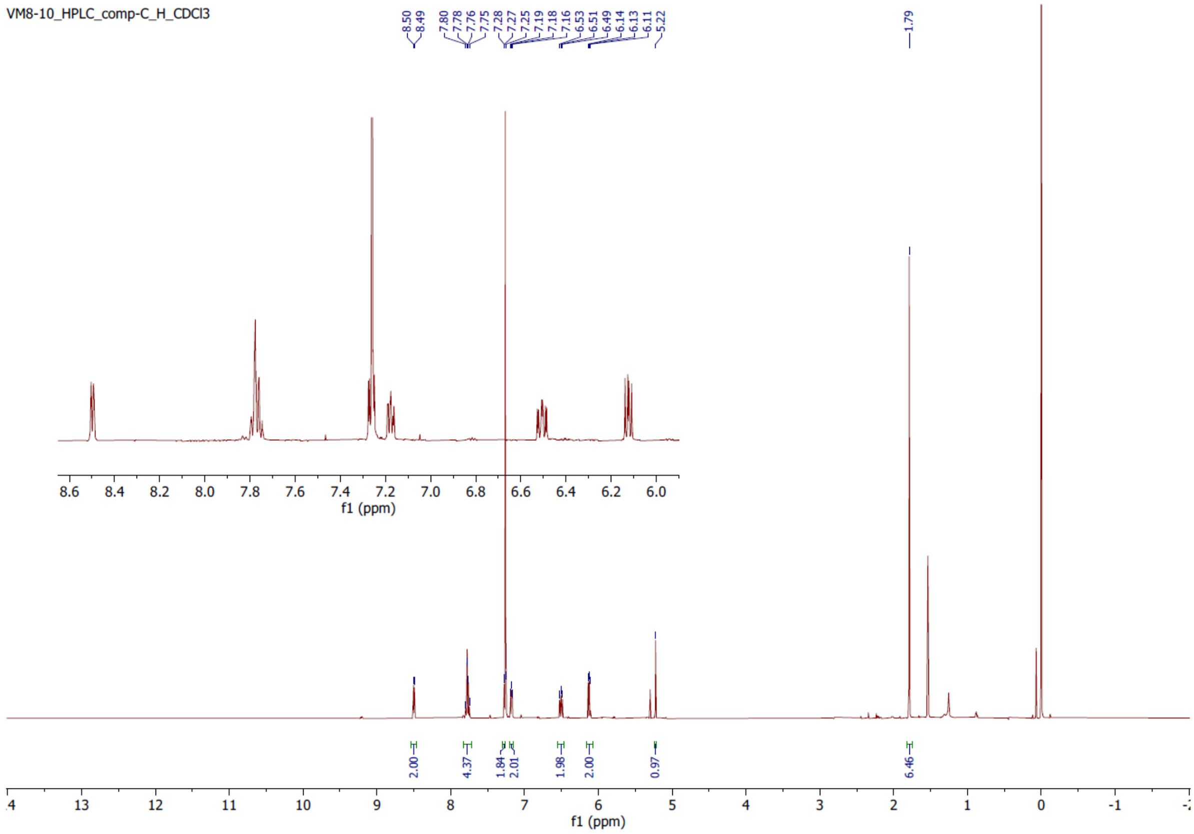

Figure S14. ^{1}H NMR spectrum of complex **C3**.

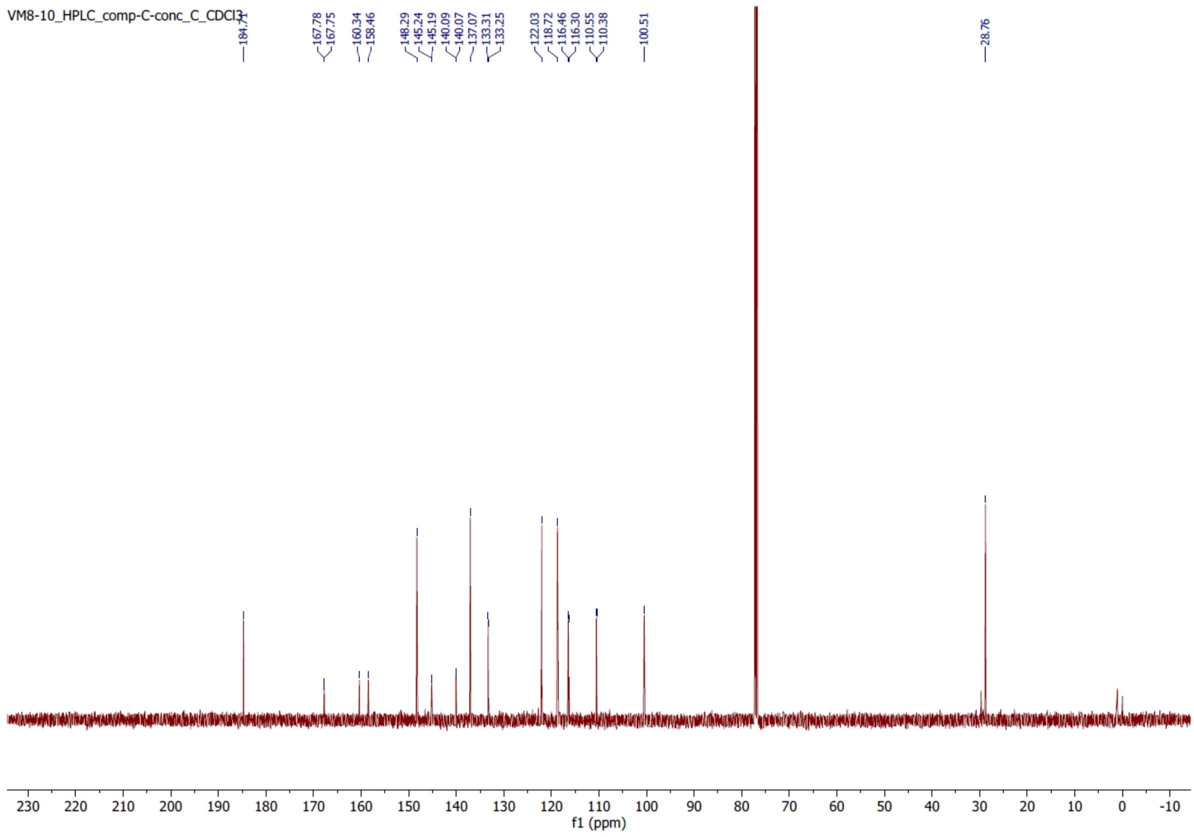

Figure S15. ^{13}C NMR spectrum of complex **C3**.

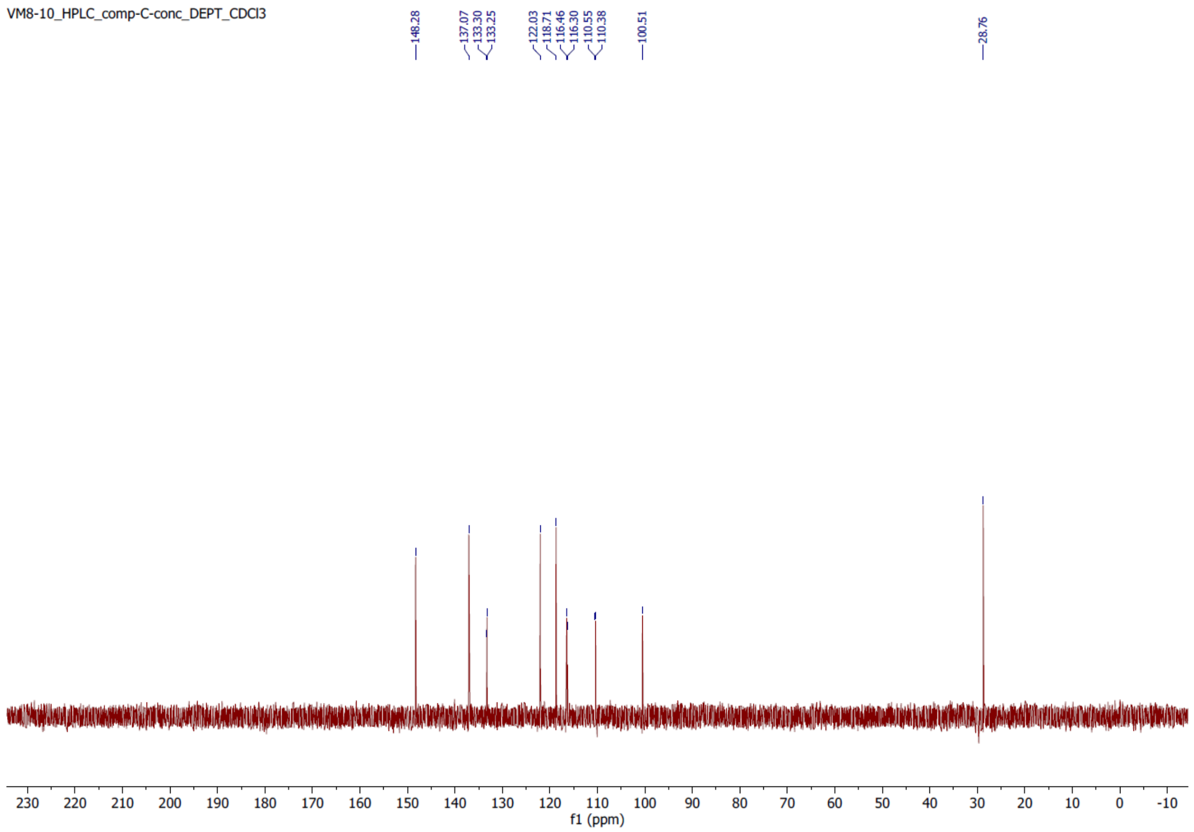

Figure S16. DEPT 135 NMR spectrum of complex **C3**.

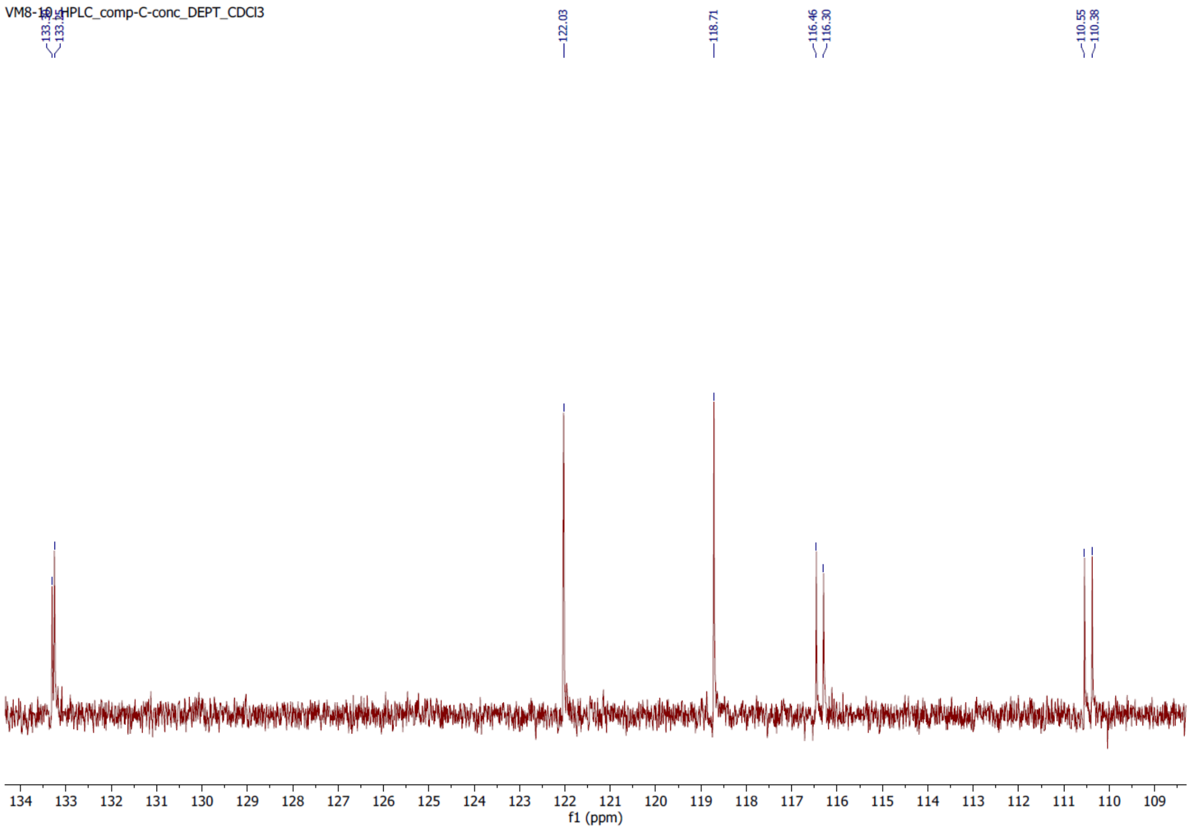

Figure S17. DEPT 135 NMR spectrum of complex **C3** with zoom on J(C_H-F).

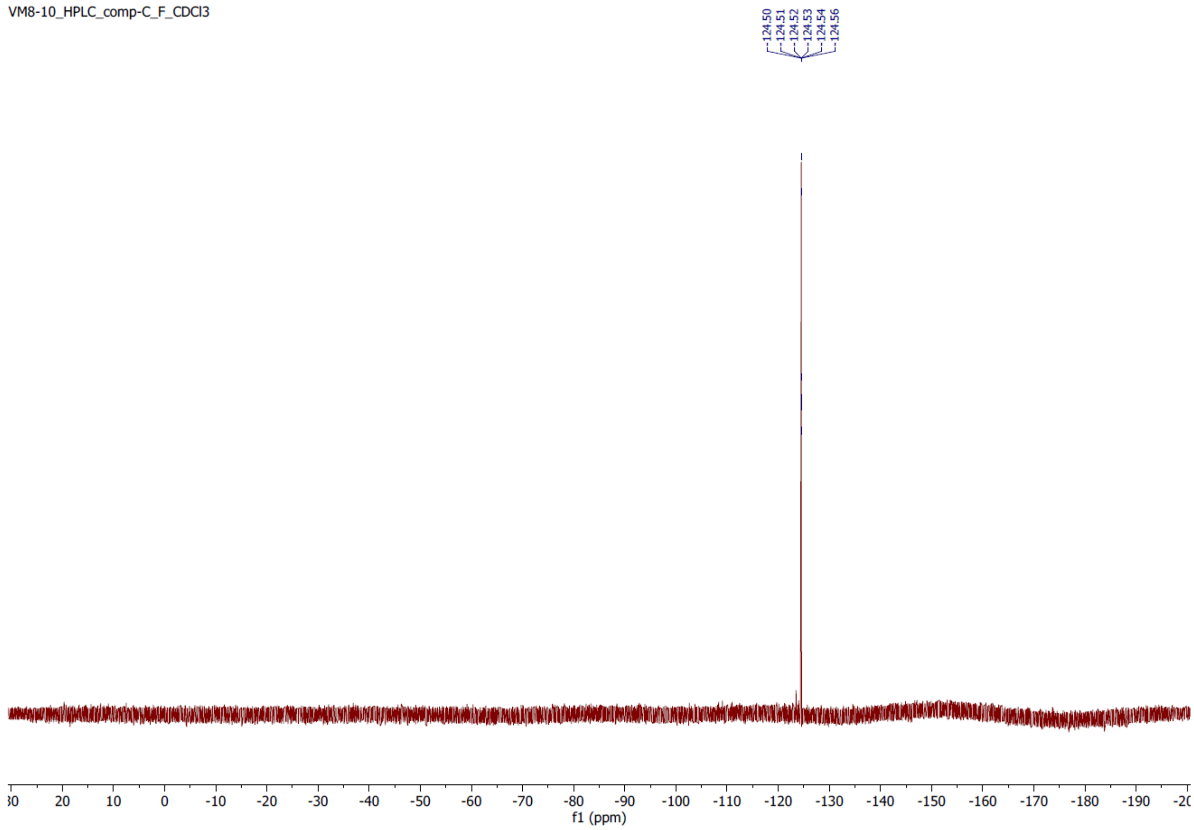

Figure S18. ^{19}F NMR spectrum of complex **C3**.

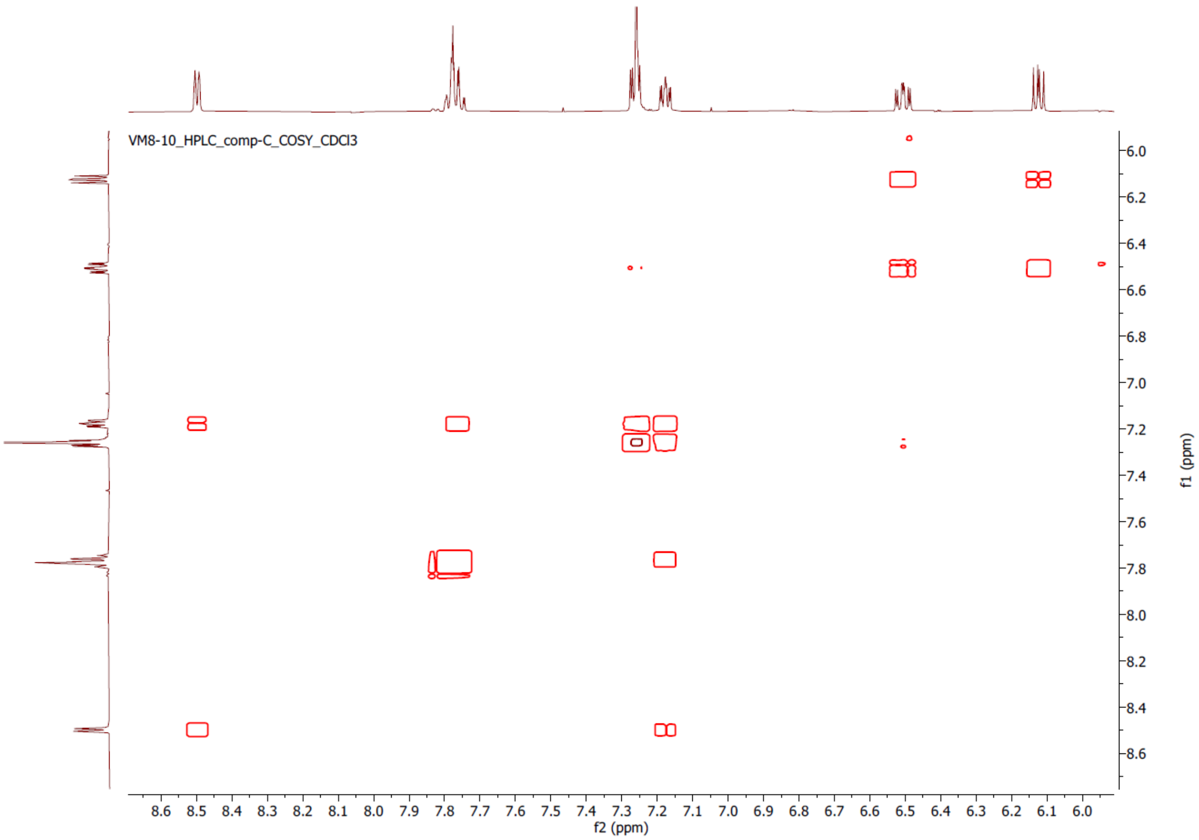

Figure S19. COSY NMR spectrum of complex **C3**.

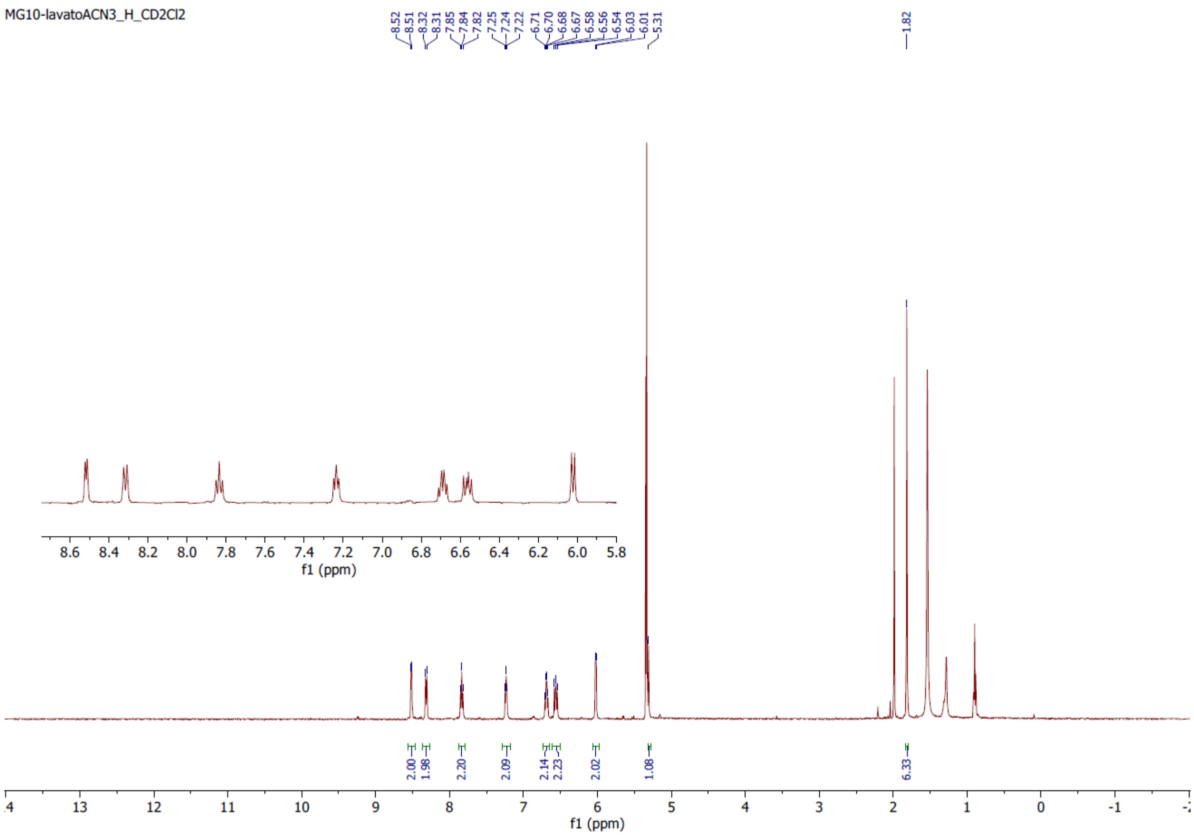

Figure S20. ^{1}H NMR spectrum of complex **C4**.

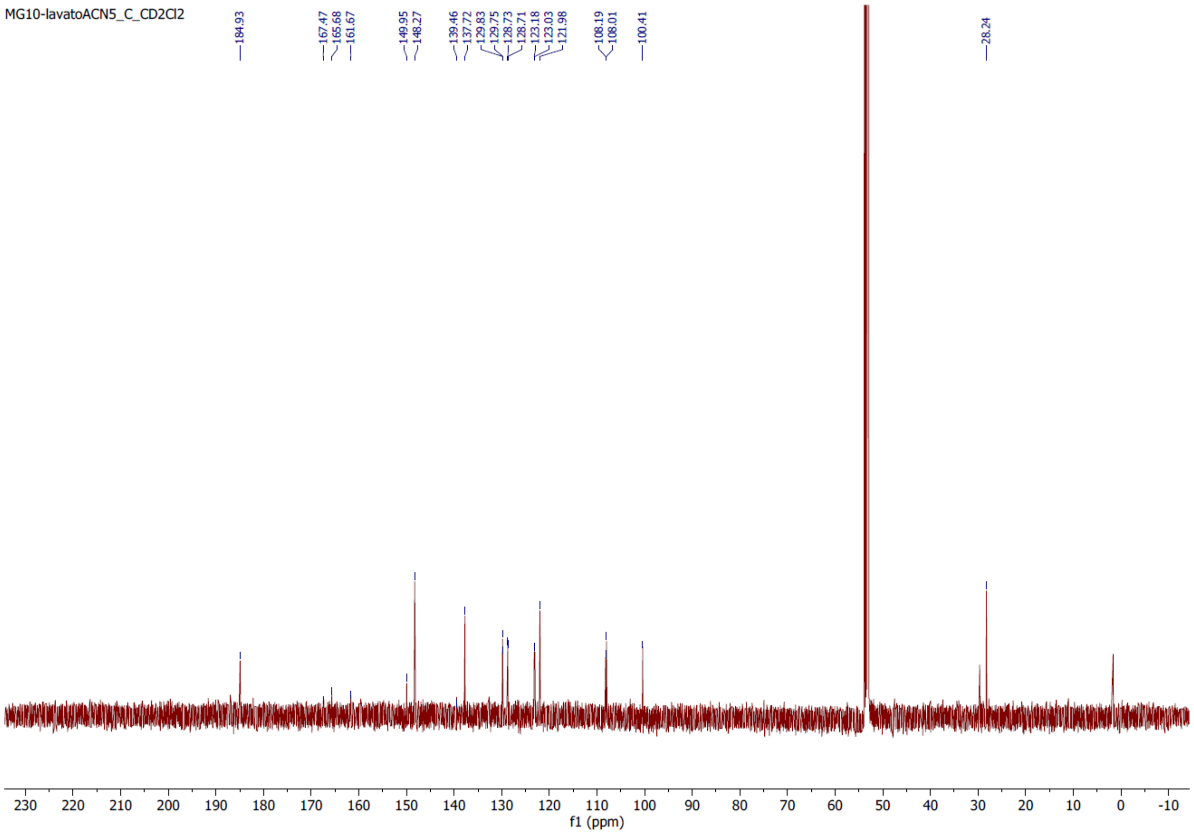

Figure S21. ^{13}C NMR spectrum of complex **C4**.

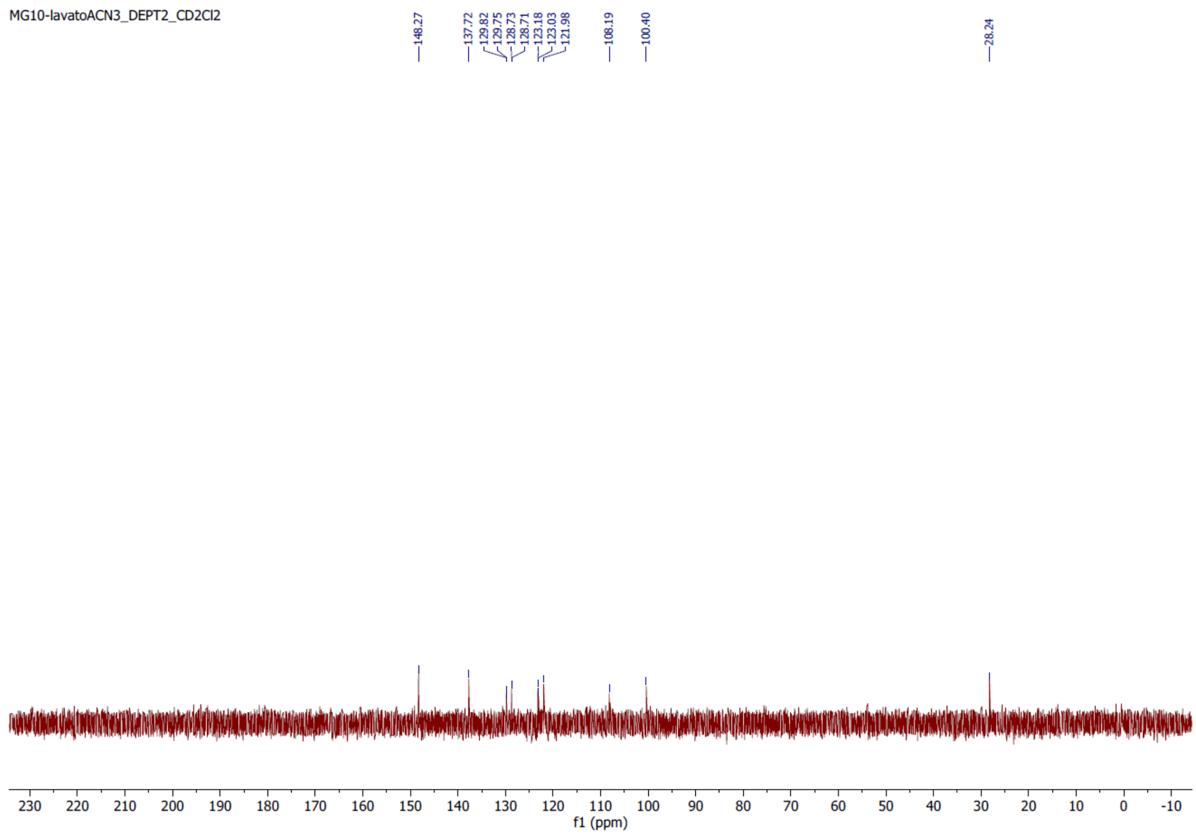

Figure S22. DEPT 135 NMR spectrum of complex **C4**.

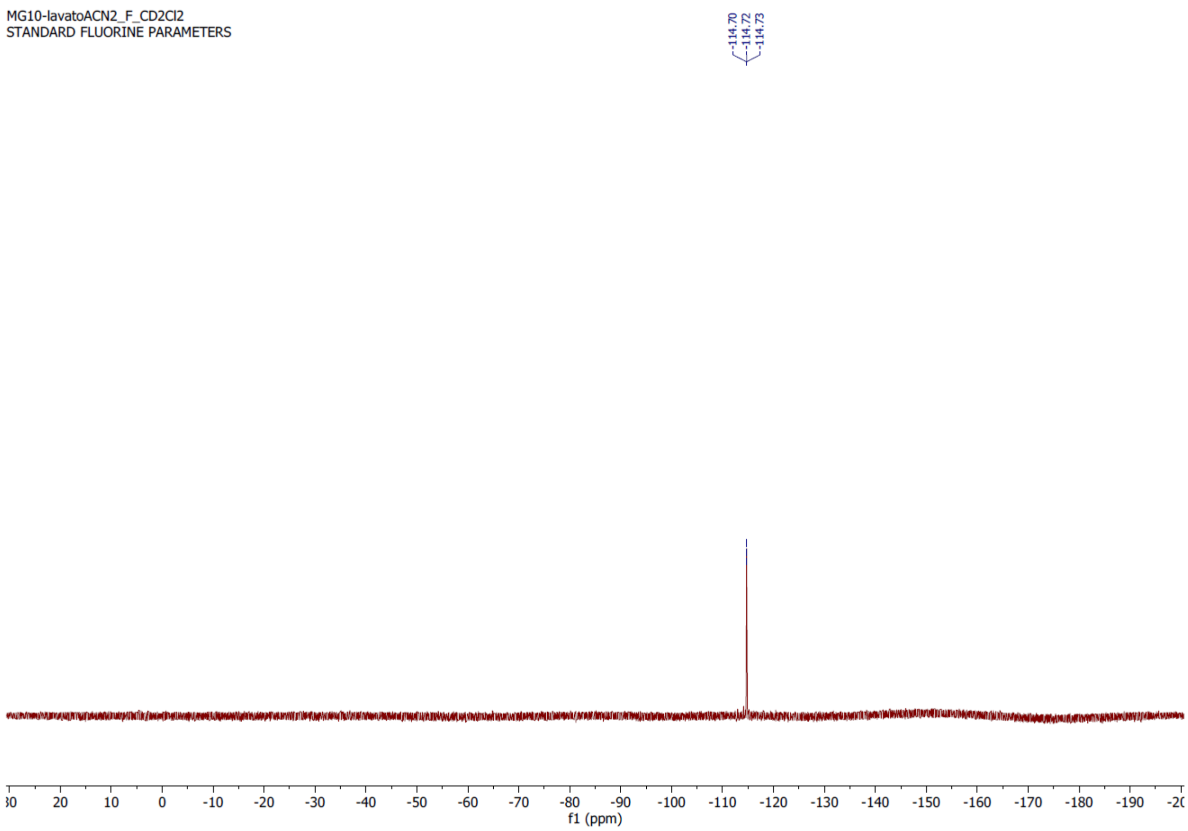

Figure S23. ^{19}F NMR spectrum of complex **C4**.

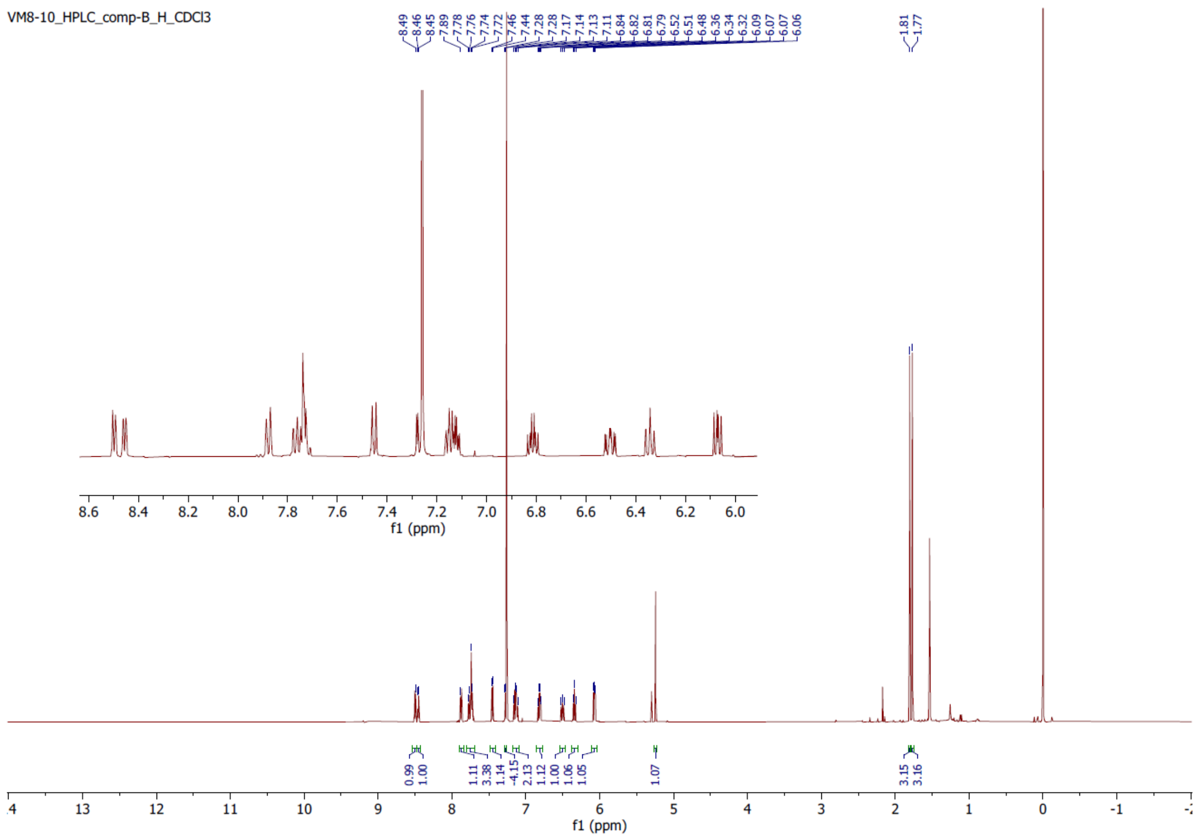

Figure S24. ^{1}H NMR spectrum of complex **C5**.

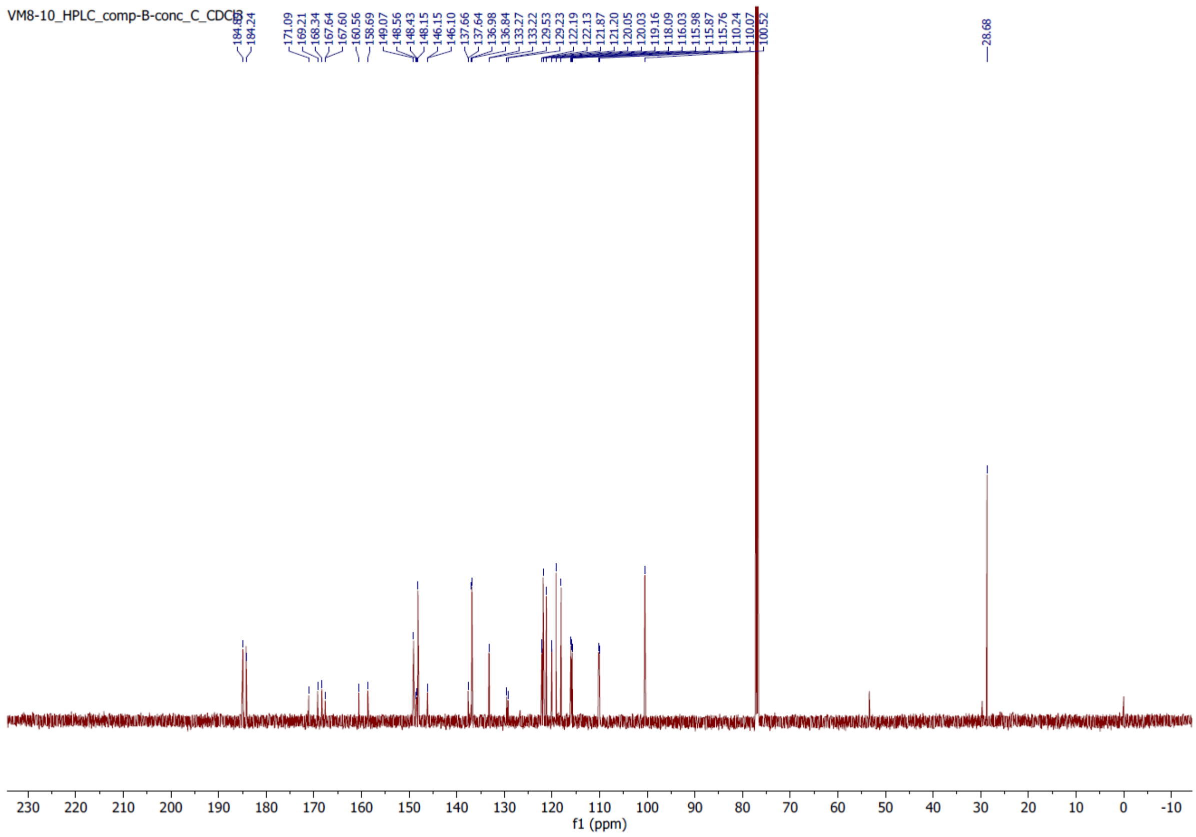

Figure S25. ^{13}C NMR spectrum of complex **C5**.

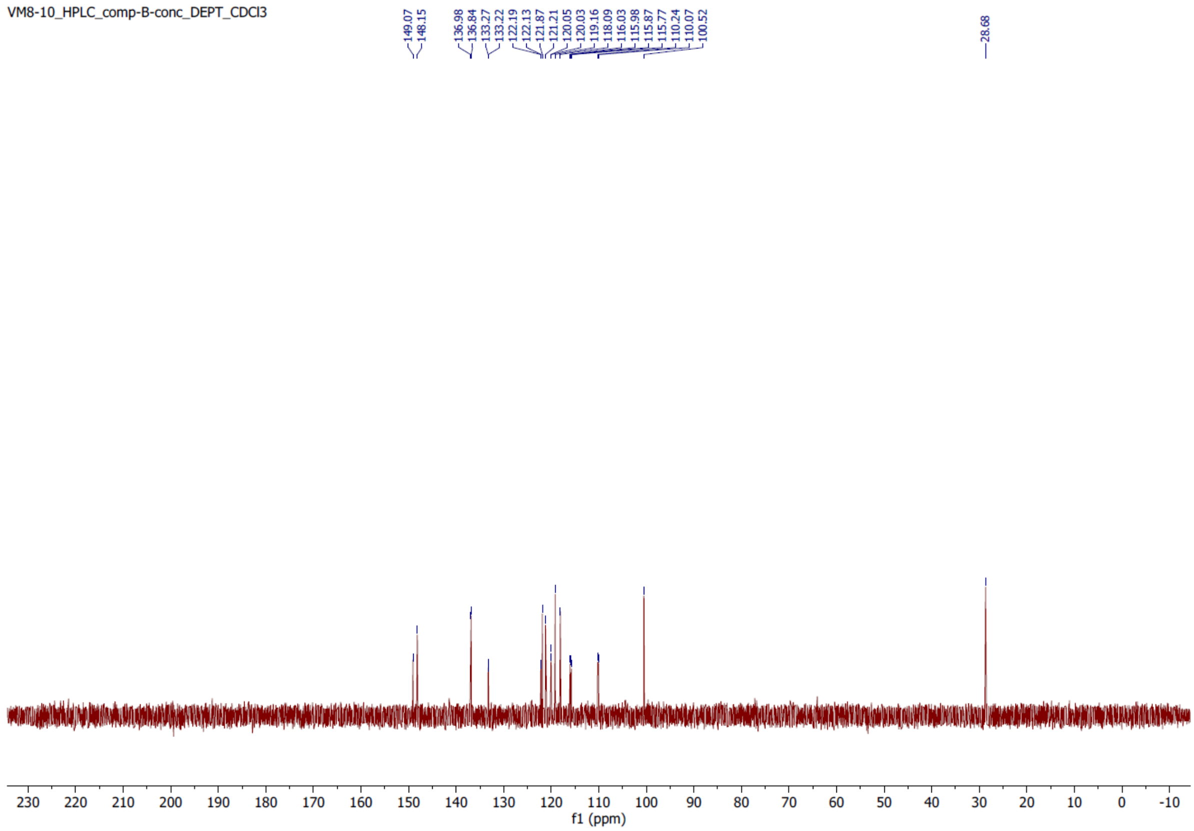

Figure S26. DEPT 135 NMR spectrum of complex **C5**.

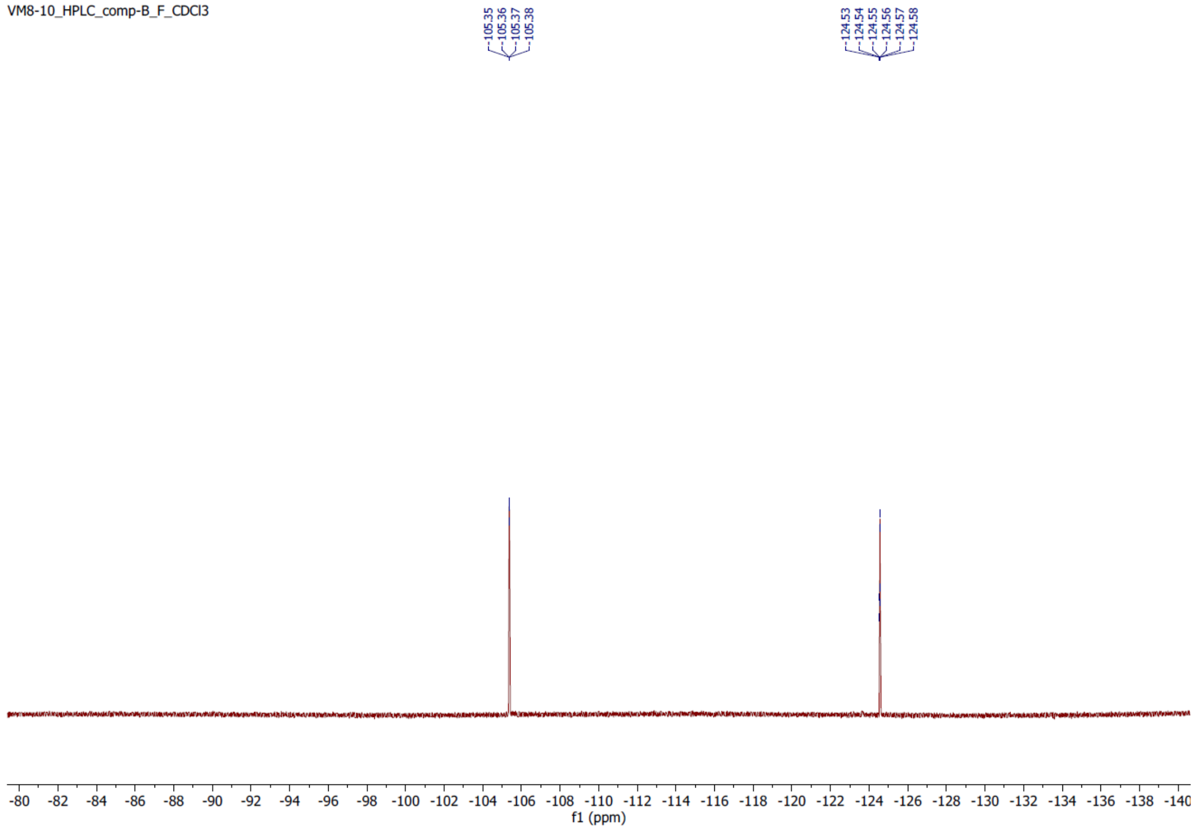

Figure S27. ^{19}F NMR spectrum of complex **C5**.

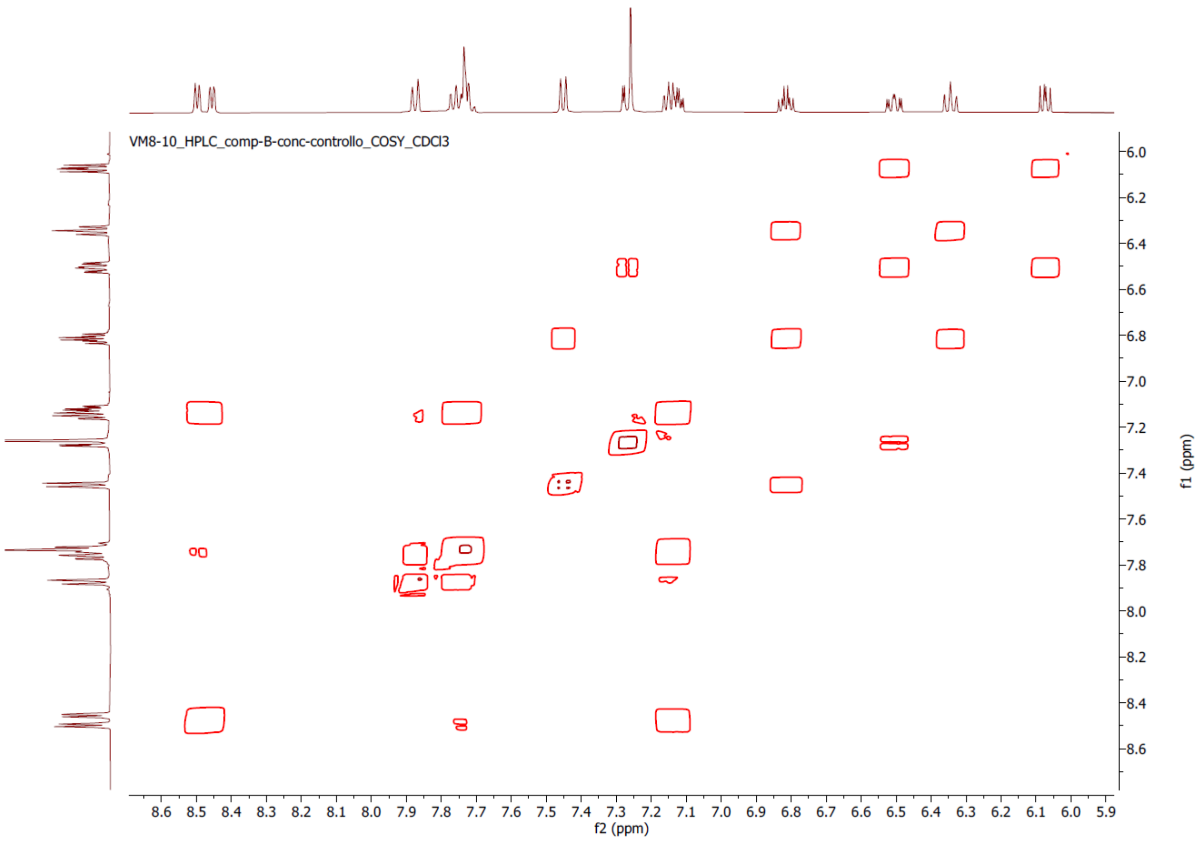

Figure S28. COSY NMR spectrum of complex **C5**.

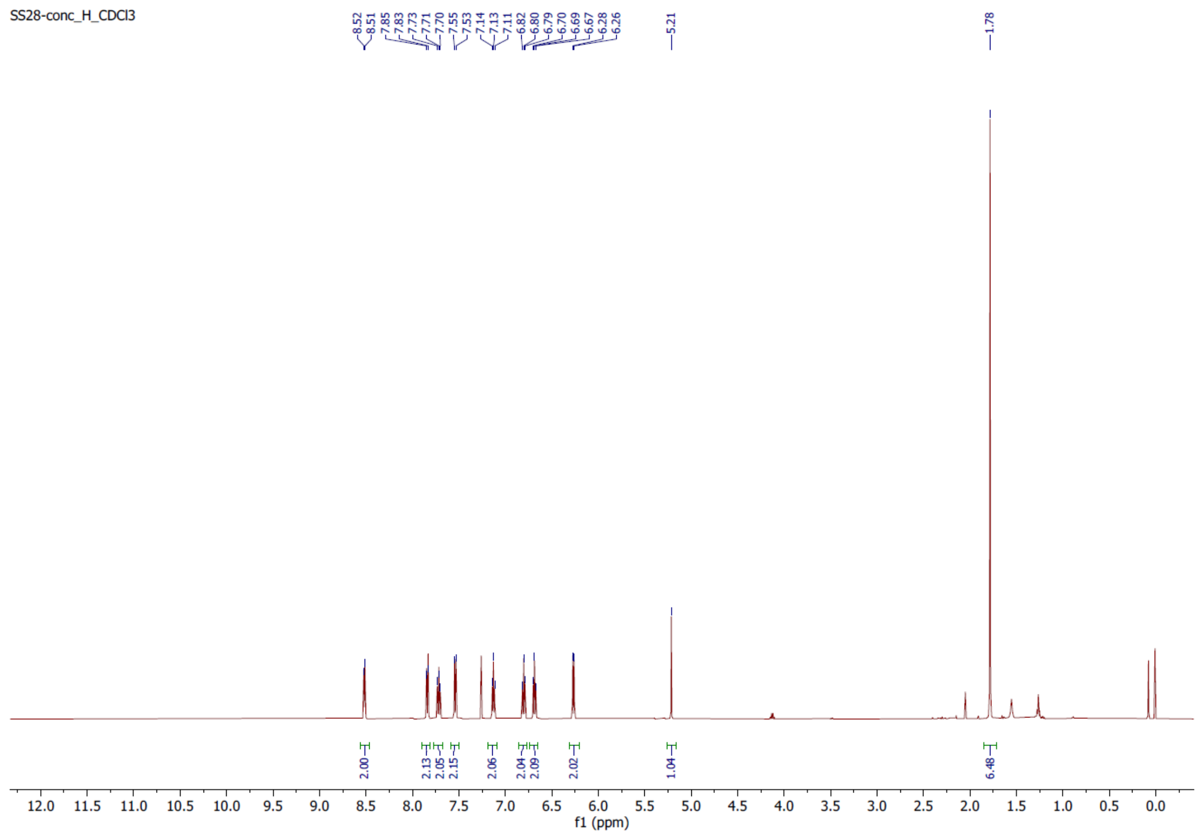

Figure S29. ^{1}H NMR spectrum of complex **C6**.

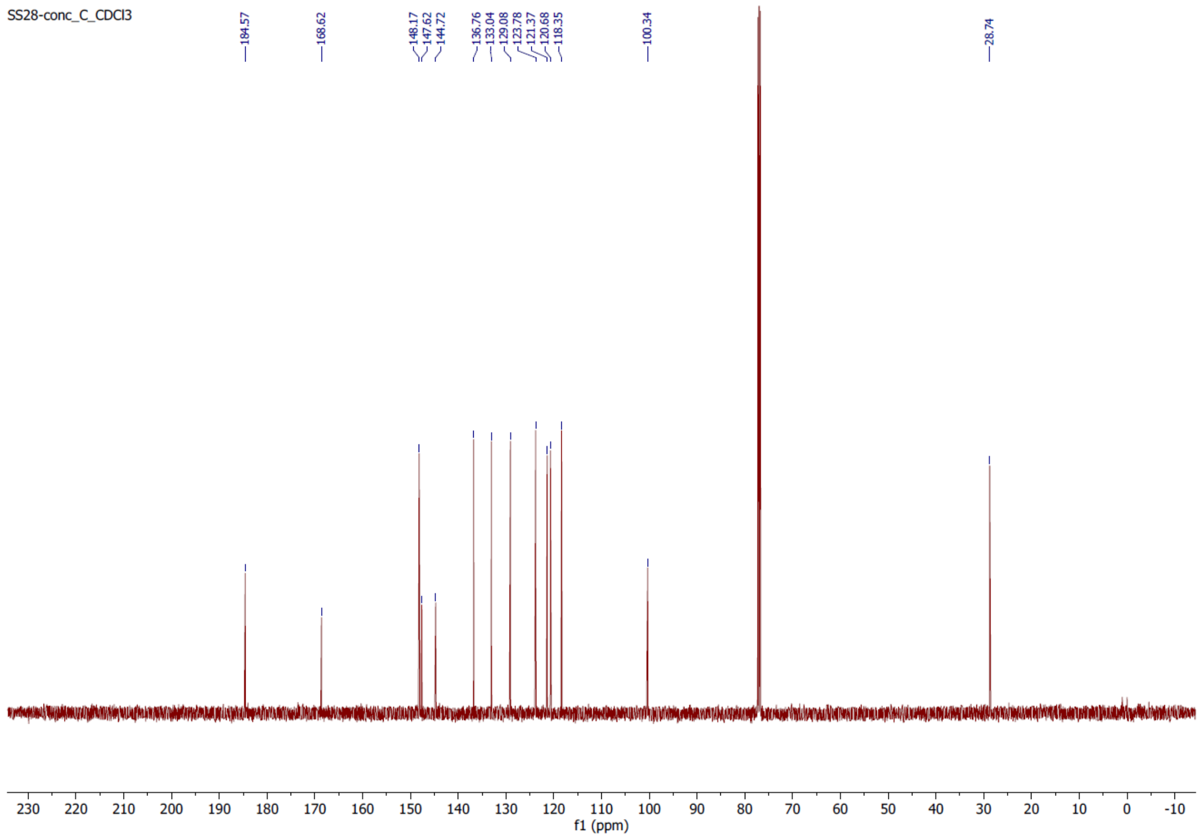

Figure S30. ^{13}C NMR spectrum of complex **C6**.

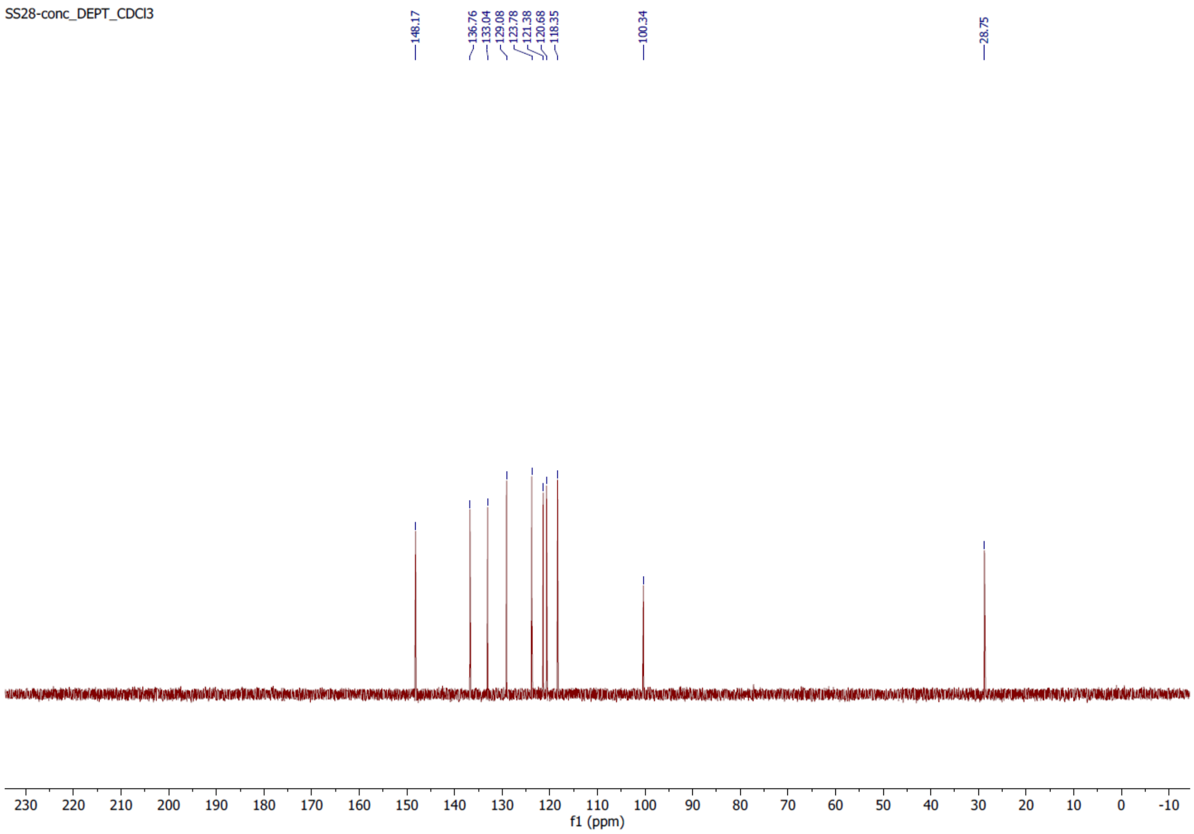

Figure S31. DEPT 135 NMR spectrum of complex **C6**.

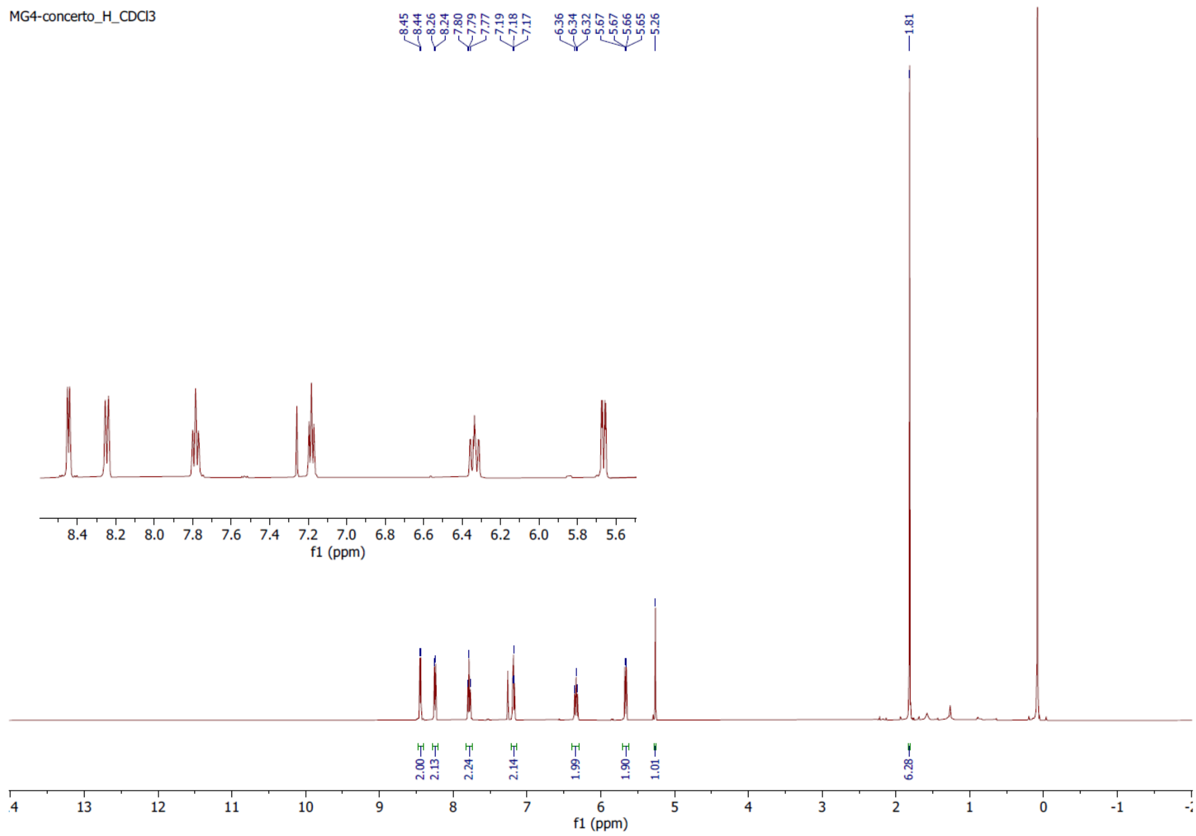

Figure S32. ^{1}H NMR spectrum of complex **C7**.

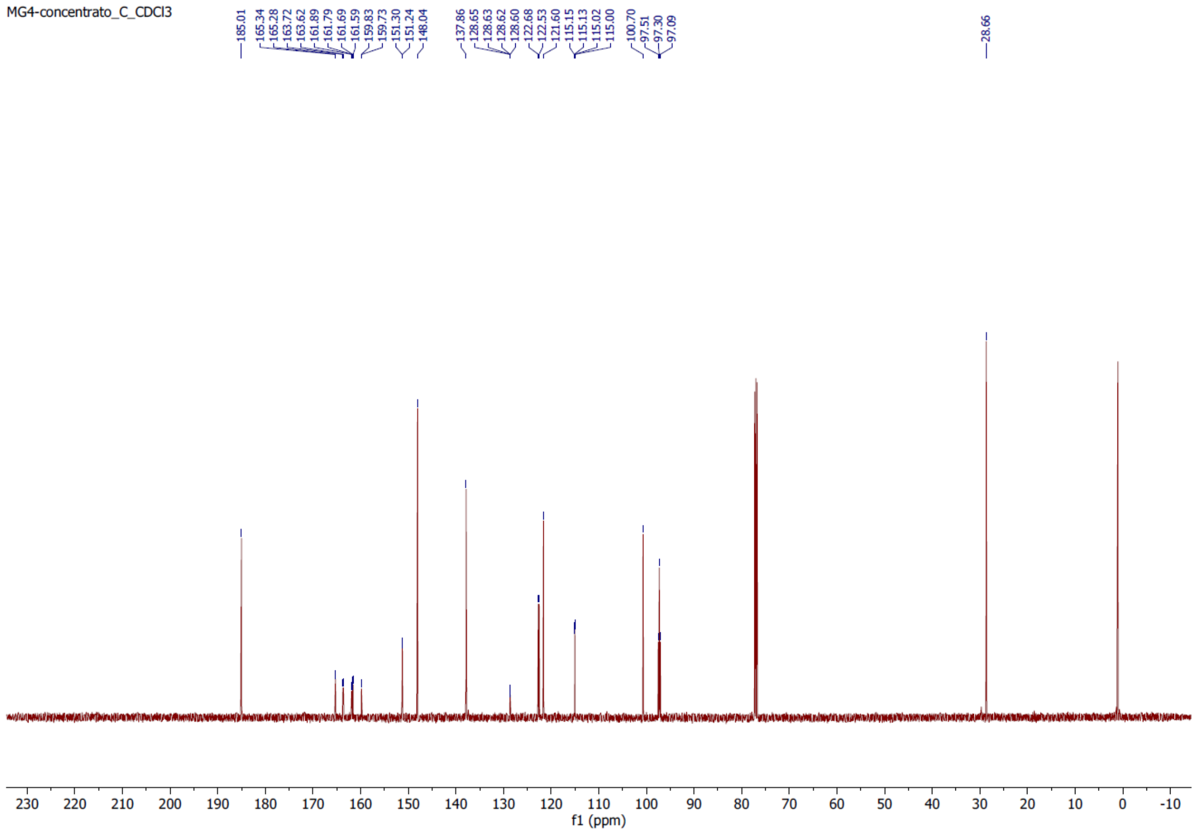

Figure S33. ^{13}C NMR spectrum of complex **C7**.

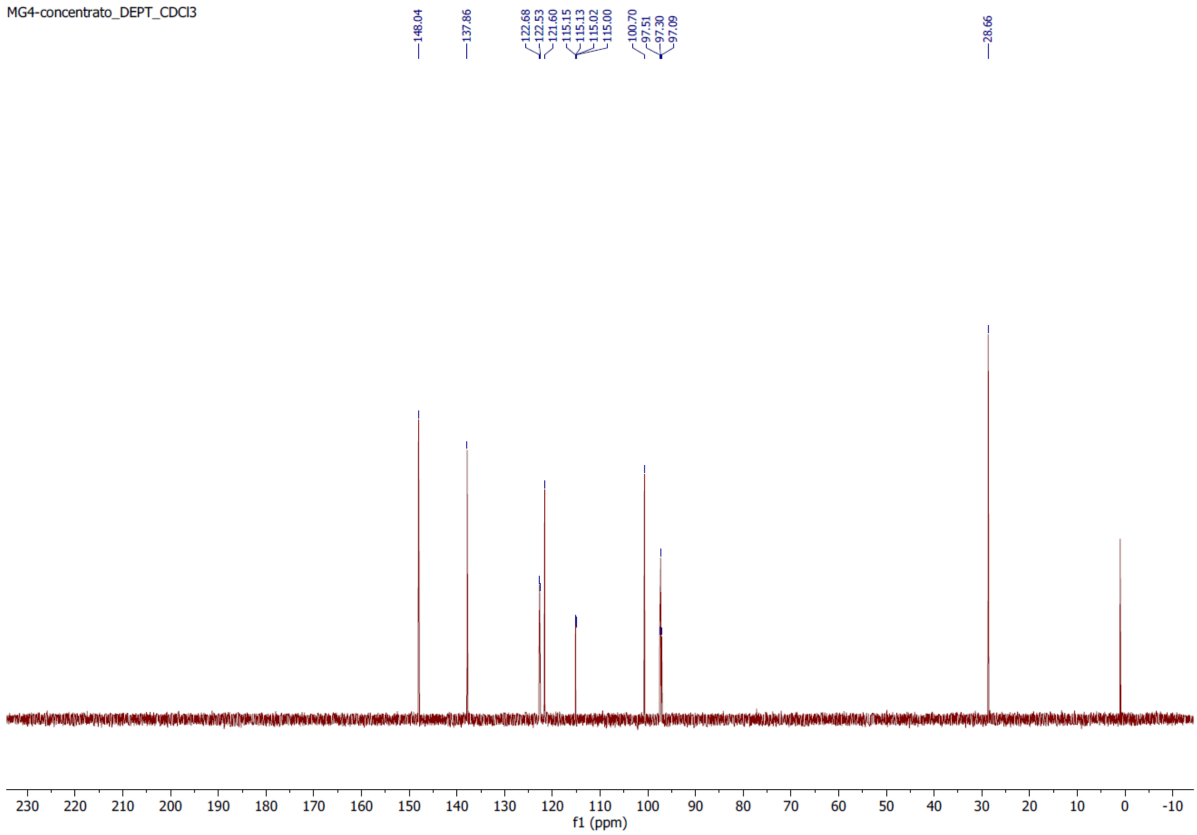

Figure S34. DEPT 135 NMR spectrum of complex **C7**.

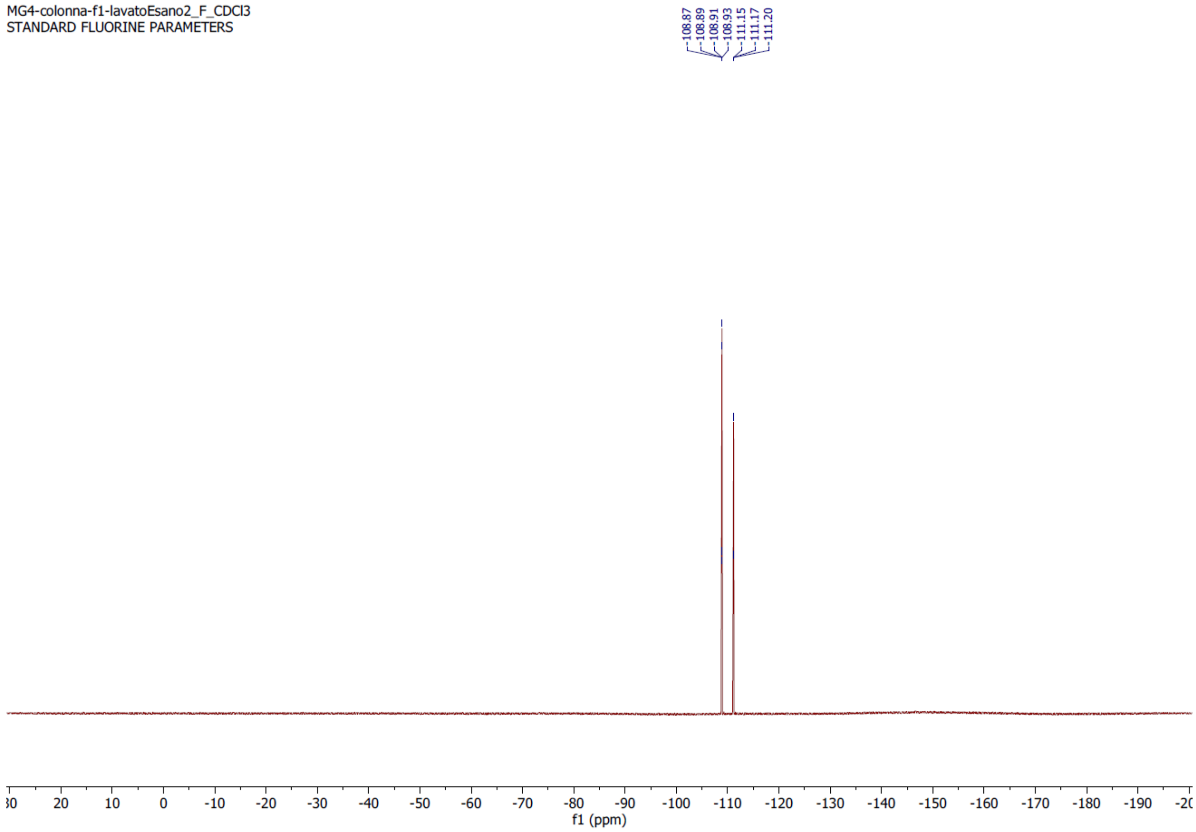

Figure S35. ^{19}F NMR spectrum of complex **C7**.

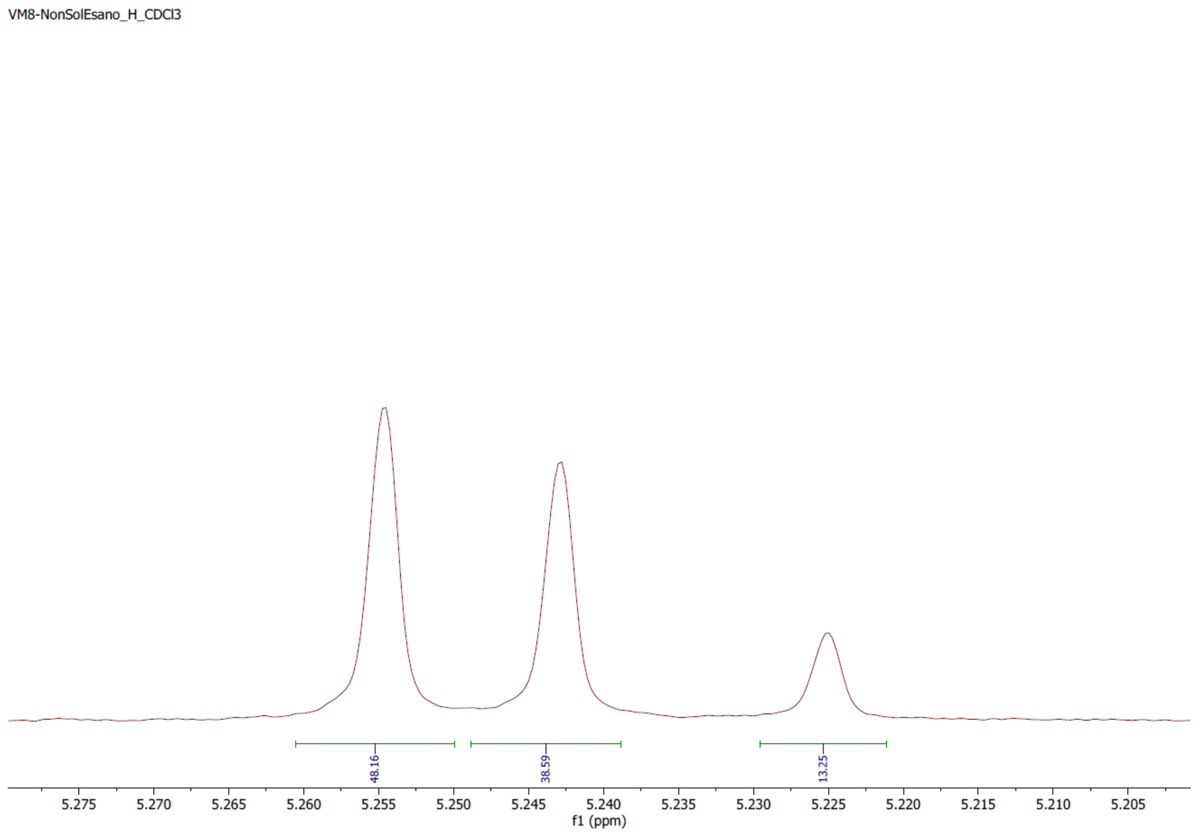

Figure S36. ^{1}H NMR spectrum of reaction crude of the synthesis of complexes **C1**, **C3** and **C5** performed at 120 °C

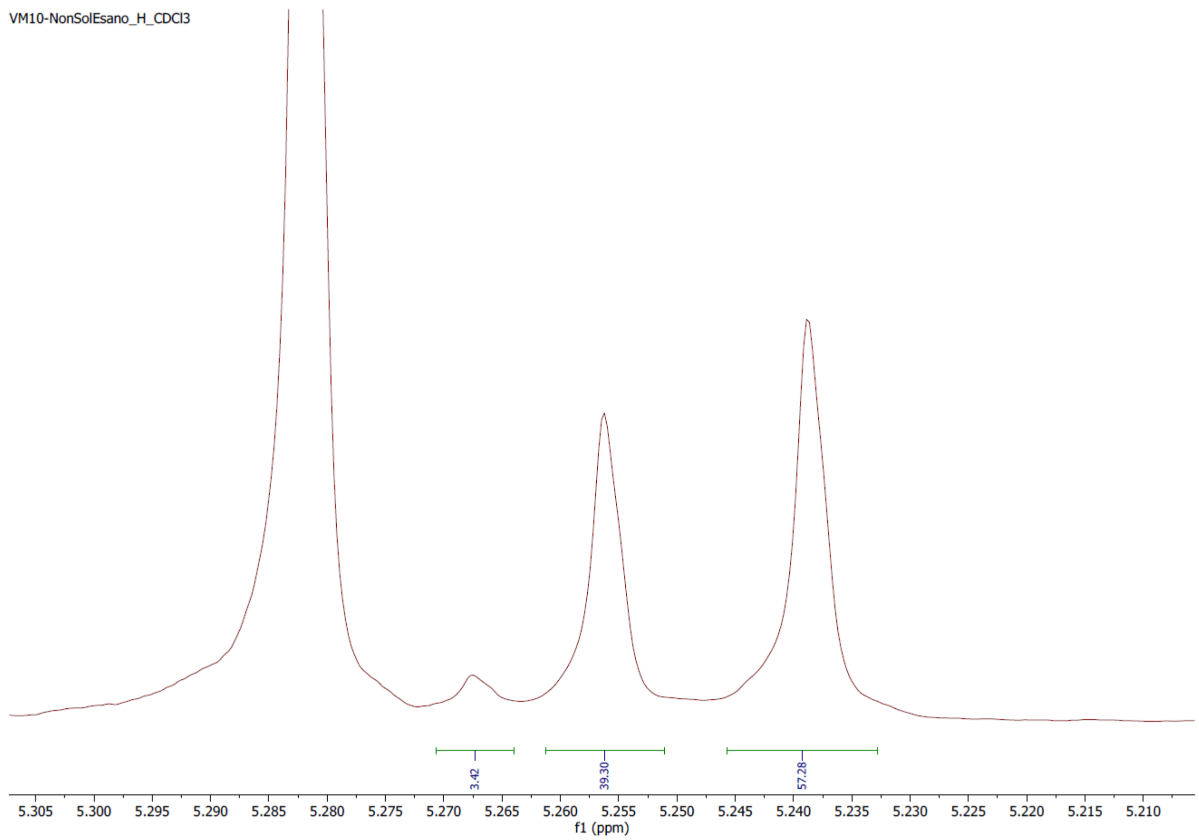

Figure S37. ^{1}H NMR spectrum of reaction crude of the synthesis of complexes **C1**, **C3** and **C5** performed at 60 °C

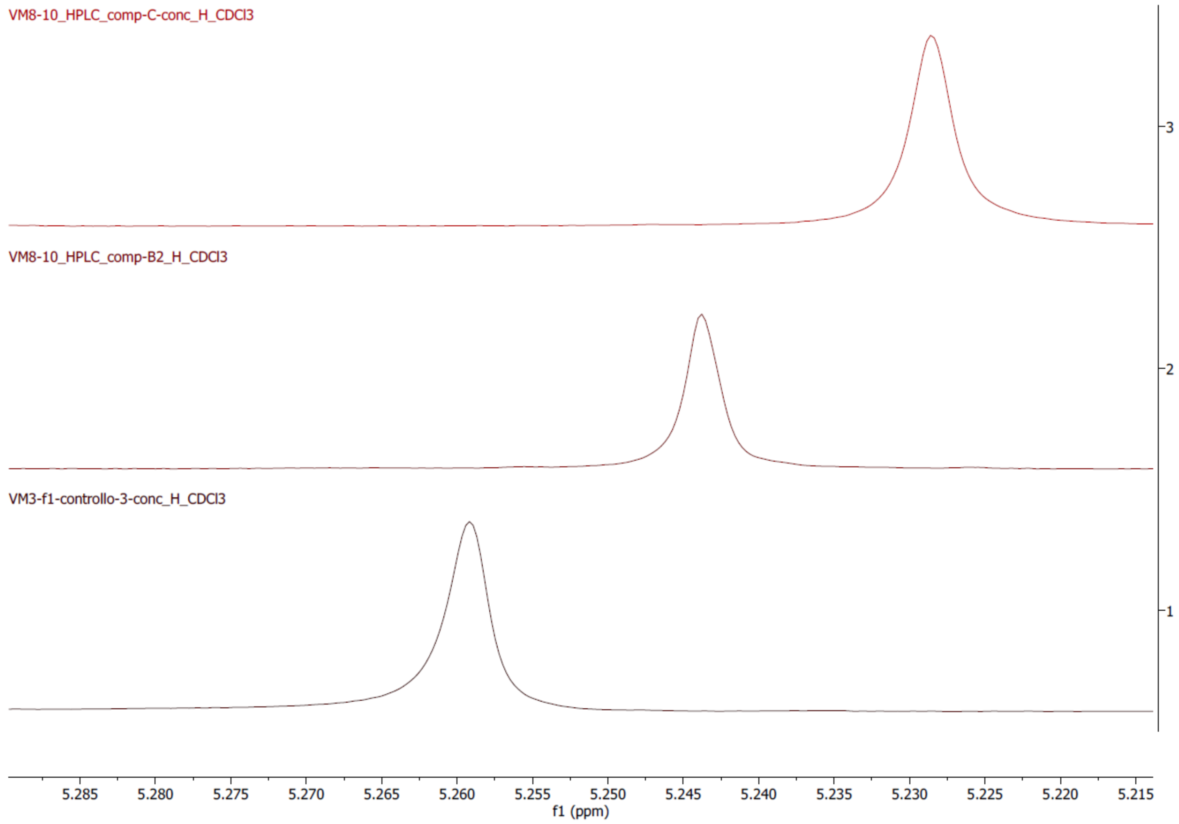

Figure S38. ^{1}H NMR spectrum shows the different CH signals (in the acetylacetonate ligand) of complex **C1** (spectrum 1), **C3** (spectrum 3) and **C5** (spectrum 2) demonstrating the effective separation of the 3 compounds.

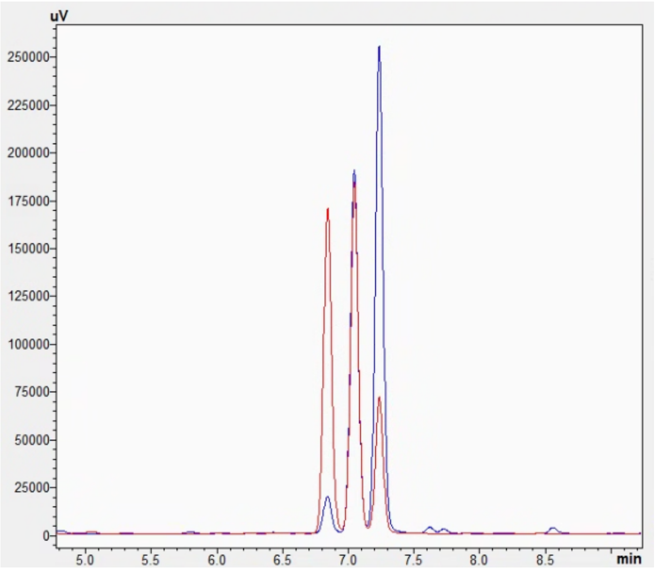

Figure S39. Analytical HPLC chromatograms (Gemini C18 column, detection at 254 nm) of the reaction crudes for the synthesis of complexes **C1**, **C3** and **C5** performed at 120 °C (red line) and 60 °C (blue line). Elution order: **C1** < **C5** < **C3**.

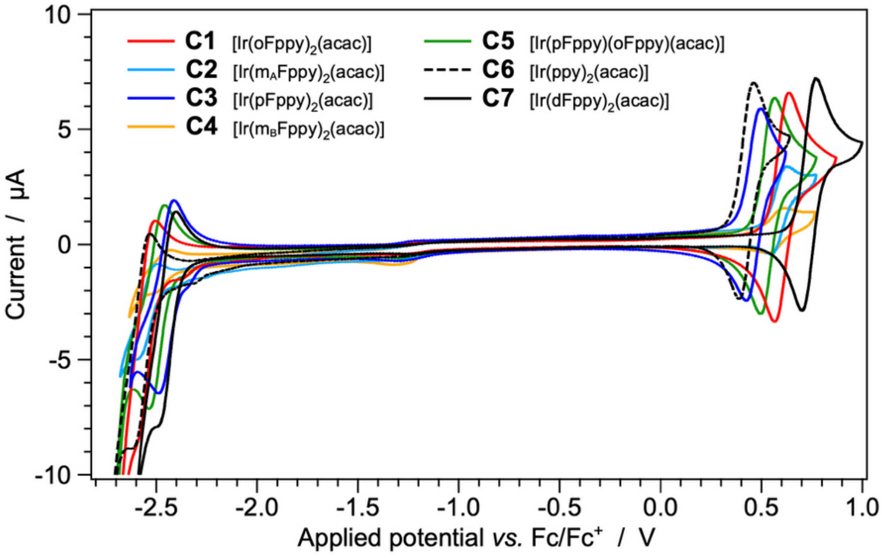

Figure S40. Cyclic voltammograms of complexes **C1**–**C7** (1.0 mM) recorded in acetonitrile solution at 298 K with a scan rate of 100 mV s^{-1}. The full reversibility of both the first oxidation and the first reduction processes is supported by the near-perfect symmetry of the anodic and cathodic waves and by a peak-to-peak separation ($\Delta E_p = E_{pa} - E_{pc}$) ranging between 68 and 72 mV.

Table S1. Calculated NTOs couples describing the lowest five triplet excitations for [Ir(oFppy)$_2$(acac)] (**C1**) in acetonitrile. The λ value is the natural transition orbital eigenvalue associated with each NTOs couple; orbital isovalue: 0.04 e$^{-1/2}$ bohr$^{-3/2}$.

	Transition energy [eV (nm)]	NTO couple hole → electron (λ)	Nature
$S_0 \rightarrow T_1$	2.67 (464)	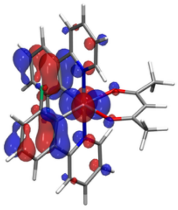 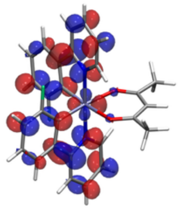 (80.4%)	mainly LC on the oFppy ligands with minor MLCT contribution
$S_0 \rightarrow T_2$	2.68 (463)	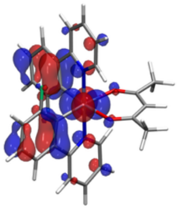 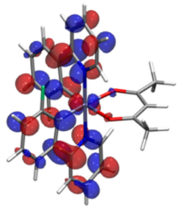 (77.4%)	mainly LC on the oFppy ligands with minor MLCT contribution
$S_0 \rightarrow T_3$	3.01 (412)	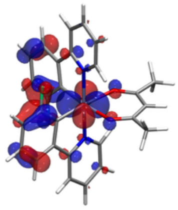 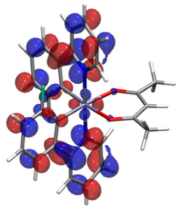 (44.7%)	predominantly MLCT from iridium to the oFppy ligands
$S_0 \rightarrow T_4$	3.03 (409)	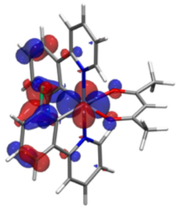 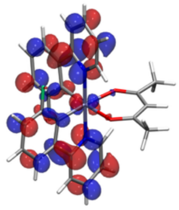 (44.4%)	predominantly MLCT from iridium to the oFppy ligands
$S_0 \rightarrow T_5$	3.17 (391)	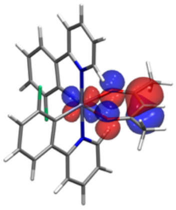 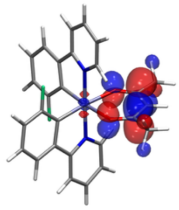 (99.6%)	mixed LC/MLCT involving the acac ancillary ligand

Table S2. Calculated NTOs couples describing the lowest five triplet excitations for [Ir(m$_A$Fppy)$_2$(acac)] (**C2**) in acetonitrile. The λ value is the natural transition orbital eigenvalue associated with each NTOs couple; orbital isovalue: 0.04 e$^{-1/2}$ bohr$^{-3/2}$.

	Transition energy [eV (nm)]	NTO couple: hole	→ electron	(λ)	Nature
$S_0 \rightarrow T_1$	2.71 (457)	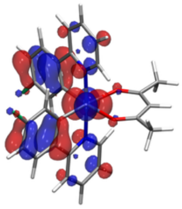	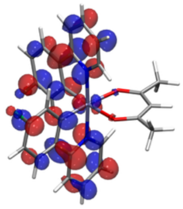	(72.3%)	mainly LC on the m$_A$Fppy ligands with minor MLCT contribution
$S_0 \rightarrow T_2$	2.74 (453)	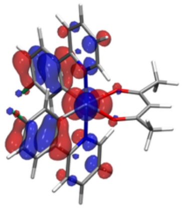	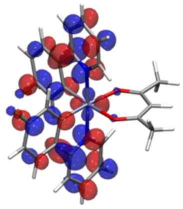	(68.1%)	mainly LC on the m$_A$Fppy ligands with minor MLCT contribution
$S_0 \rightarrow T_3$	3.07 (404)	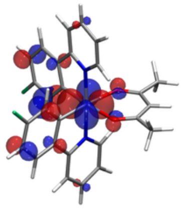	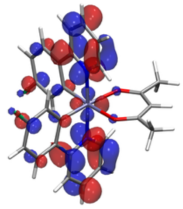	(45.3%)	predominantly MLCT from iridium to the m$_A$Fppy ligands
$S_0 \rightarrow T_4$	3.10 (401)	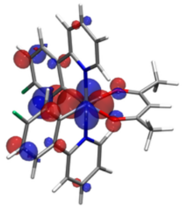	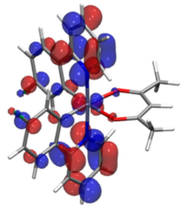	(43.4%)	predominantly MLCT from iridium to the m$_A$Fppy ligands
$S_0 \rightarrow T_5$	3.16 (392)	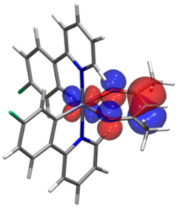	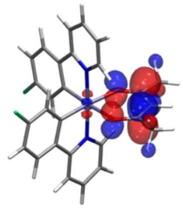	(96.6%)	mixed LC/MLCT involving the acac ancillary ligand

Table S3. Calculated NTOs couples describing the lowest five triplet excitations for [Ir(pFppy)$_2$(acac)] (**C3**) in acetonitrile. The λ value is the natural transition orbital eigenvalue associated with each NTOs couple; orbital isovalue: 0.04 e$^{-1/2}$ bohr$^{-3/2}$.

	Transition energy [eV (nm)]	NTO couple hole → electron (λ)	Nature
$S_0 \rightarrow T_1$	2.50 (495)	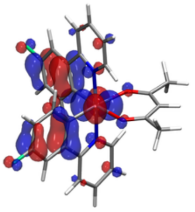 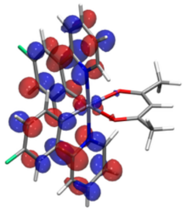 (85.6%)	mainly LC on the pFppy ligands with minor MLCT contribution
$S_0 \rightarrow T_2$	2.53 (489)	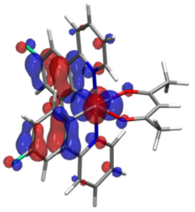 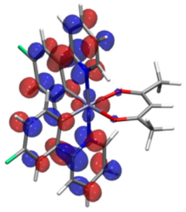 (83.9%)	mainly LC on the pFppy ligands with minor MLCT contribution
$S_0 \rightarrow T_3$	2.94 (422)	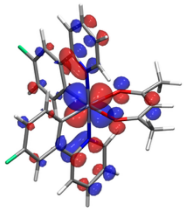 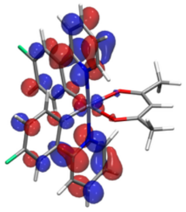 (52.4%)	predominantly MLCT from iridium to the pFppy ligands
$S_0 \rightarrow T_4$	2.96 (419)	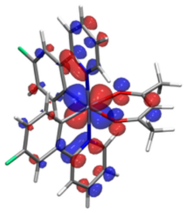 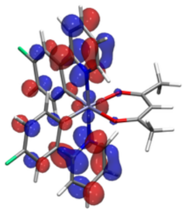 (50.6%)	predominantly MLCT from iridium to the pFppy ligands
$S_0 \rightarrow T_5$	3.16 (393)	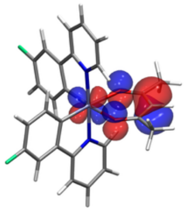 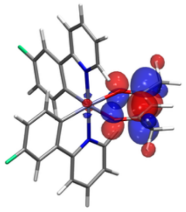 (97.4%)	mixed LC/MLCT involving the acac ancillary ligand

Table S4. Calculated NTOs couples describing the lowest five triplet excitations for [Ir(m$_B$Fppy)$_2$(acac)] (**C4**) in acetonitrile. The λ value is the natural transition orbital eigenvalue associated with each NTOs couple; orbital isovalue: 0.04 e$^{-1/2}$ bohr$^{-3/2}$.

	Transition energy [eV (nm)]	NTO couple hole	→ electron	(λ)	Nature
$S_0 \rightarrow T_1$	2.68 (462)	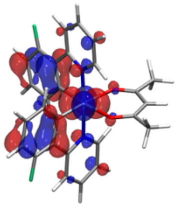	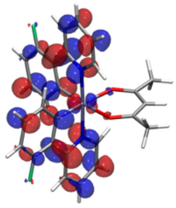	(80.1%)	mainly LC on the m$_B$Fppy ligands with minor MLCT contribution
$S_0 \rightarrow T_2$	2.71 (457)	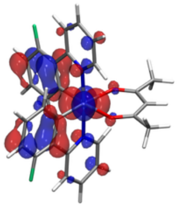	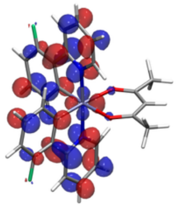	(77.8%)	mainly LC on the m$_B$Fppy ligands with minor MLCT contribution
$S_0 \rightarrow T_3$	2.99 (414)	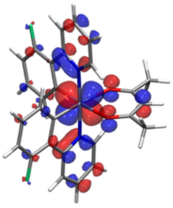	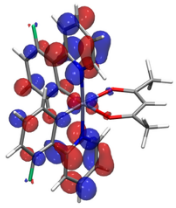	(48.0%)	predominantly MLCT from iridium to the m$_B$Fppy ligands
$S_0 \rightarrow T_4$	3.02 (411)	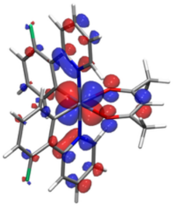	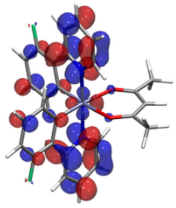	(46.2%)	predominantly MLCT from iridium to the m$_B$Fppy ligands
$S_0 \rightarrow T_5$	3.16 (392)	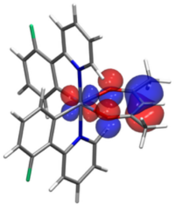	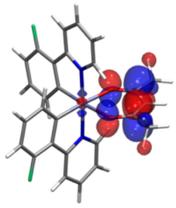	(98.9%)	mixed LC/MLCT involving the acac ancillary ligand

Table S5. Calculated NTOs couples describing the lowest five triplet excitations for [Ir(pFppy)(oFppy)(acac)] (**C5**) in acetonitrile. The λ value is the natural transition orbital eigenvalue associated with each NTOs couple; orbital isovalue: 0.04 $e^{-1/2}$ $bohr^{-3/2}$.

	Transition energy [eV (nm)]	NTO couple hole → electron (λ)	Nature
$S_0 \rightarrow T_1$	2.57 (482)	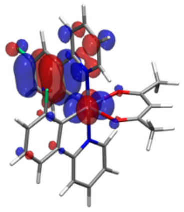 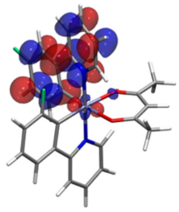 (96.4%)	mainly LC on the pFppy ligand with minor MLCT contribution
$S_0 \rightarrow T_2$	2.63 (472)	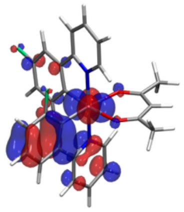 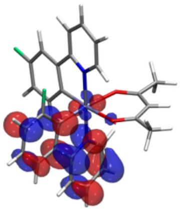 (95.6%)	mainly LC on the oFppy ligand with minor MLCT contribution
$S_0 \rightarrow T_3$	2.96 (419)	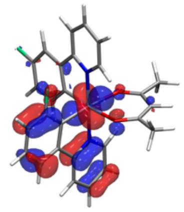 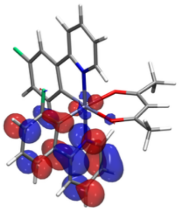 (82.5%)	predominantly MLCT from iridium to the oFppy ligand
$S_0 \rightarrow T_4$	3.02 (411)	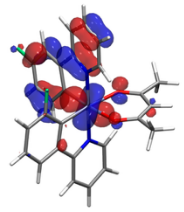 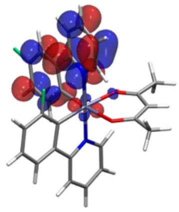 (81.7%)	predominantly MLCT from iridium to the pFppy ligand
$S_0 \rightarrow T_5$	3.17 (392)	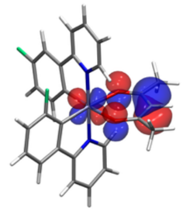 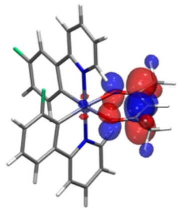 (98.7%)	mixed LC/MLCT involving the acac ancillary ligand

Table S6. Calculated NTOs couples describing the lowest five triplet excitations for [Ir(ppy)$_2$(acac)] (**C6**) in acetonitrile. The λ value is the natural transition orbital eigenvalue associated with each NTOs couple; orbital isovalue: 0.04 e$^{-1/2}$ bohr$^{-3/2}$.

	Transition energy [eV (nm)]	NTO couple hole → electron (λ)	Nature
$S_0 \rightarrow T_1$	2.61 (475)	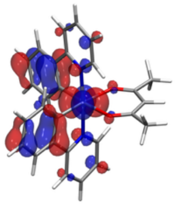 → 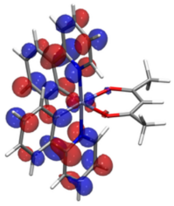 (80.9%)	mainly LC on the ppy ligands with minor MLCT contribution
$S_0 \rightarrow T_2$	2.64 (470)	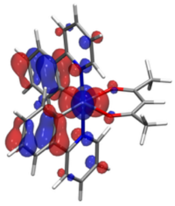 → 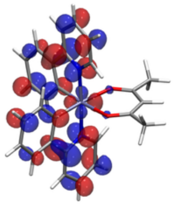 (78.5%)	mainly LC on the ppy ligands with minor MLCT contribution
$S_0 \rightarrow T_3$	2.96 (418)	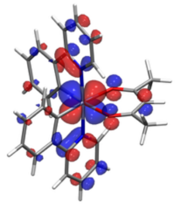 → 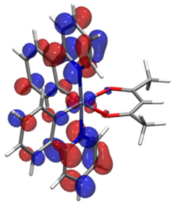 (46.9%)	predominantly MLCT from iridium to the ppy ligands
$S_0 \rightarrow T_4$	2.99 (415)	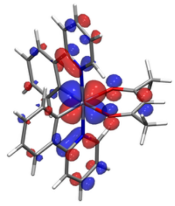 → 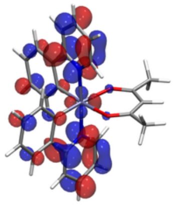 (45.7%)	predominantly MLCT from iridium to the ppy ligands
$S_0 \rightarrow T_5$	3.15 (393)	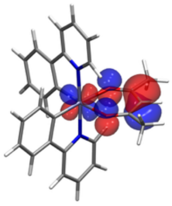 → 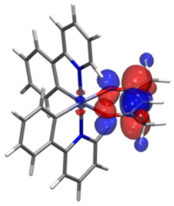 (98.5%)	mixed LC/MLCT involving the acac ancillary ligand

Table S7. Calculated NTOs couples describing the lowest five triplet excitations for [Ir(dFppy)$_2$(acac)] (**C7**) in acetonitrile. The λ value is the natural transition orbital eigenvalue associated with each NTOs couple; orbital isovalue: 0.04 e$^{-1/2}$ bohr$^{-3/2}$.

	Transition energy [eV (nm)]	NTO couple hole → electron (λ)	Nature
$S_0 \rightarrow T_1$	2.79 (444)	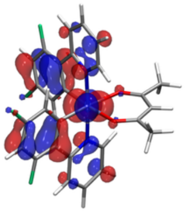 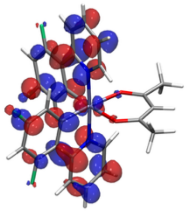 (70.3%)	mainly LC on the dFppy ligands with minor MLCT contribution
$S_0 \rightarrow T_2$	2.82 (440)	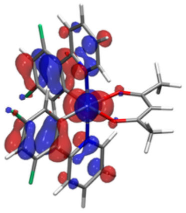 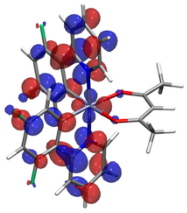 (66.1%)	mainly LC on the dFppy ligands with minor MLCT contribution
$S_0 \rightarrow T_3$	3.11 (399)	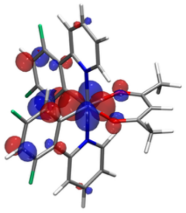 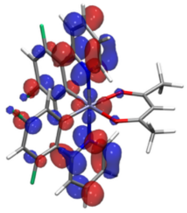 (46.4%)	predominantly MLCT from iridium to the dFppy ligands
$S_0 \rightarrow T_4$	3.13 (396)	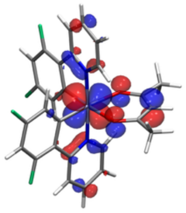 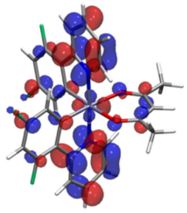 (43.9%)	predominantly MLCT from iridium to the dFppy ligands
$S_0 \rightarrow T_5$	3.17 (391)	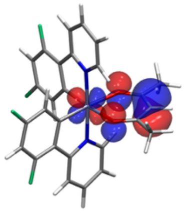 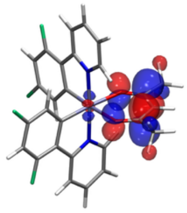 (93.5%)	mixed LC/MLCT involving the acac ancillary ligand

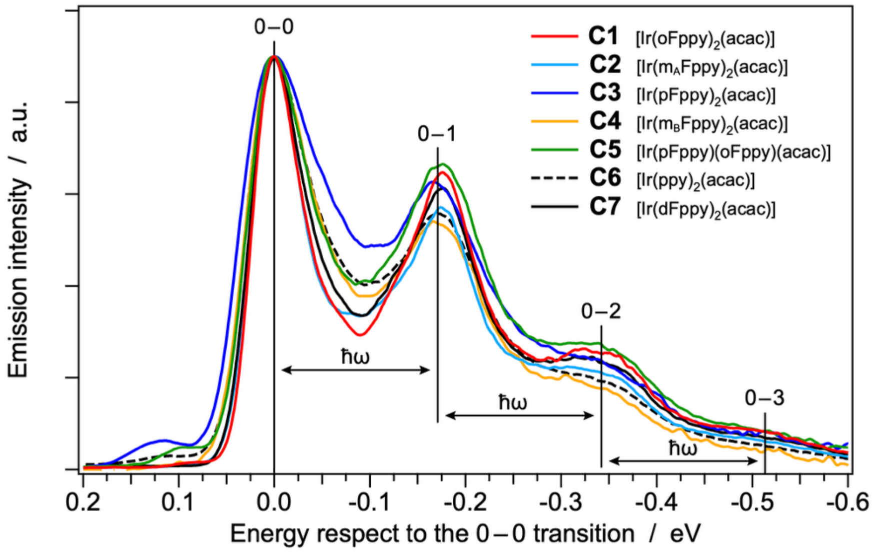

Figure S41. Normalized emission spectra of complexes **C1**–**C7** in room-temperature acetonitrile solutions, plotted as relative quanta per energy interval; all spectra have been energetically shifted to align their respective 0–0 vibronic peaks. The estimated energy of the effective vibrational mode coupling the emitting and the ground states is $\hbar\omega = (0.158 \pm 0.006)$ eV, corresponding to a vibrational frequency of (1275 ± 50) cm^{-1}, for all complexes.

Table S8. Summary of the vibronic parameters extracted from the fitting of the spectra in Figure S41. The table reports: the energy of the 0–0 transition (E_{00}); the effective vibrational mode energy ($\hbar\omega$); the Huang–Rhys factor (S), and the full width at half maximum (FWHM) used in the Gaussian broadening.

Complex		E_{00} [eV]	$\hbar\omega$ [eV]	S	FWHM [eV]
[Ir(oFppy)$_2$(acac)]	**C1**	2.553	0.160	1.132	0.103
[Ir(m$_A$Fppy)$_2$(acac)]	**C2**	2.569	0.157	1.019	0.104
[Ir(pFppy)$_2$(acac)]	**C3**	2.362	0.160	0.963	0.124
[Ir(m$_B$Fppy)$_2$(acac)]	**C4**	2.503	0.155	0.882	0.108
[Ir(pFppy)(oFppy)(acac)]	**C5**	2.453	0.160	1.105	0.113
[Ir(ppy)$_2$(acac)]	**C6**	2.451	0.156	0.922	0.111
[Ir(dFppy)$_2$(acac)]	**C7**	2.626	0.158	1.043	0.105
		**Mean**	0.158	1.01	0.110
		**Standard deviation**	0.002	0.09	0.007

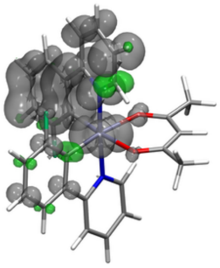

[2.542 eV]

C1

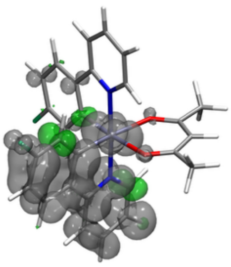

[2.607 eV]

C2

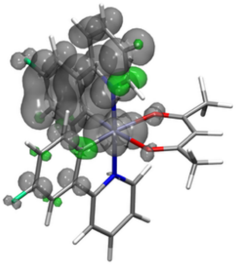

[2.352 eV]

C3

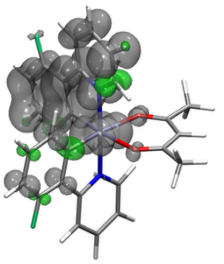

[2.567 eV]

C4

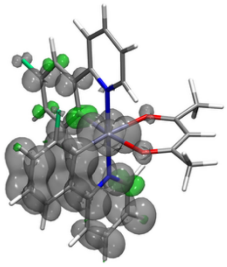

[2.481 eV]

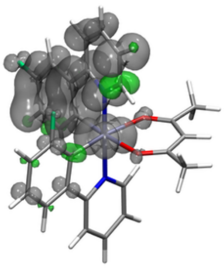

[2.418 eV]

C5

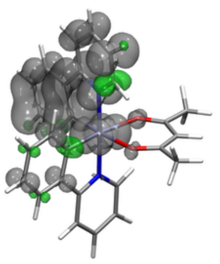

[2.483 eV]

C6

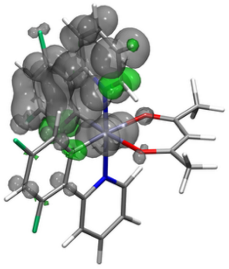

[2.699 eV]

C7

Figure S42. Spin-density distribution of the lowest triplet states of complexes **C1**–**C7** in their fully relaxed geometry, computed by spin-unrestricted DFT in acetonitrile (isovalues: 0.002 e bohr^{-3}). The adiabatic energy difference with the ground state is also reported. Notably, for the asymmetric complex **C5**, two distinct triplets have been identified; for all the other symmetrical complexes, two degenerate triplets are expected, localized on each of the equivalent cyclometallating ligands.
